# Global, regional, and national burden of older adult atopic dermatitis in 204 countries and territories worldwide

**DOI:** 10.3389/fpubh.2025.1569119

**Published:** 2025-04-02

**Authors:** Yi Ou, Xinyi Shao, Jingbo Zhang, Jin Chen

**Affiliations:** Department of Dermatology, The First Affiliated Hospital of Chongqing Medical University, Chongqing, China

**Keywords:** atopic dermatitis, the older adult, global burden of disease study, disability-adjusted life years, incidence

## Abstract

**Background:**

With the aging global population, older adult atopic dermatitis (AD) is emerging as an increasingly significant health challenge. This study aimed to evaluate the global burden of older adult AD from 1990 to 2021 and to project its change to 2050.

**Methods:**

The estimates and 95% uncertainty intervals of prevalence, incidence, and disability-adjusted life-years (DALYs) attributable to AD among individuals aged over 60 years were extracted from the Global Burden of Diseases (GBD) Study 2021. We used joinpoint regression analysis, decomposition analysis, cross-country inequality analysis, frontier analysis and prediction model to epidemiological analysis.

**Results:**

From 1990 to 2021, the global prevalence of older adult AD increased to 11,009,630 cases (95% UI: 9,915,829 to 12,170,941), even as ASRs declined, which were primarily driven by population growth. It was observed that females and 75–79 years old had higher incidence rates. SDI relative and frontier analysis exhibited that incidence, prevalence and DALYs rates were positively correlated with SDI levels, while SDI-related inequalities had a significant decrease. Predictions up to 2050 anticipated increasing older adult AD incidence, prevalence, and DALYs numbers, while only age-standardized disability-adjusted life-year rates (ASDRs) were expected to decline.

**Conclusion:**

The burden of older adult AD varied by genders, age groups, regions, countries and climatic conditions. Although the ASRs had shown a decline over time, the burden of older adult AD remained significant, especially in regions with high SDI levels. In the future, the burden of older adult AD was projected to continue rising until 2050, thereby targeted interventions and public health strategies were needed to address this trend.

## 1 Introduction

Atopic dermatitis (AD) is a chronic inflammatory skin disease primarily characterized by dry, itchy, and eczematous skin (1), significantly impacting individual social interactions and quality of life. AD arises in genetically susceptible individuals when environmental factors interact with compromised skin barrier function, leading to immune dysregulation (2). It’s the heaviest burden among skin diseases, affecting at least 230 million people globally (3). While AD is commonly observed in childhood, it can have long-term effects into adulthood. As aging intensifies, the older adult AD gradually attracts more and more attention. Among the older adult, trunk involvement is more common, and the percentage of moderate to severe AD is higher (4), which has a huge impact on their quality of life and disease burden. In addition, due to the uniqueness of the older adult population, they often have more comorbidities and are prone to worsening due to systemic medication for AD. Although children represent a key population for AD prevention and treatment, the condition in the older adult cannot be overlooked. Compared with young AD patients, decreased barrier functionalities due to aging may elevate the vulnerability to environmental stimuli, potentially resulting in systemic sensitization and predisposing older adult individuals with AD to type 2 immune reactions (5, 6). Therefore, it’s important to present the burden estimates for older adult AD.

The Global Burden of Diseases (GBD) dataset is recognized as a potent tool widely utilized in disease burden research (7–9). Currently, there is a lack of detailed global burden information specific to AD in the older adult. Therefore, our objective is to analyze the GBD database to report the global, regional, and national burdens and trends related to older adult AD from 1990 to 2021, encompassing prevalence, incidence, and disability-adjusted life years (DALYs). Furthermore, we also discuss the spatiotemporal trends associated with socioeconomic development at the national level and forecast these three indicators until 2050, which inform future public health interventions.

## 2 Methods

### 2.1 Data sources

The GBD 2021 study offers an exhaustive evaluation of health losses due to 371 diseases, injuries, and impairments across 204 countries and regions. It employs the latest epidemiological data and advanced standardized methodologies (10). The GBD database uses advanced techniques to handle missing data and correct for confounding variables. Comprehensive descriptions of the study design and methodologies of GBD studies can be found in the existing GBD literature (10). The Global Health Data Exchange query tool^1^ provides access to the data.

We sought data from the GBD study tool on incidence, prevalence, and disability-adjusted life years (DALYs) of AD in people aged 60 years and older from 1990 to 2021. To facilitate a significant comparison of rates across various global populations, age standardization was employed in the GBD study to adjust a country’s prevalence rates for different risk factors against a consistent standard population. Disability-adjusted life years (DALYs) quantified the aggregate healthy years forfeited from disease onset to demise, which were determined by summing the years lived with disability (YLDs) and the years of life lost (YLLs). YLDs were derived by multiplying the patient’s count by the time until remission or demise, incorporating the disability weight. YLLs were figured by multiplying the death toll by the standard life expectancy from a reference table. Furthermore, we also included a socio-demographic index (SDI) for analyzing the correlation between diseases and development levels, which was a comprehensive metric encompassing income, education, and fertility, and measuring the sociodemographic advancement level of a nation or region (11).

### 2.2 Statistical analyses

The burden of older adult AD was measured across different periods and segmented by region, nation, SDI, sex, and age, employing absolute numbers and age-standardized rates (ASRs) with their 95% uncertainty interval (UI). Within the GBD database, these metrics were derived based on the global population’s age distribution, determined by a specific formula: $ASR=\frac{\overset{A}{\underset{i=1}{\sum }}a_{i}w_{i}}{\overset{A}{\underset{i=1}{\sum }}w_{i}}\times 100,000$ (*a_i_*: the age-specific rate in the *i*th age group; w*_i_*: the number of people in the corresponding *i*-th age group among the standard population; A: the number of age groups). These initiatives considered variations in the demographic age structures of populations. The objective of age standardization was to mitigate the influence of demographic age structures and facilitate the comparability of research metrics. In addition, we used a joinpoint regression model (selecting the log-linear model: lny = xb) to assess rates trend (12). This model allowed us to compute the annual percent change (APC) along with its 95% confidence interval (CI), and illustrate the trend across the specified time period. Subsequently, we derived the average annual percent change (AAPC) by analyzing the trend of APC. To evaluate the correlations between the SDI and age-standardized older adult AD rates, we utilized the Spearman correlation coefficient, and statistical significance was set at *p* < 0.05. In order to gain deeper insights into the explanatory factors influencing older adult AD burden from 1990 to 2021, decomposition analyses were conducted, taking into account population size, age structure, and epidemiological shifts (13). Moreover, to examine the relationship between older adult AD burden and sociodemographic development levels, we employed frontier analysis and cross-country inequality analysis to quantify the distributive inequality of older adult AD burden across nations, in which the slope index of inequality and the concentration index represent absolute and relative gradient inequality, respectively. Additionally, we projected future trends in older adult AD burden using a Bayesian age-period-cohort (BAPC) model, incorporating integrated nested Laplace approximations. Data analysis was conducted using R software, specifically version 4.3.3.

## 3 Results

### 3.1 Global trends

In 2021, older adult AD continued to pose a significant global burden, totaling 11,009,630 cases (95% UI: 9,915,829 to 12,170,941), with a remarkable 106.98% increase since 1990 (Table 1). Notably, despite this substantial increase in case numbers, the age-standardized prevalence rates (ASPRs) exhibited a slight decline, from 1117.11 cases per 100,000 population (95% UI: 1005.84 to 1234.26) in 1990 to 1016.99 cases per 100,000 population (95% UI: 915.91 to 1124.40) in 2021.

**Table 1:** Numbers and ASRs per 100,000 cases of incidence, prevalence and DALYs of older adult atopic dermatitis in 1990 and 2021, along with the AAPC in ASRs per 100,000 cases from 1990 to 2021, categorized by global, sex, age and SDI regions.

	Incidence (95% UI)						Prevalence (95% UI)						DALYs (disability-adjusted life years) (95% UI)					
	Absolute number		ASR, per 100,000 population				Absolute number		ASR, per 100,000 population				Absolute number		ASR, per 100,000 population			
	1990	2021	1990	2021	AAPC	*P*	1990	2021	1990	2021	AAPC	*P*	1990	2021	1990	2021	AAPC	*P*
Global	665,926 (561,085 to 781,532)	1,399,503 (1,181,465 to 1,637,503)	136.94 (160.86 to 115.24)	128.22 (150.09 to 108.19)	−0.2 (−0.2 to −0.2)	<0.001	5,319,119 (4,789,963 to 5,875,954)	11,009,630 (9,915,829 to 12,170,941)	1117.11 (1234.26 to 1005.84)	1016.99 (1124.40 to 915.91)	−0.2 (−0.3 to −0.2)	<0.001	214,597 (114,429 to 359,894)	442,714 (236,073 to 743,980)	44.86 (75.27 to 23.91)	40.83 (68.63 to 21.77)	−0.1 (−0.2 to −0.1)	<0.001
**Sex**																		
Male	274,516 (231,847 to 321,317)	609,573 (514,888 to 714,646)	127 (107 to 148)	121 (102 to 142)	−0.1 (−0.2 to −0.1)	<0.001	2,049,556 (1,840,680 to 2,265,063)	4,569,779 (4,110,761 to 5,056,828)	980 (880 to 1,083)	925 (832 to 1,023)	−0.2 (−0.2 to−0.2)	<0.001	83,229 (44,505 to 139,923)	185,012 (98,356 to 311,632)	39 (21 to 66)	37 (20 to 63)	−0.2 (−0.2 to −0.2)	<0.001
Female	391,410 (329,616 to 460,672)	789,930 (664,562 to 926,929)	146 (122 to 171)	135 (113 to 158)	−0.2 (−0.3 to −0.2)	<0.001	3,269,562 (2,947,244 to 3,610,161)	6,439,850 (5,807,580 to 7,127,615)	1,229 (1,108 to 1,357)	1,099 (991 to 1,217)	−0.4 (−0.4 to −0.3)	<0.001	131,368 (70,022 to 220,141)	257,702 (137,531 to 432,746)	49 (26 to 83)	44 (23 to 74)	−0.4 (−0.4 to −0.3)	<0.001
**Age**																		
60–64	364,038 (306,627 to 426,836)	372,195 (313,299 to 435,812)	94 (67 to 133)	93 (66 to 131)	−0.1 (−0.2 to 0)	0.006	2,698,234 (2,437,894 to 2,976,090)	2,751,922 (2,482,504 to 3,031,913)	1,182 (1,041 to 1,327)	1,134 (997 to 1,268)	−0.2 (−0.3 to −0.1)	<0.001	112,073 (61,202 to 188,761)	114,013 (62,979 to 191,041)	43 (23 to 71)	41 (22 to 68)	−0.2 (−0.3 to −0.1)	0.001
65–69	329,072 (278,734 to 386,373)	346,531 (293,307 to 406,797)	100 (79 to 123)	96 (77 to 118)	−0.3 (−0.3 to −0.2)	<0.001	2,399,708 (2,168,086 to 2,646,356)	2,509,565 (2,266,165 to 2,768,562)	1,360 (1,231 to 1,501)	1,260 (1,138 to 1,392)	−0.4 (−0.4 to −0.3)	<0.001	98,283 (52,244 to 163,975)	102,573 (53,764 to 171,328)	50 (27 to 85)	47 (25 to 78)	−0.4 (−0.5 to −0.3)	<0.001
70–74	268,647 (228,084 to 310,083)	293,987 (249,386 to 339,347)	120 (99 to 144)	114 (94 to 135)	−0.2 (−0.2 to −0.1)	<0.001	1,986,040 (1,784,681 to 2,194,971)	2,169,341 (1,948,106 to 2,398,667)	1,467 (1,317 to 1,622)	1,326 (1,191 to 1,472)	−0.2 (−0.3 to−0.2)	<0.001	79,968 (43,309 to 133,776)	87,214 (46,586 to 147,731)	56 (30 to 95)	50 (27 to 86)	−0.2 (−0.3 to −0.2)	<0.001
75–79	188,852 (161,230 to 220,906)	192,891 (164,860 to 225,113)	147 (123 to 173)	136 (115 to 160)	−0.3 (−0.4 to −0.3)	<0.001	1,530,447 (1,372,526 to 1,706,903)	1,552,590 (1,392,081 to 1,731,337)	1,460 (1,319 to 1,608)	1,293 (1,170 to 1,427)	−0.4 (−0.5 to−0.4)	<0.001	60,547 (31,395 to 101,159)	61,366 (31,732 to 102,026)	57 (30 to 96)	50 (26 to 85)	−0.4 (−0.5 to−0.3)	<0.001
80–84	114,882 (96,595 to 134,907)	119,497 (100,449 to 140,456)	161 (137 to 187)	146 (125 to 171)	−0.3 (−0.3 to −0.2)	<0.001	1,092,693 (988,230 to 1,206,671)	1,132,721 (1,024,555 to 1,249,483)	1,342 (1,201 to 1,498)	1,177 (1,056 to 1,313)	−0.4 (−0.4 to−0.4)	<0.001	42,475 (22,275 to 71,893)	43,959 (23,102 to 74,767)	53 (27 to 89)	47 (24 to 77)	−0.4 (−0.6 to −0.3)	<0.001
85–89	49,512 (40,685 to 58,569)	52,094 (42,845 to 61,675)	151 (128 to 174)	143 (121 to 165)	−0.2 (−0.2 to −0.1)	<0.001	580,099 (521,390 to 643,990)	606,339 (544,442 to 672,828)	1,130 (1,016 to 1,249)	1,054 (946 to 1,165)	−0.3 (−0.4 to−0.3)	<0.001	22,018 (11,768 to 37,952)	23,000 (12,254 to 39,430)	46 (24 to 76)	42 (23 to 72)	−0.3 (−0.5 to −0.2)	<0.001
89–94	16,311 (12,942 to 20,039)	17,253 (13,723 to 21,161)	136 (115 to 162)	126 (106 to 147)	−0.1 (−0.2 to 0)	0.005	214,557 (194,503 to 237,156)	225,336 (203,650 to 249,064)	1,012 (913 to 1,114)	910 (822 to 1,004)	−0.3 (−0.3 to−0.2)	<0.001	7,963 (4,308 to 13,411)	8,350 (4,479 to 13,952)	41 (22 to 69)	37 (19 to 62)	−0.2 (−0.4 to 0)	0.014
95+	4,590 (3,262 to 6,500)	5,055 (3,595 to 7,143)	121 (102 to 143)	116 (98 to 136)	0	<0.001	56,087 (49,179 to 62,724)	61,816 (54,324 to 69,088)	920 (830 to 1,013)	860 (776 to 947)	−0.1 (−0.2 to −0.1)	<0.001	2,034 (1,062 to 3,358)	2,239 (1,177 to 3,704)	38 (21 to 64)	36 (20 to 60)	−0.2 (−0.3 to 0)	0.038
**SDI levels**																		
Low SDI	18,830 (15,670 to 22,388)	41,802 (34,719 to 49,533)	72 (60 to 86)	73 (60 to 86)	0	<0.001	124,057 (109,820 to 139,218)	275,465 (244,056 to 309,253)	482 (427 to 541)	484 (429 to 543)	0	<0.001	5,095 (2,707 to 8,772)	11,334 (5,992 to 19,707)	20 (10 to 34)	20 (10 to 35)	0	<0.001
Low-middle SDI	59,136 (50,064 to 69,234)	147,181 (124,339 to 172,414)	84 (71 to 99)	85 (72 to 100)	0	<0.001	399,627 (358,296 to 444,644)	1,000,286 (896,039 to 1,111,658)	579 (519 to 644)	587 (526 to 652)	0	<0.001	16,279 (8,674 to 27,847)	40,652 (21,689 to 69,643)	23 (12 to 40)	24 (13 to 41)	0 (0 to 0.1)	<0.001
Middle SDI	124,642 (106,126 to 144,933)	341,041 (292,072 to 395,931)	103 (88 to 120)	102 (88 to 119)	0	<0.001	832,026 (747,320 to 920,745)	2,292,079 (2,066,115 to 2,538,994)	698 (627 to 772)	693 (625 to 768)	0	<0.001	34,160 (18,178 to 58,203)	93,645 (49,821 to 159,741)	28 (15 to 49)	28 (15 to 48)	0	<0.001
High-middle SDI	164,647 (138,787 to 193,217)	315,410 (266,226 to 369,970)	130 (110 to 153)	123 (103 to 144)	−0.2 (−0.2 to −0.2)	<0.001	1,255,338 (1,125,203 to 1,395,659)	2,356,247 (2,109,618 to 2,619,550)	1,011 (906 to 1,124)	922 (825 to 1,025)	−0.3 (−0.3 to−0.3)	<0.001	50,732 (27,155 to 85,255)	95,294 (51,023 to 161,216)	41 (22 to 68)	37 (20 to 63)	−0.3 (−0.3 to −0.2)	<0.001
High SDI	298,031 (249,031 to 352,698)	552,946 (461,627 to 654,770)	205 (172 to 243)	203 (170 to 241)	0	<0.001	2,703,511 (2,436,402 to 2,984,645)	5,077,483 (4,573,891 to 5,606,286)	1,866 (1,682 to 2,059)	1,840 (1,658 to 2,031)	0 (−0.1 to 0)	<0.001	108,146 (57,814 to 180,639)	201,463 (107,410 to 335,806)	75 (40 to 125)	73 (39 to 122)	−0.1 (−0.1 to 0)	<0.001

AAPC, average annual percent change; ASRs, age-standardized rates; DALYs, disability-adjusted life-years; SDI, Socio-demographic Index; UI, uncertainty interval.

Regarding incidence, the global number of new older adult AD cases reached 1,399,503 in 2021 (95% UI: 1,181,465 to 1,637,503), representing a 110.16% surge compared to 1990. The age-standardized incidence rates (ASIRs) of older adult AD declined from 136.94 cases per 100,000 people (95% UI: 115.24 to 160.86) in 1990 to 128.22 cases per 100,000 (95% UI: 108.19 to 150.09) in 2021.

**Figure 1:**
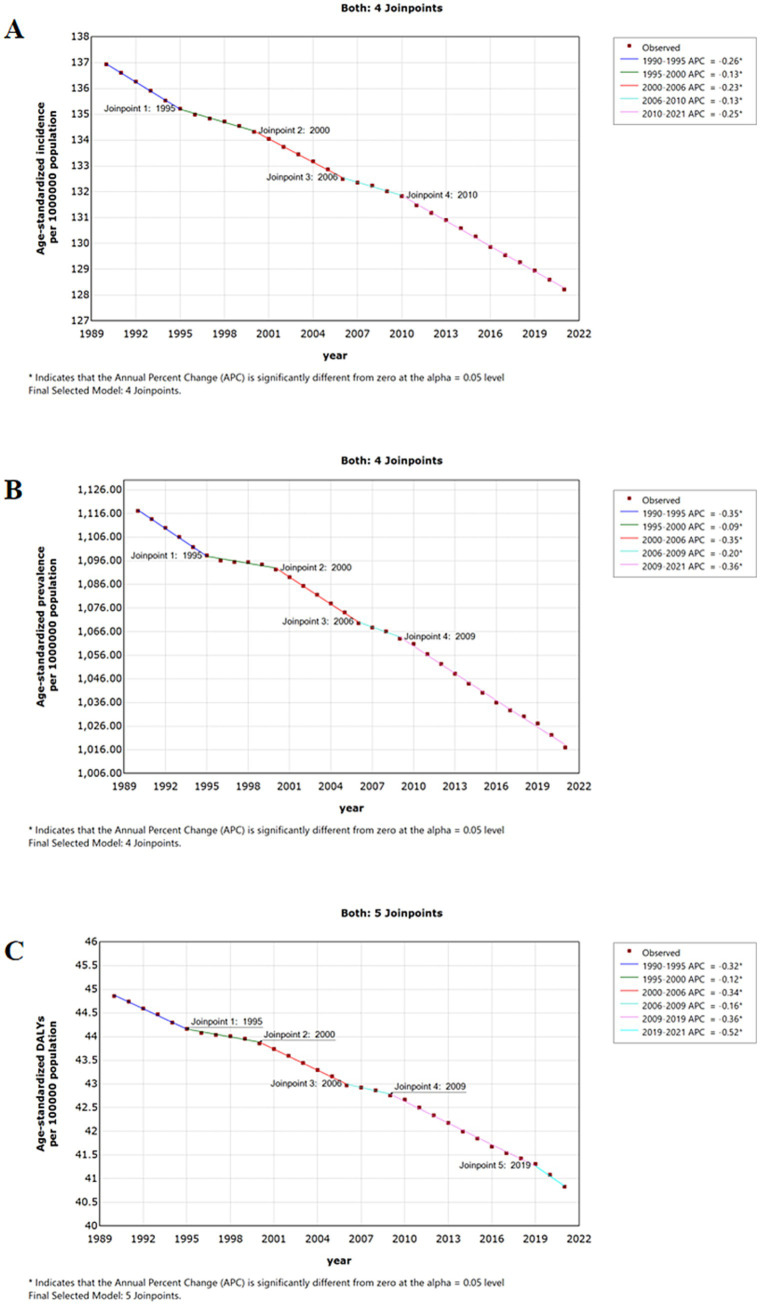
Jointpoint regression analysis of global incidence **(A)**, prevalence **(B)** and DALYs **(C)** of older adult atopic dermatitis from 1990 to 2021. DALYs, disability-adjusted life-years.

Furthermore, the DALYs due to older adult AD in 2021 amounted to 442,714 (95% UI: 236,073 to 743,980), with an age-standardized disability-adjusted life-year rates (ASDRs) of 40.83 per 100,000 population (95% UI: 21.77 to 68.63) and an AAPC of −0.1% (95% CI: −0.2 to −0.1). Notably, incidence, prevalence, and DALY rates exhibited a consistent downward trend with AAPCs < 0% (Figure 1).

### 3.2 Global trends by sex and age

In 2021, globally, the number of older adult AD cases amounted to 4,569,779 in males and 6,439,850 in females, with females being more affected (Table 1). Although the rates of both genders displayed comparable downward trends between 1990 and 2021, females consistently maintained higher rates across all categories. However, sex-specific analysis of older adult AD incidence, prevalence, and DALYs revealed a slight decline in ASRs from 1990 to 2021, with AAPCs approaching 0%.

Incidence of older adult AD increased, with a particularly sharp rise between the ages of 75–79. Similarly, prevalence rates climbed with age, peaking in the 85–89 age bracket. The trend in DALYs followed a similar pattern, reaching its maximum in the 80–84 age group. Across these metrics, females consistently demonstrated slightly higher rates than males in all age categories (Supplementary Figure S1).

### 3.3 Burden of AD based on SDI

**Figure 2:**
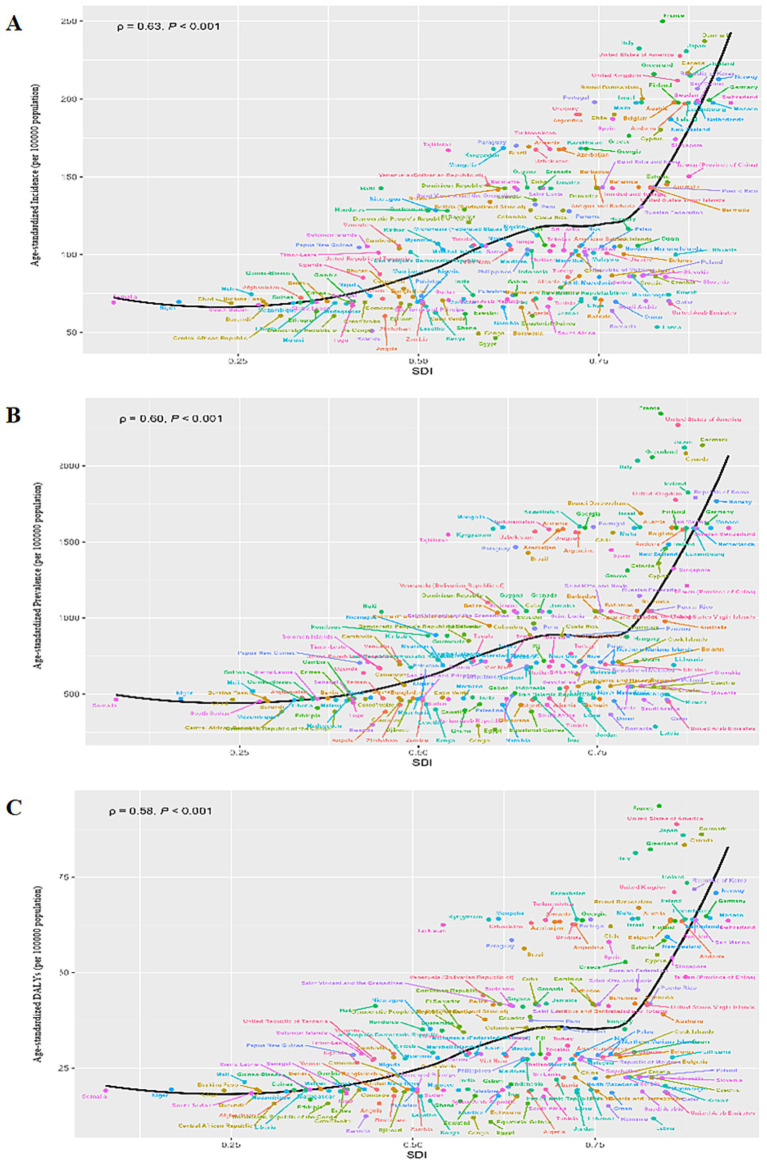
Pearson correlation analysis between the SDI and ASRs of incidence **(A)**, prevalence **(B)**, and DALYs **(C)** for older adult atopic dermatitis across 204 countries and territories levels in 2021. The expected ASRs in 2021 based solely on SDI were represented by the black line. For each country, points depict estimates in 2021. ASRs, age-standardized rates; DALYs, disability-adjusted life-years; SDI, Socio-demographic Index.

Predominantly, the majority of older adult AD cases, including incidence, prevalence, and DALYs, were concentrated in regions with high SDI levels. Upon examining older adult AD indicators across 21 regions, a positive association with SDI became apparent. Specifically, ASRs for incidence, prevalence, and DALYs exhibited robust positive correlations with SDI levels (*R* = 0.673, *p* < 0.001; *R* = 0.659, *p* < 0.001; *R* = 0.660, *p* < 0.001). Notably, eight regions, such as Southern Latin America, Tropical Latin America, and Eastern Europe, surpassed the global averages for prevalence, incidence, and DALYs (Supplementary Figure S2). Consistently, a broader examination encompassing 204 countries and territories reinforced that ASRs for incidence, prevalence, and DALYs positively correlated with SDI levels (*R* = 0.593, *p* < 0.001; *R* = 0.556, *p* < 0.001; *R* = 0.555, *p* < 0.001), respectively (Figure 2).

### 3.4 Region trends

In 2021, the five regions with the highest older adult AD incidence were high-income North America, high-income Asia Pacific, Western Europe, Southern Latin America, and Tropical Latin America (Supplementary Table S1). Among these, high-income North America reported the peak ASIRs for older adult AD, which stood at 226.55 cases per 100,000 population (95% UI: 194.53 to 262.24). Western Europe exhibited the highest number of prevalence, estimated at 2,188,764.91 (95% UI: 1,917,994.06 to 2,479,578.21). In terms of ASPRs, high-income Asia Pacific topped the list with 2027.35 cases per 100,000 population (95% UI: 1,816.82 to 2,246.17). Consistent with the prevalence trends, Western Europe also reported the highest DALYs, totaling 86,960.81 (95% UI: 46,501.71 to 146,619.17). Regarding the ASDRs for older adult AD, high-income North America led with 88.27 cases per 100,000 population (95% UI: 47.32 to 145.35). Across all regions, the AAPCs in ASIRs, ASPRs, and ASDRs for older adult AD remained relatively stable, approaching 0%.

### 3.5 Country trends

**Figure 3:**
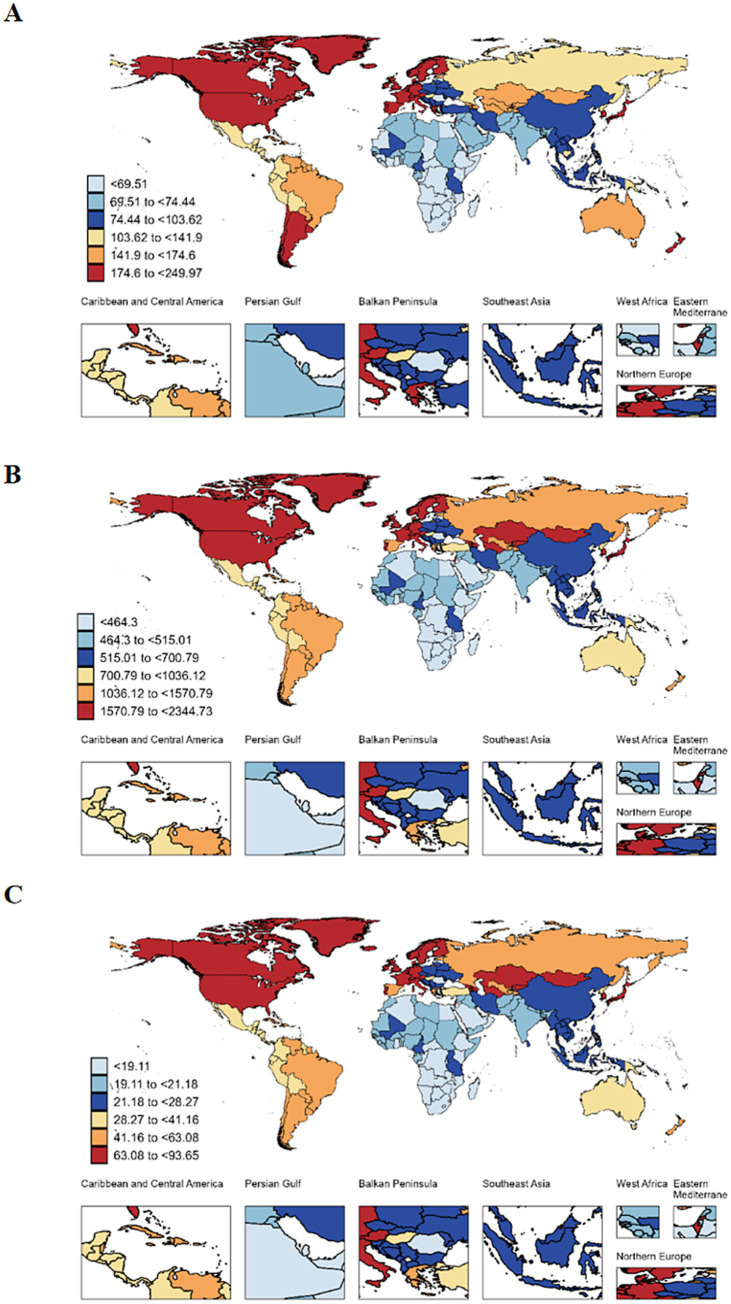
The global incidence **(A)**, prevalence **(B)** and DALYs **(C)** of older adult atopic dermatitis in 204 countries and territories. DALYs, disability-adjusted life-years.

In 2021, three countries reporting the highest incidence of older adult AD were China (270,331.98, 95% UI: 232354.22 to 312366.65), the United States Virgin Islands (179,007.14; 95% UI: 154,561.43 to 206577.38), and India (108,337.77; 95% UI: 92,763.57 to 12,5951.47). Gabon (237.31 per 100,000 population, 95% UI: 177.39 to 304.67), Cabo Verde (249.97 per 100,000 population, 95% UI: 189.38 to 321.84), and Guam (232.53 per 100,000 population, 95% UI: 196.06 to 274.15) exhibited the highest ASIRs for AD (Supplementary Table S2; Figure 3A).

Regarding prevalence, China (1,700,330.02, 95% UI: 1,52,4724.81 to 1,88,8313.9) and the United States Virgin Islands (1,78,5263.55, 95% UI: 1,63,9173.64 to 1942483.31) topped the list in 2021. Cabo Verde (2344.73 per 100,000 population, 95% UI: 1969.09 to 2756.95), Hungary (2270.04 per 100,000 population, 95% UI: 2084.45 to 2469.8), and Gabon (2136.61 per 100,000 population, 95% UI: 1766.51 to 2552.22) had the highest ASPRs for AD (Figure 3B).

The highest DALYs due to older adult AD were recorded in China (70598.84, 95% UI: 37190.42 to 122155.3), the United States Virgin Islands (69810.16, 95% UI: 37347.43 to 114650.94), and Japan (38832.09, 95% UI: 20760.05 to 64776.46). Cabo Verde (93.65 per 100,000 population, 95% UI: 49.64 to 156.92), Hungary (88.86 per 100,000 population, 95% UI: 47.55 to 145.98), and Gabon (86.21 per 100,000 population, 95% UI: 45.18 to 148.21) demonstrated the highest ASDRs for older adult AD (Figure 3C). Notably, the incidence, prevalence, and DALYs rates remained relatively stable, with AAPCs approaching 0% (Supplementary Figure S3).

### 3.6 Decomposition analysis

**Figure 4:**
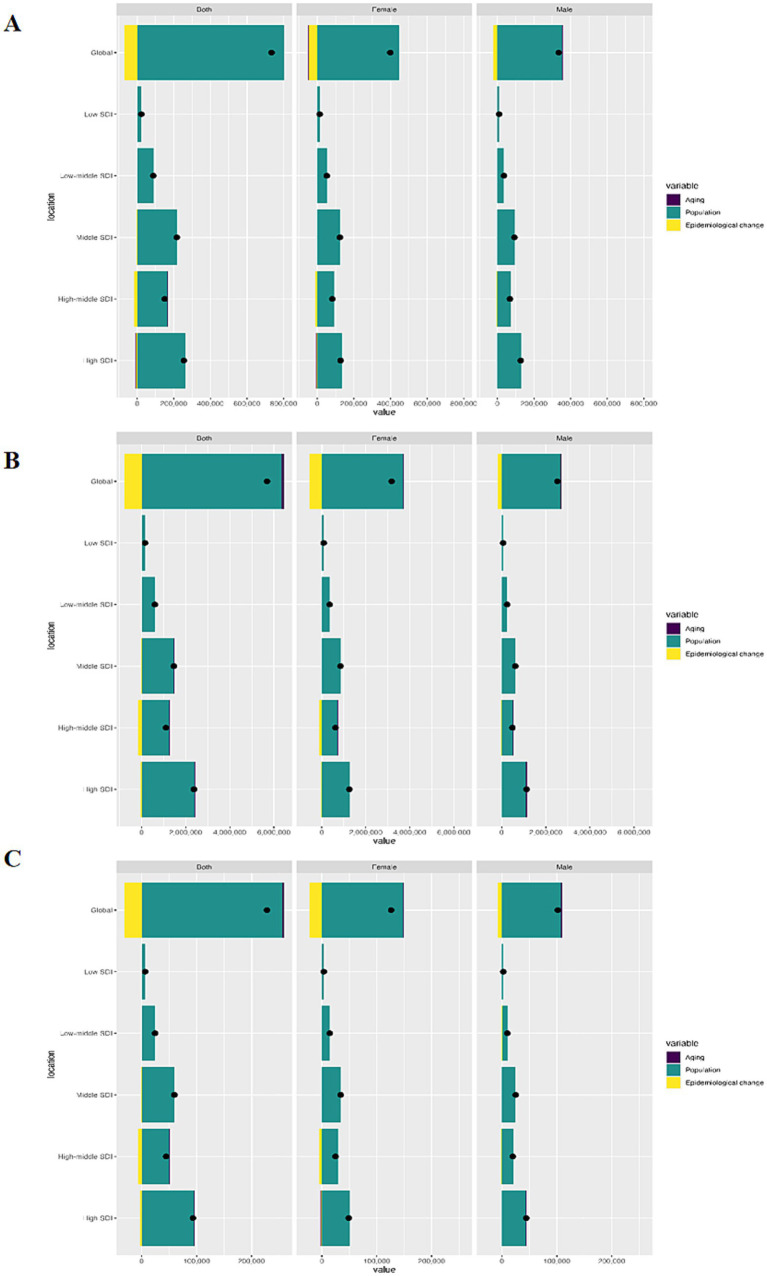
Decomposition analysis of changes in incidence **(A)**, prevalence **(B)** and DALYs **(C)** rates by sex and SDI regions. DALYs, disability-adjusted life-years; SDI, Socio-demographic Index.

Over the past three decades, there had been a remarkable surge in incidence, prevalence, and DALYs number worldwide, with the highest elevation noted in the high SDI quintile, primarily attributed to population growth (Supplementary Table S3). Intriguingly, adverse epidemiological shifts were discernible globally, as well as in the high-middle and high SDI quintiles, with the most significant manifestation observed in the high-middle SDI quintile. When examined by gender, females exhibited a higher burden compared to males across all subgroups (Figure 4).

### 3.7 Cross-country inequality analysis

Considerable absolute and relative disparities related to the SDI levels were noted in the distribution of older adult AD burden across 204 countries (Supplementary Table S4). Notably, DALYs were disproportionately concentrated in countries with high SDI levels. According to the slope index of inequality, the disparity in DALYs between countries with the highest and lowest SDI was 45.46 (95%CI: 38.14 to 52.77) in 1990 and 35.6 (95%CI: 28.98 to 42.22) in 2021. Additionally, the concentration index underwent a significant decline, from 0.29 (95%CI: 0.26 to 0.31) in 1990 to 0.26 (95%CI: 0.23 to 0.3) in 2021 (Supplementary Figure S4).

### 3.8 Frontier analysis

In our extensive frontier analysis, spanning from 1990 to 2021 across 204 countries and territories, and utilizing SDI and ASDRs for older adult AD, subtle trends emerged (Supplementary Table S5). As SDI increased from 0.0 to 1.0, a gradual decline in older adult AD’s ASDRs was observed, evident in the shifting density from darker to lighter shades over the years, signifying an overall decrease in DALYs. Examining the 2021 frontier analysis results, clear distinctions among nations and territories were depicted visually. Notably, 10 countries, including France, high-income North America, and Denmark, exhibited notably higher rates, placing them substantially away from the frontier. Conversely, Somalia, Rwanda, the Central African Republic, and Niger were positioned nearer to the frontier, indicating favorable outcomes relative to their SDI levels (Supplementary Figure S5).

### 3.9 Predictive analysis to 2050

The estimated number of cases and ASRs of incidence, prevalence, and DALYs for older adult AD up to 2050 were depicted in Supplementary Figure S6. On a global scale, an increase in the number of cases and ASRs for both incidence and prevalence was anticipated. In contrast, for DALYs, while the number of cases was projected to rise, the annual ASRs was forecasted to decline until 2050. The comprehensive figures for the number of cases and ASRs for incidence, prevalence, and DALYs were provided in Supplementary Table S6.

## 4 Discussion

This research presented the latest data on the burden of older adult AD at the global, regional, and national levels, spanning the period from 1990 to 2021. Although there were variations in incidence, prevalence and DALYs of older adult AD across countries, the burden older adult AD increased overall from 1990 to 2021. SDI relative and cross-country inequality analysis revealed high SDI countries shouldering a disproportionate older adult AD burden. Decomposition analysis found population growth as a major factor driving the changes in older adult AD burden. Of note, although the ASRs of DALYs were predicted to slightly decrease annually from 2022 to 2050, the absolute numbers of incidence, prevalence and DALYs were predicted to keep increasing, suggesting a huge challenge in the control and management of older adult AD remained in the next decades.

Prior studies have indicated that the ASPRs and ASIRs remained relatively stable between 1990 and 2019 (14), while our study reported a slight decrease. It may be attributed to advancements in treatment modalities, enhanced prevention and control measures, environmental and lifestyle changes, genetic factors, birth cohort effects, as well as shifts in diagnostic criteria and research methodologies (15). Additionally, the rise in overall incidence, prevalence, and DALYs was primarily due to population growth rather than an actual expansion in the proportion of affected individuals, suggesting that improvements in healthcare and public health initiatives may have contributed to mitigating the burden of AD among the older adult.

Older adult AD incidence peaked in the 75–79 age group. Notably, many trials have restricted the participation of older adults by imposing upper age limits (ranging from 40 to 70 years) or by establishing eligibility criteria that disproportionately exclude this population, thereby hindering the assessment of the efficacy and safety of novel therapies in the older adult, which ultimately limits their utilization in this demographic.

Our study indicated that the incidence of AD was higher in older adult females than in older adult males. However, existing research on the incidence of AD in males and females remains controversial (16, 17). Significant changes occur in hormone homeostasis and the immune system of older adult females, making them more susceptible to skin barrier dysfunction and immune disturbances, which may account for the higher incidence of AD in this demographic (18, 19). Additionally, this disparity may also be attributed to variations in the surveyed regions and populations.

The ASIRs, ASPRs, and ASDRs of AD exhibited regional and national differences, remained relatively stable across 21 regions and 204 countries from 1990 to 2021. As previously reported in international studies, higher levels of the SDI tend to be associated with higher prevalence rates of AD (20), which increases with rising SDI. The high-income areas such as North America, Western Europe and high-income Asia Pacific exhibited higher incidence and prevalence rates, potentially linked to the rapid pace of urbanization and economic development, where older adult individuals may experience lifestyle alterations and exposure to environmental pollutants, both known risk factors for AD (21, 22). Furthermore, countries with higher SDI typically possess better healthcare systems and greater awareness of older adult AD, leading to improved diagnosis and reporting of the disease (23, 24). This disparity underscores the multifaceted determinants of healthcare outcomes, indicating that while SDI is a significant factor, other underlying elements (environmental, genetic, or healthcare system complexities) also play crucial roles in shaping AD trends. Heterogeneity in the burden of older adult AD across SDI regions and countries underscores the importance of tailored interventions and public health strategies.

We also presented the global burden of older adult AD until 2050. Our findings indicated that the incidence and prevalence of older adult AD were anticipated to rise by 2050, posing an even greater societal burden globally. Consequently, healthcare systems will continue to bear a significant burden due to this disease in the future. These trends underscore the urgency for the formulation of nuanced health policies that focus on reducing the incidence and improving the long-term prognosis for older adult patients with AD.

This study also has several limitations. Firstly, there was considerable variation in the quality and accessibility of healthcare data across different regions, which may introduce biases or inaccuracies in the results. Secondly, similar to all analyses utilizing GBD data, this study was constrained by limitations in case ascertainment. The identification of older adult AD cases in the GBD study hinges on a range of data sources and coding systems, potentially leading to either underestimation or overestimation of older adult AD prevalence and burden in specific regions or populations. Additionally, this study lacked a classification analysis of older adult AD severity.

## 5 Conclusion

Globally, AD represented an underestimated public health concern primarily affecting individuals aged 60 and older, with variations observed across genders, age groups, regions, and nations. Between 1990 and 2021, the burden of older adult AD remained high varied by gender, age and countries. Those within the 80–89 age bracket, particularly females, bore a heavier burden of AD and thus warrant greater attention. Consequently, targeted strategies for the effective prevention and management of AD in the older adult are needed to address the impending challenge.

## Data Availability

The original contributions presented in the study are included in the article/Supplementary material, further inquiries can be directed to the corresponding authors.

## Footnotes

^1^ http://ghdx.healthdata.org/gbd-results-tool
