# Supplementary material for: Global, regional, and national burden of older adult atopic dermatitis in 204 countries and territories worldwide
Source: Front Public Health. 2025 Apr 2;13:1569119. doi: 10.3389/fpubh.2025.1569119 (PMC11999981; doi:10.3389/fpubh.2025.1569119)
Supplement: Supplementary file 1 [file Supplementary_file_1.docx]

Supplementary Material

# Supplementary Figures and Tables

## Supplementary Figures


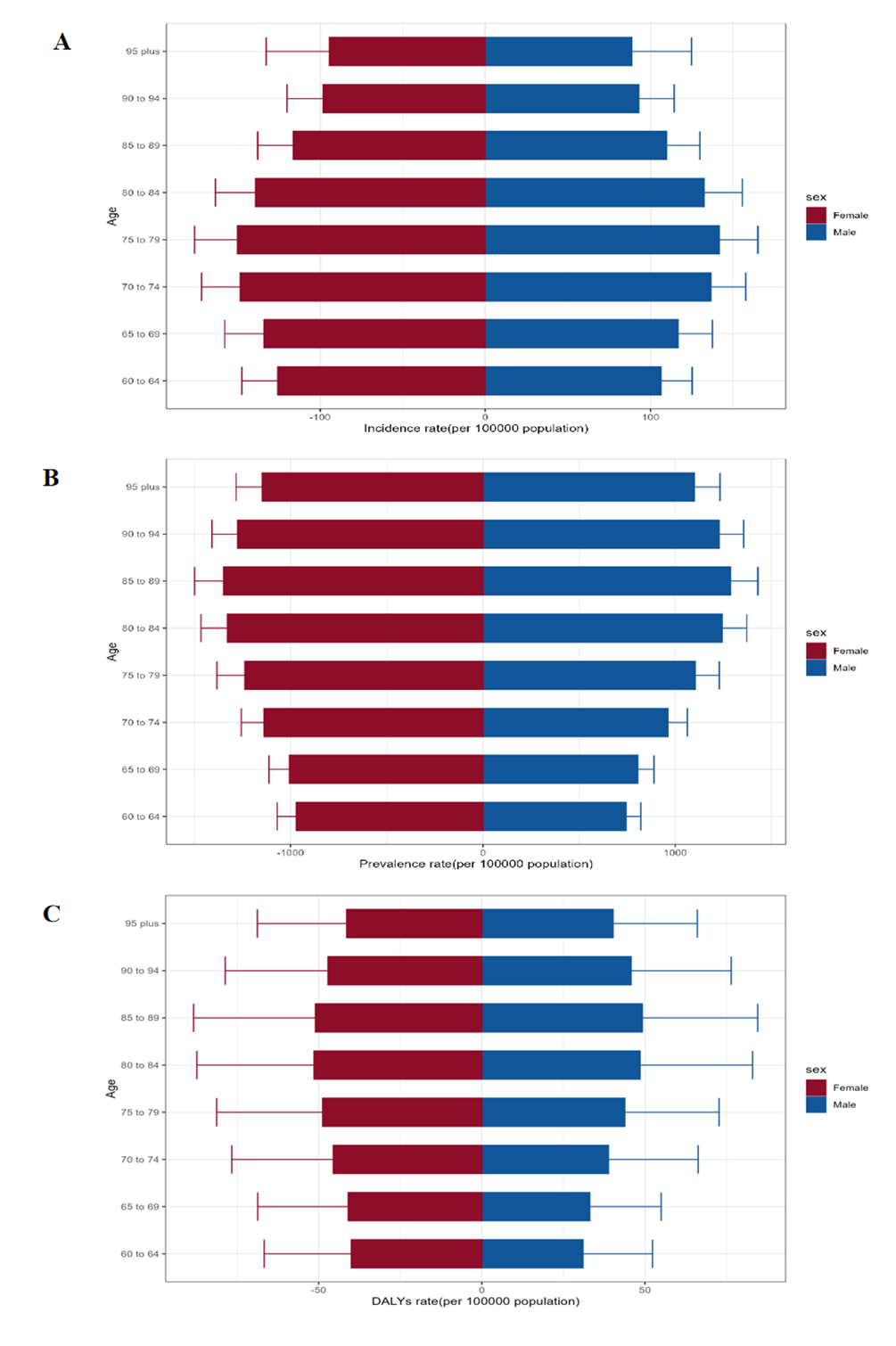


# Supplement Figure 1. The trends of incidence (A), prevalence (B), and DALYs (C) for elderly atopic dermatitis across different genders, female and male, by age groups ranging from 60 years to 95+ years. DALYs, disability-adjusted life-years.


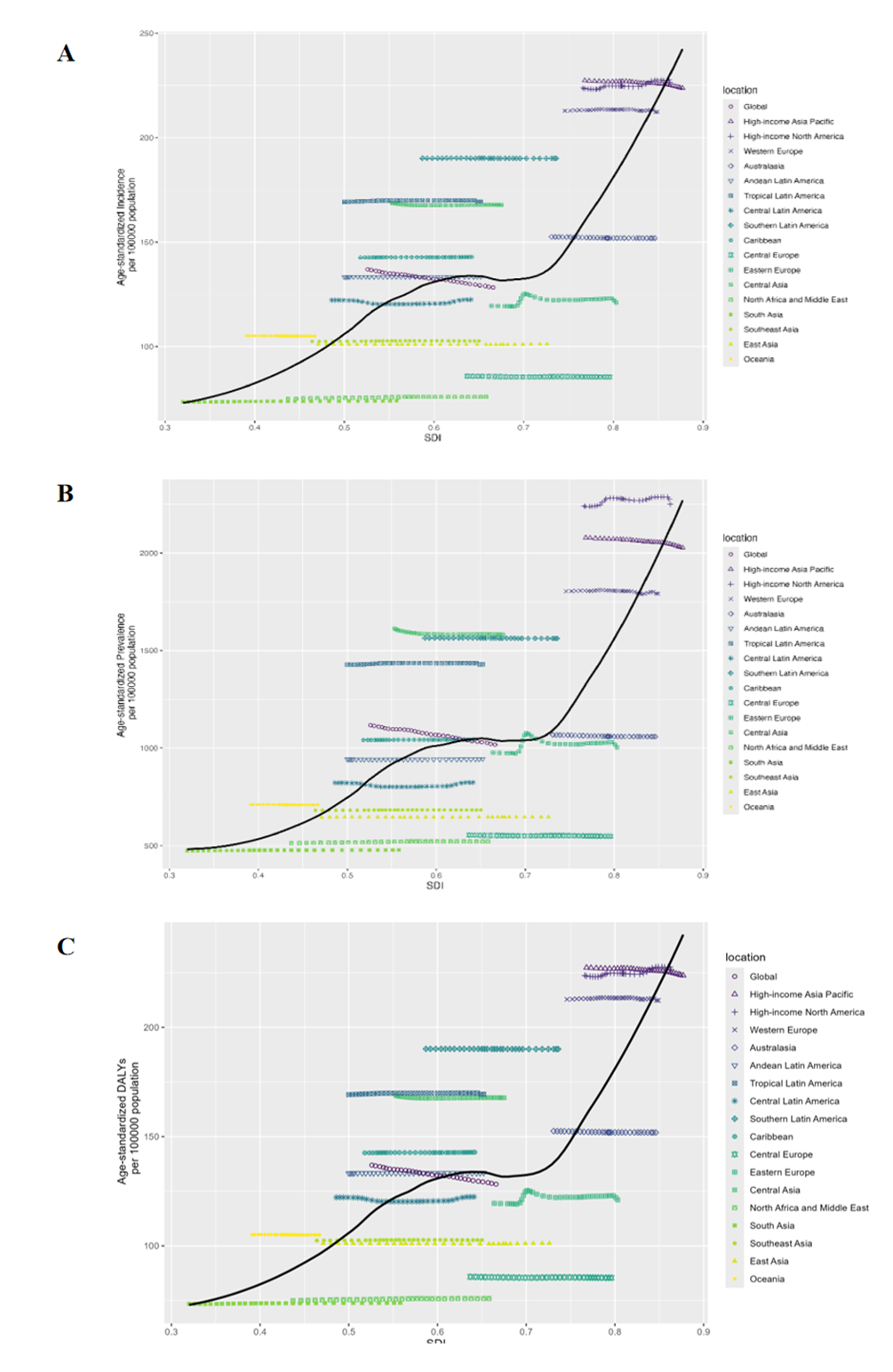


**Supplement Figure 2. The ASRs of incidence (A), prevalence (B), and DALYs (C) for elderly atopic dermatitis globally and for 21 GBD regions by SDI from 1990 to 2021. The expected ASRs in 2021 based solely on SDI were represented by the black line. For each region, points from left to right depict estimates from each year from 1990 to 2021. ASRs, age-standardized rates; DALYs, disability-adjusted life-years; GBD, Global Burden of Diseases; SDI, socio-demographic index.**


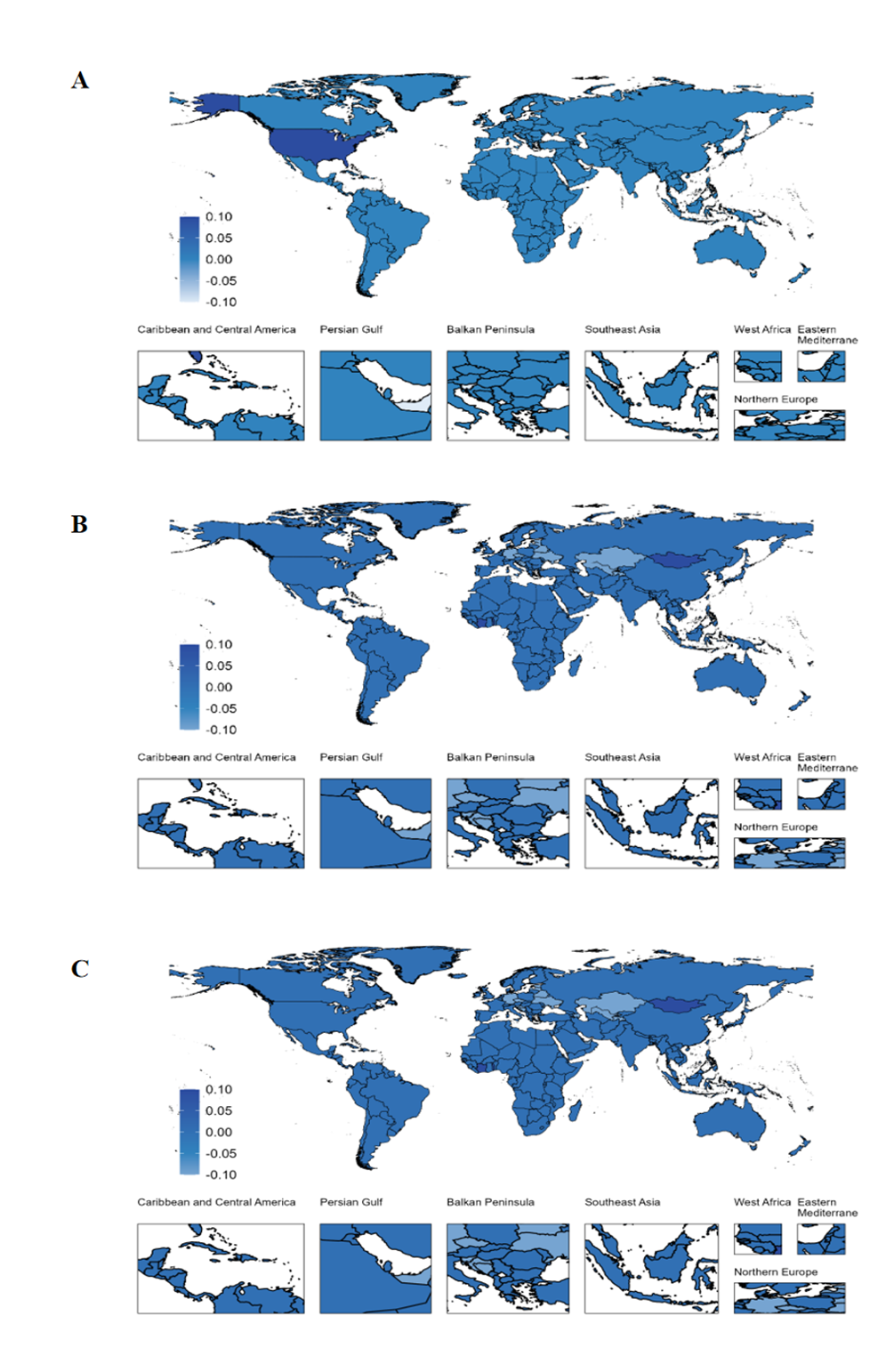


**Supplement Figure 3. Global maps of AAPCs of incidence (A), prevalence (B), and DALYs (C) for elderly atopic dermatitis from 1990 to 2021. AAPCs, average annual percent changes; DALYs, disability-adjusted life-years.**


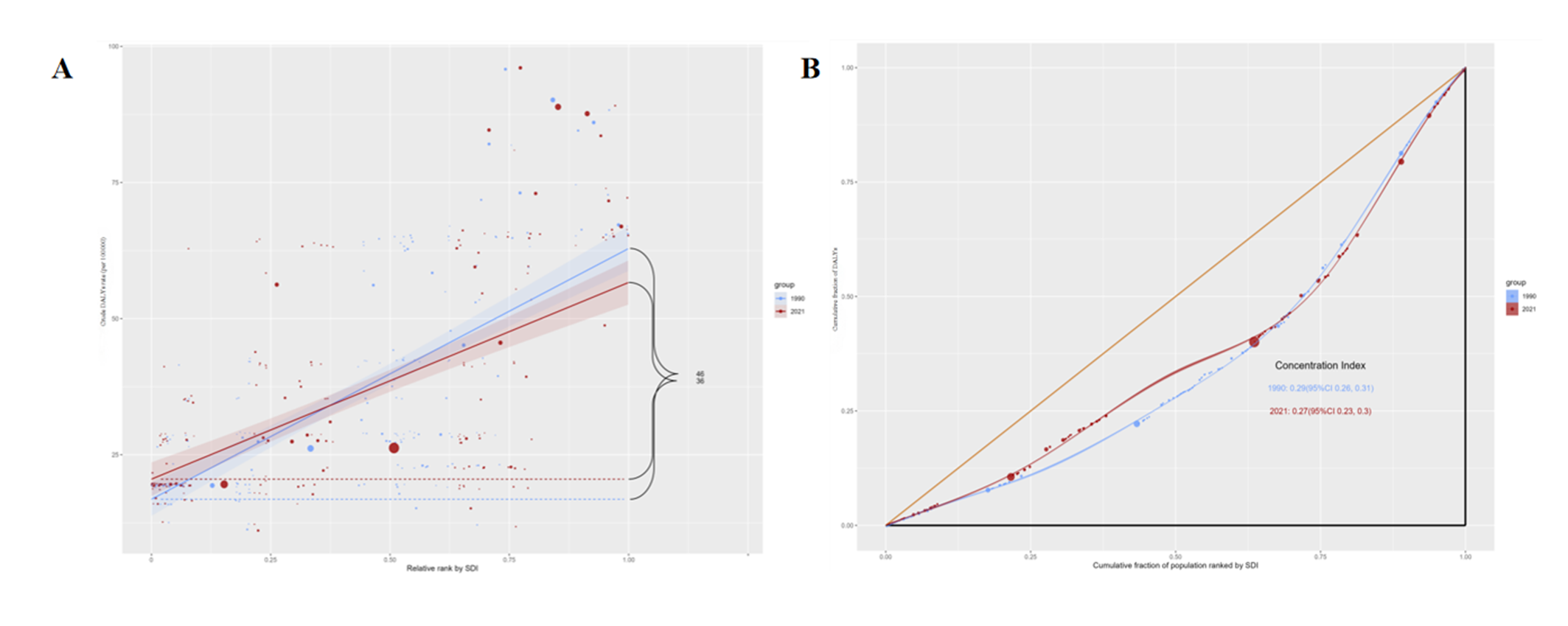


**Supplement Figure 4. SDI-related health inequality regression (A) and concentration (B) curves for the DALYs of elderly atopic dermatitis worldwide, 1990 and 2021. DALYs, disability-adjusted life-years; SDI, socio-demographic index.**


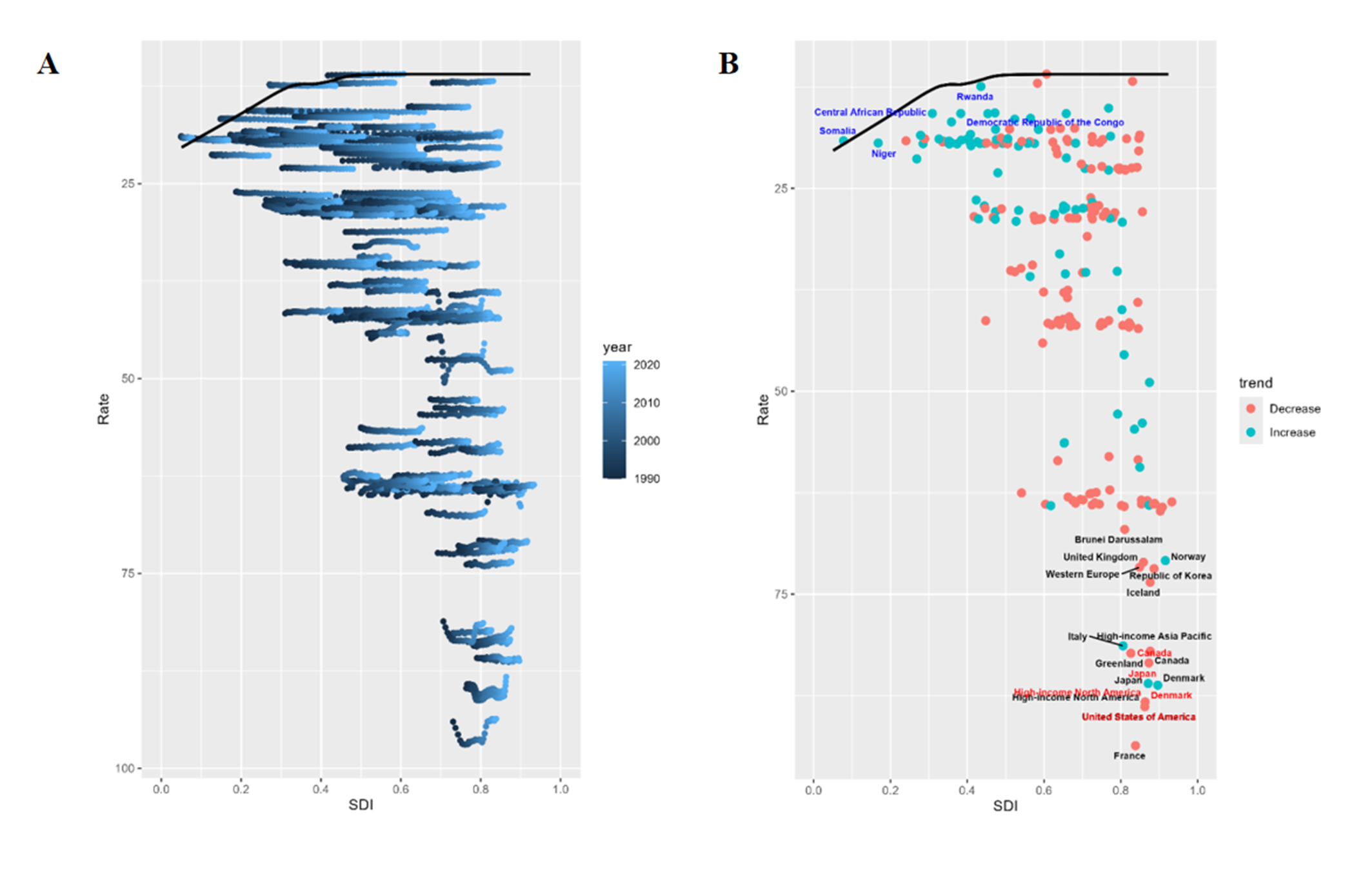
**Supplement Figure 5. Frontier analysis, represented by the solid black lines, explores the relationship between SDI and ASRs for DALYs (A, B) in the context of elderly atopic dermatitis. The color gradient in graphs A illustrates the progression of years, ranging from light shades representing 1990 to the darkest shades denoting 2021. In graphs B, each dot signifies a specific country for the year 2021, with the top 15 countries displaying the most significant deviation from the frontier labeled in black. Countries with low SDI and minimal deviation from the frontier are highlighted in blue, while those with high SDI and notable deviation for their developmental level are emphasized in red. The direction of change from 1990 to 2021 in ASRs is indicated by the color of the dots: decrease dots represents a decrease, while increase dots signifies an increase. ASRs, age-standardized rates; DALYs, disability-adjusted life-years; SDI, socio-demographic index.**


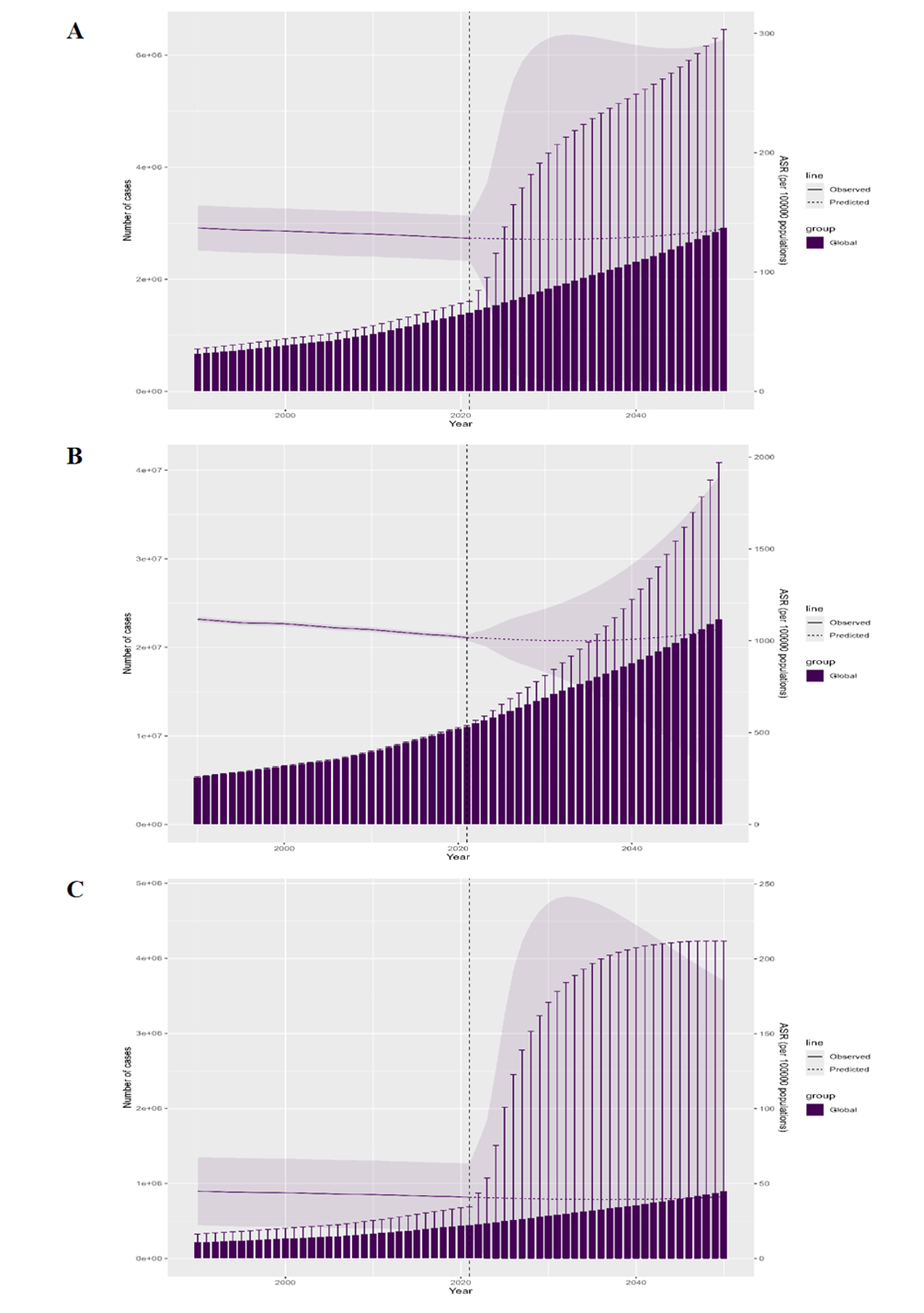


**Supplement Figure 6. Predictive analysis of incidence (A), prevalence (B) and DALYs (C) for elderly atopic dermatitis in global to 2050 year. The bar graphs represent the quantity, while the line graphs represent ASRs. ASRs, age-standardized rates; DALYs, disability-adjusted life-years.**

## Supplementary Tables

**Supplement Table 1. Numbers and ASRs per 100 000 cases of incidence, prevalence and DALYs of elderly** **atopic dermatitis in 1990 and 2021, along with the AAPC in ASRs per 100 000 cases from 1990 to 2021, categorized by 21 regions.**

| **Regions** | **Incidence (95% UI)** | | | | | | **Prevalence (95% UI)** | | | | | | **DALYs (Disability-Adjusted Life Years) (95% UI)** | | | | | |
| --- | --- | --- | --- | --- | --- | --- | --- | --- | --- | --- | --- | --- | --- | --- | --- | --- | --- | --- |
|  | **Absolute number** | | **ASR, per 100 000 population** | | | | **Absolute number** | | **ASR, per 100 000 population** | | | | **Absolute number** | | **ASR, per 100 000 population** | | | |
|  | **1990** | **2021** | **1990** | **2021** | **AAPC** | ***P*** | **1990** | **2021** | **1990** | **2021** | **AAPC** | ***P*** | **1990** | **2021** | **1990** | **2021** | **AAPC** | ***P*** |
| Andean Latin America | 3171.51 (2558.51 to 3825.45) | 9620.51 (7760.87 to 11607.13) | 133.2 (107.35 to 160.83) | 133.38 (107.56 to 160.97) | 0 | 0.656 | 22317.73 (19638.32 to 25270.55) | 67946.41 (59723.11 to 77001.44) | 942.14 (828.21 to 1067.94) | 943.93 (829.36 to 1070.15) | 0 | 0 | 901.62 (479.77 to 1520.14) | 2726.54 (1458.1 to 4554.68) | 37.97 (20.19 to 64.06) | 37.85 (20.23 to 63.23) | 0 | 0.714 |
| Central Latin America | 11786.17 (10059.83 to 13701.77) | 37885.53 (32304.14 to 44121.93) | 122.17 (104.16 to 142.1) | 122.43 (104.4 to 142.56) | 0 | 0.674 | 78703.7 (71039.93 to 87159.13) | 254061.02 (228899.08 to 281619.3) | 822.17 (741.77 to 910.51) | 823.37 (741.74 to 912.45) | 0 | 0.757 | 3177.81 (1695.53 to 5357.2) | 10228.43 (5478.99 to 17295.91) | 33.06 (17.63 to 55.74) | 33.09 (17.72 to 55.97) | 0 | 0.686 |
| Oceania | 344.67 (268.91 to 429.79) | 848.13 (661.79 to 1057.36) | 105.15 (82.18 to 131.09) | 105.03 (82.08 to 130.92) | 0 | 0.002 | 2283.86 (1957.93 to 2641.82) | 5636.69 (4833.64 to 6521.81) | 709.9 (608.36 to 821.8) | 708.82 (607.54 to 820.68) | 0 | 0 | 93.09 (48.78 to 161.07) | 229.12 (123.76 to 395.47) | 28.68 (15.01 to 49.64) | 28.59 (15.36 to 49.37) | 0 | 0 |
| Southeast Asia | 29942.6 (24741.28 to 35588.63) | 82058.7 (67660.42 to 97604.81) | 102.42 (84.64 to 121.72) | 102.66 (84.65 to 122.09) | 0 | 0.669 | 196991.79 (174290.17 to 222058.06) | 540600.89 (478713.85 to 608075.42) | 680.55 (601.91 to 767.23) | 682.47 (603.94 to 767.87) | 0 | 0 | 8025.17 (4326.06 to 13529.9) | 22000.66 (11855.35 to 37731.22) | 27.56 (14.78 to 46.56) | 27.61 (14.84 to 47.4) | 0 | 0 |
| East Asia | 105872.43 (90555.66 to 122182.76) | 283715.29 (243386.36 to 328731.74) | 101.06 (86.3 to 116.72) | 101.14 (86.7 to 117.26) | 0 | 0.627 | 666395.21 (598296.4 to 739406.79) | 1803175.15 (1616429.8 to 2006763.26) | 645.33 (579.77 to 715.76) | 647 (579.99 to 719.93) | 0 | 0 | 27782.08 (14689.56 to 47948.35) | 74757.04 (39494.26 to 129014.13) | 26.75 (14.13 to 46.33) | 26.77 (14.15 to 46.3) | 0 | 0.704 |
| Central Europe | 16861.77 (13754.15 to 20197.38) | 25587.04 (21009.44 to 30524.66) | 85.82 (70.04 to 102.79) | 85.38 (70.07 to 101.86) | 0 | 0.426 | 108627.55 (95441.54 to 122948.17) | 164294.04 (144763.7 to 185743.13) | 554.11 (486.97 to 627.17) | 548.29 (483.06 to 619.75) | 0 | 0 | 4456.86 (2366.84 to 7767.68) | 6734.03 (3574.47 to 11668.31) | 22.67 (12.03 to 39.54) | 22.5 (11.95 to 39.01) | 0 | 0.001 |
| North Africa and Middle East | 14693.07 (11513.15 to 18126) | 39993.05 (31316.73 to 49533.82) | 74.9 (58.75 to 92.45) | 75.93 (59.54 to 94.04) | 0 | 0 | 99594.05 (85683.1 to 114956.49) | 272956.11 (234593.43 to 315239.82) | 512.17 (440.33 to 591.46) | 522.04 (448.86 to 603.11) | 0.1 | 0 | 4115.96 (2190.47 to 7133.26) | 11165.53 (6025.89 to 19396.74) | 21.05 (11.17 to 36.58) | 21.27 (11.44 to 37) | 0 | 0 |
| Eastern Sub-Saharan Africa | 6035.45 (4932.69 to 7241.68) | 13200.46 (10804.15 to 15800.06) | 70.21 (57.27 to 84.39) | 70.22 (57.42 to 84.09) | 0 (0 to 0.1) | 0 | 40883.36 (36026.48 to 46099.74) | 89217.14 (78482.16 to 100590.42) | 481.31 (423.9 to 543.08) | 479.57 (421.94 to 540.69) | 0 | 0.961 | 1685.88 (909.59 to 2915.69) | 3692.51 (1967.4 to 6361.02) | 19.74 (10.6 to 34.31) | 19.76 (10.48 to 34.15) | 0 | 0.016 |
| South Asia | 47910.85 (40901.17 to 55953.17) | 132864.15 (113405.38 to 155143.11) | 73.45 (62.61 to 85.71) | 73.92 (63.03 to 86.3) | 0 | 0 | 305580.76 (275143.9 to 338907.3) | 851834.63 (766180.23 to 945210.84) | 473.8 (426.51 to 525.53) | 477.4 (429.41 to 529.7) | 0 | 0 | 12500.35 (6570.69 to 21723.8) | 34880 (18276.36 to 61028.74) | 19.26 (10.1 to 33.62) | 19.47 (10.18 to 34.16) | 0 | 0 |
| Western Sub-Saharan Africa | 7211.5 (5974.4 to 8586.77) | 15357.95 (12680.65 to 18363.84) | 69.9 (57.92 to 83.2) | 70.11 (57.91 to 83.8) | 0 | 0.743 | 48047.38 (42564.47 to 53977.82) | 102379.08 (90417.81 to 115018.63) | 469.96 (416.49 to 527.76) | 471.51 (416.47 to 529.63) | 0 | 0 | 1986.01 (1053.5 to 3433.57) | 4247.15 (2242.93 to 7346.34) | 19.35 (10.23 to 33.55) | 19.48 (10.25 to 33.78) | 0 | 0 |
| Southern Sub-Saharan Africa | 2135.14 (1818.28 to 2507.69) | 4648.36 (3964.99 to 5440.72) | 66.38 (56.47 to 77.98) | 66.61 (56.73 to 77.99) | 0 | 0 | 13679.89 (12259.97 to 15240.46) | 29726.92 (26715.38 to 33097.08) | 428.72 (384.09 to 477.56) | 430.23 (386.42 to 478.92) | 0 | 0 | 567.08 (300.49 to 990.52) | 1222.78 (659.01 to 2130.11) | 17.71 (9.37 to 30.98) | 17.63 (9.46 to 30.76) | 0 | 0 |
| Central Sub-Saharan Africa | 1539.38 (1209.58 to 1890.4) | 3580.39 (2814.91 to 4392.72) | 60.14 (47.3 to 74.04) | 60.4 (47.5 to 74.33) | 0 | 0 | 9627.96 (8272.17 to 11126.49) | 22427.57 (19274.53 to 25918.99) | 381.21 (327.24 to 440.81) | 382.7 (328.4 to 442.6) | 0 | 0 | 396.64 (207.8 to 690.45) | 926.97 (486.1 to 1631.21) | 15.59 (8.14 to 27.24) | 15.72 (8.22 to 27.77) | 0 | 0.023 |
| High-income North America | 104024.21 (89293.75 to 120529.52) | 199824.2 (171605.75 to 231295.31) | 223.74 (191.92 to 259.42) | 226.55 (194.53 to 262.24) | 0 (0 to 0.1) | 0 | 1040762.64 (954641.46 to 1131318.13) | 1986458.93 (1821199.82 to 2163430.94) | 2239.31 (2054.34 to 2433.78) | (328.4 to 442.6) | 0 | 0.017 | 41405.42 (22159.81 to 68240.35) | 77852.33 (41738.51 to 128186.45) | 89.22 (47.78 to 147.06) | 88.27 (47.32 to 145.35) | 0 (-0.1 to 0) | 0.011 |
| High-income Asia Pacific | 58304.08 (48810.71 to 69012.39) | 128564.33 (106272.91 to 152840.2) | 227.33 (190.17 to 269.25) | 223.76 (184.78 to 266.27) | -0.1 (-0.1 to -0.1) | 0 | 527239.03 (475250.09 to 582339.17) | 1207324.74 (1082984.96 to 1336222.33) | 2078.51 (1873.82 to 2295.2) | 2027.35 (1816.82 to 2246.17) | -0.1 | 0 | 21307.95 (11372.54 to 35457.69) | 48451.99 (25896.04 to 81020.51) | 83.79 (44.73 to 139.52) | 82.01 (43.93 to 137.14) | -0.1 | 0 |
| Western Europe | 163341.36 (132109.55 to 198490.71) | 250192.44 (200948.52 to 304239.33) | 212.86 (172.06 to 258.58) | 212.34 (170.85 to 257.85) | 0 | 0.002 | 1395293.47 (1227463.33 to 1576047.72) | 2188764.91 (1917994.06 to 2479578.21) | 1804.04 (1586.95 to 2037.13) | 1792.67 (1572.15 to 2028.86) | 0 | 0.048 | 55645.78 (29798.32 to 93215.58) | 86960.81 (46501.71 to 146619.17) | 72.04 (38.63 to 120.63) | 71.68 (38.41 to 120.8) | 0 | 0.037 |
| Central Asia | 9619.31 (7285.67 to 12223.08) | 16711.71 (12640.56 to 21249.6) | 168.79 (127.89 to 214.66) | 167.79 (127.14 to 213.26) | 0 | 0 | 91444.4 (78941.01 to 105323.07) | 156283.57 (135035.8 to 180048.26) | 1611.66 (1390.43 to 1858.93) | 1581.99 (1366.12 to 1824.8) | -0.1 | 0 | 3685.45 (1981.33 to 6157.09) | 6300.48 (3374.15 to 10715.62) | 64.76 (34.72 to 107.85) | 63.45 (33.94 to 107.52) | -0.1 | 0 |
| Southern Latin America | 11335.18 (8748.01 to 14222.03) | 21363.45 (16477.68 to 26813.55) | 190.15 (146.64 to 238.71) | 190.07 (146.55 to 238.58) | 0 | 0 | 92185.73 (79248.55 to 106513.4) | 176152.38 (151435.05 to 203480.77) | 1564.23 (1344.69 to 1807.02) | 1562.65 (1343.52 to 1804.91) | 0 | 0 | 3707.52 (1952.34 to 6220.09) | 7034.16 (3749.15 to 12042.82) | 62.79 (33.05 to 105.36) | 62.46 (33.29 to 106.92) | 0 | 0.176 |
| Tropical Latin America | 18385.19 (16003.68 to 21087.97) | 54798.81 (47659.63 to 62867.65) | 169.21 (147.09 to 194.1) | 169.43 (147.33 to 194.29) | 0 | 0.74 | 152760.12 (139125.56 to 167652.9) | 460190.42 (419227.74 to 504914.41) | 1428.44 (1300.98 to 1566.71) | 1429.46 (1302.2 to 1567.95) | 0 | 0.93 | 6055.64 (3249.41 to 9952.39) | 18182.26 (9679.26 to 30067.59) | 56.33 (30.2 to 92.68) | 56.38 (30.01 to 93.24) | 0 | 0.969 |
| Australasia | 4764.05 (3818.04 to 5837.49) | 10607.83 (8426.57 to 13011.14) | 152.53 (122.1 to 187.04) | 151.86 (120.57 to 186.27) | 0 (-0.1 to 0) | 0.021 | 33128.47 (28198.24 to 38436.9) | 74407.32 (63273.35 to 86543.2) | 1067.89 (909.22 to 1238.7) | 1058.88 (900.47 to 1231.13) | 0 | 0 | 1322.16 (708.59 to 2279.55) | 2962.73 (1564.16 to 5027.01) | 42.59 (22.81 to 73.51) | 42.28 (22.34 to 71.7) | 0 | 0.007 |
| Caribbean | 4602.7 (3581.82 to 5712.56) | 9576.05 (7446.84 to 11905.09) | 142.55 (110.85 to 177.09) | 142.86 (111.15 to 177.53) | 0 | 0 | 33404.15 (28603.95 to 38659.9) | 70029.18 (59946.97 to 81082.28) | 1041.28 (891.62 to 1205.03) | 1043.97 (893.88 to 1208.58) | 0 | 0 | 1352.59 (727.72 to 2283.89) | 2802.26 (1493.99 to 4736.2) | 42.1 (22.65 to 71.12) | 41.79 (22.29 to 70.66) | 0 | 0 |
| Eastern Europe | 44045.15 (38543.65 to 50108.88) | 58504.7 (51319.39 to 66533.34) | 119.45 (104.5 to 135.89) | 121.04 (106.2 to 137.62) | 0 (0 to 0.1) | 0 | 360167.24 (330987.48 to 391927.13) | 485762.55 (447144.79 to 528071.07) | 975.88 (896.47 to 1061.99) | 1003.77 (923.75 to 1091.45) | 0.1 | 0 | 14425.69 (7717.4 to 23953.55) | 19355.96 (10423.93 to 31968.19) | 38.98 (20.85 to 64.7) | 39.96 (21.49 to 66.05) | 0.1 (0 to 0.1) | 0 |

**Abbreviations:** AAPC, average annual percent change; ASRs, age-standardized rates; DALYs, disability-adjusted life-years.

**Supplement Table 2. Numbers and ASRs per 100000 cases of incidence, prevalence and DALYs of elderly** **atopic dermatitis in 1990 and 2021, categorized by 204 countries and territories.**

| **Countries** | **Incidence (95% UI)** | | | | **Prevalence (95% UI)** | | | | **DALYs (Disability-Adjusted Life Years) (95% UI)** | | | | |
| --- | --- | --- | --- | --- | --- | --- | --- | --- | --- | --- | --- | --- | --- |
|  | **Absolute number** | | **ASR, per 100 000 population** | | **Absolute number** | | **ASR, per 100 000 population** | | **Absolute number** | | **ASR, per 100 000 population** | | |
|  | **1990** | **2021** | **1990** | **2021** | **1990** | **2021** | **1990** | **2021** | **1990** | **2021** | **1990** | **2021** | |
| Afghanistan | 605.64 (468.58 to 757.58) | 584.14 (452.07 to 730.69) | 71.02 (55.03 to 88.85) | 101.69 (80.03 to 125.84) | 3962.31 (3391.98 to 4604.35) | 3846.29 (3290.21 to 4469.43) | 1595.27 (1374.02 to 1843.57) | 677.28 (580.18 to 783.48) | 163.08 (84.28 to 293.26) | 154.9 (79.83 to 280.05) | 27.54 (14.16 to 48.45) | 27.53 (14.28 to 47.26) | |
| Albania | 207.98 (163.54 to 255.36) | 515.15 (405.69 to 631.98) | 85.66 (67.47 to 105.15) | 167.99 (127.2 to 213.52) | 1315.61 (1131.71 to 1518.1) | 3258 (2801.25 to 3759.01) | 676.4 (607.47 to 751.41) | 1585.51 (1365.89 to 1831.28) | 54.59 (28.42 to 97.5) | 135.52 (70.49 to 239.65) | 63.92 (33.12 to 109.52) | 63.38 (33.22 to 107.97) | |
| Algeria | 1067.68 (801.48 to 1377.39) | 3038.05 (2278.55 to 3923.42) | 70.97 (53.26 to 91.59) | 102.4 (87.45 to 118.37) | 6917.68 (5571.66 to 8446.2) | 19742.2 (15902.82 to 24081.1) | 630.62 (567.95 to 698.86) | 677.15 (609.99 to 749.39) | 287 (144.98 to 506.13) | 812.73 (415.49 to 1434.83) | 27.39 (14.44 to 46.64) | 27.42 (14.68 to 46.36) | |
| American Samoa | 2.64 (2.06 to 3.29) | 6.02 (4.7 to 7.5) | 105.55 (82.52 to 131.59) | 99.8 (85.73 to 115.42) | 17.58 (15.07 to 20.34) | 40.26 (34.51 to 46.57) | 713.13 (610.95 to 825.34) | 631.72 (566.58 to 701.49) | 0.72 (0.37 to 1.25) | 1.61 (0.83 to 2.81) | 26.18 (13.8 to 45.37) | 26.18 (13.79 to 45.4) | |
| Andorra | 14.78 (11.7 to 18.34) | 37.71 (29.76 to 46.89) | 197.34 (155.87 to 245.23) | 106.2 (83.03 to 132.46) | 116.19 (100.92 to 132.7) | 306.31 (265.95 to 350.11) | 672.35 (571.63 to 785.86) | 718.66 (615.04 to 831.6) | 4.68 (2.43 to 7.95) | 12.25 (6.46 to 20.71) | 28.81 (14.79 to 50.43) | 28.69 (14.93 to 49.47) | |
| Angola | 249.85 (196.41 to 306.73) | 764.64 (600.98 to 938.07) | 60.09 (47.26 to 74.02) | 102.89 (80.04 to 127.84) | 1562.75 (1340.58 to 1806.31) | 4791.24 (4114.48 to 5539.88) | 670.47 (573.89 to 775.61) | 687.51 (583.59 to 805.06) | 64.6 (32.62 to 114.36) | 198.52 (100.9 to 352.14) | 27.16 (14.14 to 46.41) | 27.47 (14.26 to 47.93) | |
| Antigua and Barbuda | 9.89 (7.71 to 12.3) | 19.22 (14.94 to 23.86) | 143.44 (111.69 to 178.37) | 101.36 (79.73 to 125.43) | 72.83 (62.34 to 84.36) | 138.98 (119.02 to 160.87) | 721.94 (617.68 to 835.7) | 674.48 (577.65 to 780.29) | 2.92 (1.55 to 5.04) | 5.55 (2.95 to 9.54) | 27.07 (14.29 to 47.34) | 27.17 (14.21 to 47.4) | |
| Argentina | 7994.02 (6170.05 to 10030.61) | 13718.06 (10584.53 to 17216.92) | 190.16 (146.65 to 238.73) | 106.99 (83.51 to 133.35) | 64957.86 (55839.38 to 75060.55) | 113054.29 (97180.55 to 130611.77) | 691.87 (592.87 to 800.12) | 725.06 (620.36 to 839.43) | 2614.19 (1370.14 to 4424.24) | 4522.15 (2405.05 to 7764.49) | 28.94 (15.14 to 51.1) | 29.07 (15.17 to 50.27) |  |
| Armenia | 565.39 (427.67 to 719.16) | 1013.71 (766.93 to 1288.46) | 168.31 (127.42 to 214.27) | 103.28 (81.24 to 127.87) | 5324.89 (4589.51 to 6140.98) | 9549.42 (8231.14 to 11020.41) | 675.67 (610.41 to 747.24) | 690.46 (591.63 to 798.44) | 214.38 (111.46 to 366.3) | 382.41 (200.45 to 652.1) | 28.15 (14.6 to 48.69) | 28.17 (14.53 to 49.6) | |
| Australia | 3787.17 (2967.32 to 4743.34) | 8535.03 (6614.68 to 10663.98) | 145.6 (113.95 to 182.47) | 103.27 (88.52 to 119.77) | 25405.64 (21104.64 to 30107.1) | 57893.35 (48182.97 to 68739.3) | 681.52 (583.96 to 788.32) | 684.42 (618.12 to 757.4) | 1015.51 (536.36 to 1748.84) | 2303.94 (1194.82 to 3972.4) | 27.27 (14.61 to 46.62) | 27.58 (14.66 to 46.2) | |
| Austria | 3124.72 (2465.2 to 3897.31) | 4509.34 (3548.07 to 5618.97) | 198.55 (156.67 to 247.4) | 102.29 (80.51 to 126.59) | 25675.53 (22248.7 to 29352.63) | 37568.21 (32582.06 to 42941.46) | 853.17 (730.13 to 989.85) | 682.08 (584.52 to 788.88) | 1024.19 (546.97 to 1732.38) | 1495.65 (788.22 to 2537.3) | 27.56 (14.36 to 47.58) | 27.64 (14.34 to 48.31) | |
| Azerbaijan | 970.76 (734.7 to 1236.02) | 2115.35 (1599.58 to 2689.41) | 168.45 (127.51 to 214.73) | 120.73 (94.66 to 150.04) | 9204.58 (7930.34 to 10621.81) | 19696.25 (16985.33 to 22710.45) | 708.79 (607.42 to 820.47) | 850.6 (727.43 to 986.92) | 372.77 (195.76 to 624.94) | 796.66 (424.52 to 1364.01) | 34.49 (17.87 to 60.4) | 34.45 (17.73 to 58.97) | |
| Bahamas | 25.4 (19.79 to 31.58) | 70.93 (55.18 to 88.12) | 143.69 (111.89 to 178.78) | 104.63 (81.7 to 130.33) | 184.38 (157.77 to 213.71) | 512.89 (439.24 to 594.04) | 704.83 (604.3 to 816.53) | 705.47 (604.75 to 817.11) | 7.49 (3.99 to 12.98) | 20.56 (11.07 to 35.14) | 28.58 (14.49 to 49.95) | 28.49 (14.99 to 50.19) | |
| Bahrain | 12.81 (9.9 to 16.03) | 70.94 (54.83 to 88.79) | 71.37 (55.31 to 89.32) | 105.55 (82.52 to 131.59) | 83.59 (71.57 to 97.15) | 462.16 (395.89 to 537) | 1631.47 (1405.22 to 1884.79) | 713.03 (610.98 to 825.15) | 3.47 (1.78 to 6.17) | 18.91 (9.91 to 33.69) | 28.73 (14.74 to 50.56) | 28.82 (15.11 to 50.2) | |
| Bangladesh | 3887.73 (3044.78 to 4794.82) | 12415.42 (9732.34 to 15317.87) | 72.46 (56.74 to 89.45) | 168.28 (127.36 to 214.2) | 25044.67 (21322.58 to 29119.17) | 80078.88 (68175.79 to 93111.18) | 689.69 (591.09 to 797.64) | 1601.89 (1379.17 to 1850.74) | 1029.14 (534.04 to 1812.1) | 3306.29 (1710.36 to 5911.88) | 65.09 (34.64 to 111.67) | 63.99 (33.4 to 109.49) | |
| Barbados | 55.37 (43.24 to 68.82) | 99.87 (77.77 to 124.1) | 143.85 (112.08 to 178.88) | 103.96 (81.78 to 128.71) | 407.44 (348.74 to 472.15) | 727.92 (623.32 to 842.99) | 714.28 (611.83 to 826.74) | 695.96 (596.42 to 805.21) | 16.41 (8.61 to 28.26) | 29.2 (15.5 to 50.39) | 27.73 (14.57 to 48.66) | 27.89 (14.35 to 49.12) | |
| Belarus | 1727.74 (1472.66 to 2024.21) | 2202.42 (1876.73 to 2579.24) | 101.55 (86.41 to 119.09) | 106.09 (82.95 to 132.29) | 12059.25 (10845.81 to 13410.41) | 15277.78 (13740.81 to 16988.59) | 1615.42 (1391.13 to 1866.55) | 717.54 (614.17 to 830.37) | 489.67 (250.02 to 846.54) | 616.39 (312.74 to 1077.85) | 28.89 (14.87 to 51.15) | 28.85 (14.93 to 50.35) | |
| Belgium | 4054.34 (3200.8 to 5049.38) | 5862.42 (4613.74 to 7302.53) | 197.94 (156.23 to 246.36) | 167.94 (127.15 to 213.58) | 33049.25 (28674.1 to 37768.83) | 48748.6 (42279.44 to 55711.7) | 701.06 (601.49 to 812.43) | 1587.03 (1366.93 to 1833.19) | 1320 (696.72 to 2252.88) | 1929.05 (1016.71 to 3251.32) | 64.93 (34.55 to 109.22) | 63.92 (33.23 to 108.52) | |
| Belize | 16.2 (12.58 to 20.11) | 48.99 (38.03 to 60.78) | 142.19 (110.48 to 176.54) | 105.28 (82.32 to 131.21) | 118.17 (101.24 to 136.72) | 354.73 (303.91 to 410.23) | 716 (613.12 to 828.84) | 710.76 (609.15 to 822.51) | 4.8 (2.5 to 8.19) | 14.3 (7.59 to 24.81) | 28.53 (14.86 to 49.63) | 28.78 (14.87 to 50.75) | |
| Benin | 165.29 (130.02 to 204.77) | 402.14 (316.24 to 497.79) | 69.72 (54.81 to 86.4) | 105.2 (82.2 to 131.1) | 1109.01 (953.09 to 1278.21) | 2693.74 (2316.1 to 3103.75) | 713.66 (611.42 to 826.25) | 710.1 (608.42 to 821.83) | 45.54 (23.16 to 80.48) | 111.65 (58.21 to 195.89) | 28.91 (14.96 to 51.19) | 28.43 (15.06 to 50.05) | |
| Bermuda | 11.3 (8.8 to 14.04) | 25.84 (20.11 to 32.15) | 143.74 (111.91 to 178.89) | 105.6 (82.57 to 131.68) | 82.03 (70.2 to 95.07) | 190.06 (162.68 to 220.14) | 1586.68 (1366.65 to 1833.6) | 713.53 (611.28 to 825.84) | 3.34 (1.75 to 5.83) | 7.62 (3.98 to 12.93) | 28.88 (15.21 to 49.73) | 28.73 (14.8 to 50.34) | |
| Bhutan | 19.49 (15.26 to 24.06) | 53.17 (41.68 to 65.63) | 73.4 (57.5 to 90.65) | 167.06 (126.75 to 212.15) | 124.87 (106.24 to 145.29) | 343.93 (292.94 to 399.94) | 681.14 (583.63 to 788.03) | 1549.45 (1336.1 to 1789.68) | 5.19 (2.73 to 9.22) | 14.21 (7.16 to 25.17) | 64.1 (33.47 to 110.8) | 62.53 (32.89 to 107.58) | |
| Bolivia (Plurinational State of) | 502.59 (410.14 to 613.49) | 1502.38 (1225.38 to 1833.37) | 134.02 (108.97 to 163.82) | 103.18 (81.19 to 127.7) | 3521.37 (3098.02 to 3995.29) | 10536.82 (9269.23 to 11955.79) | 881.71 (750.18 to 1026.98) | 689.51 (590.9 to 797.48) | 141.69 (74.17 to 242.29) | 420.57 (216.65 to 721.77) | 27.52 (14.49 to 48.47) | 27.73 (14.46 to 48.3) | |
| Bosnia and Herzegovina | 424.61 (333.39 to 521.97) | 733.12 (577.44 to 899.57) | 87.26 (68.66 to 107.22) | 116.67 (90.38 to 147.04) | 2715.7 (2337.81 to 3139.21) | 4669.73 (4017.51 to 5388.01) | 1175.42 (993.63 to 1380.81) | 877.09 (746.34 to 1021.55) | 112.35 (57.72 to 198.01) | 191.75 (98.3 to 343.38) | 35.16 (18.39 to 60.25) | 35.23 (18.12 to 62.07) | |
| Botswana | 44.15 (34.52 to 54.46) | 111.56 (87.2 to 137.73) | 65.94 (51.55 to 81.44) | 150.26 (114 to 190.16) | 281.98 (240.79 to 325.52) | 714.39 (610.08 to 824.22) | 543.68 (467.7 to 627.11) | 1213.3 (1027.3 to 1418.31) | 11.78 (6.01 to 20.9) | 29.44 (14.93 to 51.97) | 47.62 (24.93 to 82.36) | 48.91 (25.4 to 84.07) | |
| Brazil | 17934.91 (15631.39 to 20568.2) | 53586.93 (46653.66 to 61459.77) | 169.19 (147.27 to 194.03) | 85.69 (67.49 to 105.16) | 148903.3 (135693.01 to 163402.99) | 449806.98 (409983.66 to 493469.8) | 1607.28 (1384.44 to 1857.34) | 542.93 (466.82 to 626.33) | 5900.58 (3163.59 to 9701.4) | 17766.65 (9458.69 to 29346.6) | 22.51 (11.69 to 40.26) | 22.56 (11.72 to 39.91) | |
| Brunei Darussalam | 21.57 (16.59 to 27) | 80.71 (61.95 to 100.96) | 200 (153.9 to 250.55) | 167.93 (127.17 to 213.55) | 179.2 (153.51 to 206.98) | 659.37 (564.54 to 761.35) | 546.04 (469.44 to 629.99) | 1583.98 (1364.61 to 1829.58) | 7.18 (3.81 to 12.17) | 26.39 (13.75 to 44.41) | 64.81 (34.27 to 109.89) | 63.78 (33.54 to 108.09) | |
| Bulgaria | 1464.7 (1152.7 to 1799.47) | 1679.49 (1324.73 to 2059.57) | 86.24 (67.89 to 105.85) | 85.76 (67.53 to 105.26) | 9276.71 (7979.64 to 10710.17) | 10717.37 (9217.64 to 12368.46) | 644.88 (551.57 to 745.64) | 544.23 (468.07 to 627.74) | 385.43 (198.28 to 674.01) | 440.38 (228.65 to 767.36) | 22.48 (11.58 to 40.53) | 22.32 (11.47 to 39.69) | |
| Burkina Faso | 363.32 (285.9 to 450) | 740.95 (582.74 to 917.26) | 69.79 (54.86 to 86.49) | 100.32 (78.97 to 124.12) | 2424.41 (2083.79 to 2793.93) | 4958.11 (4261.59 to 5713.38) | 676.65 (579.64 to 782.76) | 665.7 (569.42 to 770.01) | 100.81 (51.31 to 177.25) | 205.63 (104.68 to 362.27) | 26.39 (13.52 to 45.75) | 27.17 (13.84 to 47.93) | |
| Burundi | 186.54 (147.93 to 228.14) | 373.93 (296.57 to 457.49) | 68.73 (54.4 to 84.2) | 101.25 (79.68 to 125.24) | 1239.49 (1066.15 to 1429.95) | 2465.32 (2121 to 2843.62) | 2361.12 (1983.31 to 2775.26) | 673.25 (576.53 to 778.77) | 51.42 (27 to 90.58) | 103.25 (53.29 to 182.6) | 27.37 (13.95 to 48.57) | 27.13 (13.99 to 47.8) | |
| Cabo Verde | 20.6 (16.18 to 25.49) | 37.85 (29.74 to 46.86) | 70.3 (55.23 to 87.01) | 249.97 (189.38 to 321.84) | 139.63 (120.08 to 161.06) | 254.85 (219.23 to 293.78) | 1605.14 (1381.98 to 1855.12) | 2344.73 (1969.09 to 2756.95) | 5.8 (2.97 to 10.34) | 10.6 (5.44 to 18.51) | 94.02 (49.14 to 160.15) | 93.65 (49.64 to 156.92) | |
| Cambodia | 526.66 (414.34 to 652.08) | 1533.85 (1206.47 to 1899.52) | 103.21 (81.21 to 127.72) | 167.62 (127 to 213.02) | 3476.58 (2979.95 to 4017.27) | 10151.91 (8701.73 to 11736.53) | 1611.26 (1382.26 to 1856.45) | 1575.93 (1357.82 to 1820.52) | 140.9 (74.29 to 248.24) | 409.76 (210.91 to 720.88) | 64.82 (33.97 to 108.67) | 63.33 (33.68 to 107.85) | |
| Cameroon | 400.05 (312.47 to 496.35) | 1091.48 (852.16 to 1363.08) | 77.92 (60.85 to 96.93) | 168.13 (128.03 to 213.89) | 2829.35 (2418.15 to 3278.72) | 7723.99 (6603.6 to 9008.86) | 1647.73 (1431.49 to 1885.21) | 1594.85 (1374.42 to 1835.47) | 117.56 (61.42 to 207.67) | 320.69 (164.26 to 569.72) | 64.93 (34.81 to 109.12) | 63.73 (33.92 to 108.24) | |
| Canada | 9247.47 (7410.55 to 11267.96) | 20794.67 (16650.24 to 25374.85) | 216.67 (173.5 to 264.2) | 199.33 (155.91 to 249.02) | 88726.32 (79233.1 to 99065.62) | 200986.04 (179416.91 to 224411.61) | 712.76 (610.69 to 825.02) | 1622.72 (1407.87 to 1854.62) | 3578.73 (1901.15 to 6072.2) | 8033.76 (4254.83 to 13642.01) | 65.85 (34.44 to 112.1) | 64.75 (33.74 to 111.04) | |
| Central African Republic | 75.93 (59.67 to 93.2) | 138.15 (108.56 to 169.34) | 60.45 (47.53 to 74.46) | 105.92 (82.82 to 132.08) | 474.38 (407.04 to 548.39) | 863.42 (741.47 to 998.29) | 1568.67 (1351.36 to 1812.57) | 716.19 (613.24 to 828.77) | 19.54 (10.12 to 34.56) | 35.61 (18 to 62.41) | 28.94 (15.19 to 50.32) | 28.81 (14.87 to 49.98) | |
| Chad | 235.81 (185.56 to 292.05) | 442.01 (347.74 to 548.07) | 69.71 (54.82 to 86.37) | 168.45 (127.47 to 214.28) | 1580.77 (1358.35 to 1822.57) | 2950.03 (2538.78 to 3401.32) | 562.7 (484.11 to 650.09) | 1596.34 (1375.27 to 1843.92) | 65.37 (33.58 to 115.85) | 122.2 (63.36 to 212.92) | 63.03 (33.65 to 106.8) | 64.09 (33.77 to 108.4) | |
| Chile | 2348.02 (1811.58 to 2945.35) | 6300.1 (4856.11 to 7906.84) | 190.12 (146.62 to 238.66) | 86.3 (67.96 to 105.93) | 19090.92 (16414.56 to 22052.51) | 51821.92 (44562.73 to 59849.31) | 548.79 (471.96 to 633.17) | 550.15 (473.17 to 635) | 765.61 (401.11 to 1320.38) | 2062.21 (1082.2 to 3546.42) | 23.21 (12.22 to 40.83) | 22.67 (11.7 to 40.4) | |
| China | 100678.48 (86228.74 to 115833.85) | 270331.98 (232354.22 to 312366.65) | 99.77 (85.3 to 114.87) | 86.61 (68.19 to 106.33) | 627524.41 (564881.95 to 695852.7) | 1700330.02 (1524724.81 to 1888313.9) | 1601.9 (1388.73 to 1830.33) | 553.25 (475.97 to 638.76) | 26197.69 (13816.37 to 45238.24) | 70598.84 (37190.42 to 122155.3) | 22.73 (11.72 to 39.79) | 22.76 (11.82 to 39.69) | |
| Colombia | 2597.6 (2159.12 to 3088.21) | 8927.11 (7405.31 to 10640.82) | 127.92 (106.14 to 152.26) | 197.53 (155.96 to 245.59) | 17786.04 (15793.35 to 19923.94) | 61765.63 (54794.95 to 69225.53) | 1778.45 (1602.19 to 1979.91) | 1591.42 (1381.39 to 1817.9) | 716.71 (367.3 to 1209.93) | 2473.11 (1288.49 to 4224.66) | 64.51 (33.7 to 111.02) | 63.82 (33.15 to 107.44) | |
| Comoros | 15.75 (12.5 to 19.26) | 38.94 (30.87 to 47.65) | 68.35 (54.13 to 83.73) | 212.82 (179.09 to 250.61) | 104.02 (89.44 to 119.94) | 258.61 (222.5 to 298.35) | 1596.82 (1442.16 to 1761.59) | 1768.72 (1589.83 to 1958.74) | 4.33 (2.3 to 7.73) | 10.72 (5.47 to 18.98) | 70.74 (37.55 to 117.74) | 70.86 (37.54 to 118.97) | |
| Congo | 62.15 (49.01 to 77.29) | 140.87 (111.2 to 175.29) | 49.67 (39.15 to 61.82) | 199.02 (170.17 to 230.37) | 364.59 (307.67 to 431.07) | 826.67 (697.7 to 975) | 557.98 (479.99 to 644.48) | 1590.03 (1437.02 to 1754.11) | 15.08 (7.6 to 27.04) | 34.34 (17.27 to 61.73) | 64.14 (34.34 to 107.71) | 63.83 (33.9 to 108.01) | |
| Cook Islands | 1.55 (1.21 to 1.93) | 3.6 (2.81 to 4.48) | 104.88 (81.96 to 130.71) | 86.13 (67.82 to 105.71) | 10.35 (8.88 to 11.99) | 24.26 (20.8 to 28.08) | 1602.68 (1389.36 to 1831.28) | 548.27 (471.59 to 632.65) | 0.42 (0.22 to 0.73) | 0.98 (0.51 to 1.7) | 22.95 (11.88 to 40.8) | 22.5 (11.38 to 39.78) | |
| Costa Rica | 267.27 (221.94 to 317.94) | 895.14 (742.9 to 1066.49) | 127.89 (106.13 to 152.22) | 197.55 (155.96 to 245.61) | 1838.43 (1632.45 to 2059.57) | 6184.44 (5487.08 to 6931.25) | 1605.9 (1383.24 to 1855.88) | 1592.48 (1382.16 to 1819.1) | 74.06 (38.81 to 126.05) | 247.1 (124.77 to 427.91) | 63.93 (34.15 to 107.47) | 63.62 (33.96 to 107.64) | |
| Coted'Ivoire | 294.05 (231.42 to 364.4) | 836.22 (658.21 to 1036.02) | 69.16 (54.38 to 85.78) | 167.45 (126.91 to 212.77) | 1954.19 (1682.34 to 2251.56) | 5575.37 (4798.64 to 6423.55) | 558.87 (480.89 to 645.32) | 1569.76 (1352.73 to 1813.31) | 80.32 (42.27 to 139.04) | 231.02 (117.5 to 403.23) | 64.71 (33.85 to 109.02) | 63.03 (32.99 to 108.89) | |
| Croatia | 692.65 (544.07 to 851.15) | 1030.67 (812.94 to 1263.74) | 87.53 (68.82 to 107.51) | 86.4 (68.03 to 106.07) | 4446.8 (3828.02 to 5139.66) | 6576.69 (5657.2 to 7587.32) | 554.4 (477.02 to 640.04) | 550.78 (473.76 to 635.64) | 184.19 (97.44 to 323.08) | 270.38 (139.64 to 482.32) | 23.03 (11.85 to 40.58) | 22.61 (11.58 to 40.48) | |
| Cuba | 1837.77 (1430.78 to 2279.84) | 3463.01 (2694.26 to 4306.09) | 142.32 (110.65 to 176.75) | 86.23 (67.9 to 105.84) | 13383.97 (11462.61 to 15485.4) | 25442.88 (21777.23 to 29461.1) | 559.45 (503.14 to 620.05) | 549.32 (472.54 to 633.93) | 543.96 (285.29 to 933.85) | 1020.83 (526.96 to 1731.35) | 22.99 (11.87 to 40.62) | 22.69 (11.61 to 40.22) | |
| Cyprus | 201.53 (153.35 to 257.43) | 499.54 (379.68 to 639.29) | 180.83 (137.24 to 231.67) | 86.88 (74.82 to 100.5) | 1469 (1169.71 to 1797.32) | 3761.71 (2996.73 to 4599.83) | 564.79 (485.83 to 652.33) | 552.72 (497.36 to 612.44) | 58.9 (31.05 to 99.71) | 151.12 (78.86 to 260.23) | 22.91 (11.97 to 40.26) | 22.76 (11.94 to 39.69) | |
| Czechia | 1606.46 (1264.7 to 1974.5) | 2432.06 (1918.36 to 2984.63) | 87.05 (68.49 to 106.93) | 85.81 (67.58 to 105.31) | 10284.63 (8851.26 to 11884.15) | 15471.79 (13299.24 to 17843.58) | 366.14 (311.78 to 427.87) | 545.09 (468.76 to 628.79) | 423.54 (218.91 to 753.39) | 633.08 (320.25 to 1116.61) | 23.12 (12.08 to 40.42) | 22.42 (11.64 to 40.38) | |
| Democratic People's Republic of Korea | 2208.02 (1730.68 to 2746.28) | 4834.78 (3792.31 to 6006.27) | 121.04 (94.9 to 150.51) | 63.98 (50.76 to 78.72) | 15383.34 (13165.92 to 17851.81) | 33898.97 (28996.87 to 39334.29) | 549.68 (472.63 to 634.53) | 365.53 (312.14 to 427.39) | 625.84 (324.69 to 1100.66) | 1376.28 (709.16 to 2355.49) | 15.13 (7.68 to 26.9) | 15.13 (7.6 to 26.65) | |
| Democratic Republic of the Congo | 1085.59 (853.04 to 1331.47) | 2413.74 (1897.53 to 2962.06) | 60.24 (47.38 to 74.12) | 86.21 (67.88 to 105.82) | 6786.33 (5825.31 to 7850.04) | 15130.93 (12985.36 to 17490.82) | 1491.78 (1356.39 to 1638.11) | 548.76 (471.91 to 633.22) | 279.28 (143.39 to 487.89) | 624.91 (317.52 to 1096.17) | 22.67 (11.86 to 40.16) | 22.54 (11.76 to 39.86) | |
| Denmark | 2546.31 (1901.34 to 3272.97) | 3698.17 (2762.63 to 4749.88) | 237.89 (177.72 to 305.57) | 146.55 (122.08 to 173.38) | 23305.75 (19274.19 to 27846.82) | 33832.91 (27953.24 to 40449.95) | 556.16 (478.31 to 642.14) | 1457.57 (1324.88 to 1600.46) | 927.93 (488.14 to 1532.91) | 1360.2 (714.37 to 2336.58) | 59.53 (30.95 to 102) | 58.43 (30.62 to 101.11) | |
| Djibouti | 9.51 (7.55 to 11.64) | 46.47 (36.87 to 56.9) | 68.26 (54.06 to 83.65) | 86.51 (68.11 to 106.19) | 62.57 (53.82 to 72.15) | 305.78 (263.02 to 352.81) | 1126.74 (1037.49 to 1223.5) | 552.56 (475.3 to 637.97) | 2.6 (1.33 to 4.69) | 12.77 (6.63 to 22.64) | 22.78 (11.7 to 40.2) | 22.67 (11.98 to 39.48) | |
| Dominica | 11.24 (8.77 to 13.97) | 15.43 (11.99 to 19.17) | 144.35 (112.51 to 179.56) | 130.79 (114.75 to 148.87) | 81.9 (70.09 to 94.97) | 111.95 (95.91 to 129.56) | 709.45 (640.71 to 782.67) | 1146.5 (1057.69 to 1244.37) | 3.3 (1.74 to 5.68) | 4.49 (2.37 to 7.77) | 44.85 (24.06 to 74.42) | 45.5 (24.36 to 74.95) | |
| Dominican Republic | 621.81 (483.46 to 771.15) | 1728.64 (1344.15 to 2147.55) | 142.21 (110.57 to 176.6) | 101.03 (87.87 to 115.66) | 4490.41 (3846.16 to 5194.71) | 12612.37 (10799.49 to 14597.64) | 983.55 (817.31 to 1165.29) | 696.11 (629.35 to 767.34) | 182.41 (96.35 to 307.92) | 506.41 (267.14 to 865.51) | 28.57 (14.81 to 48.61) | 27.93 (14.47 to 48.27) | |
| Ecuador | 834.45 (664.11 to 1017.56) | 2745.55 (2184.5 to 3350.82) | 134.91 (107.29 to 164.7) | 145.15 (112.42 to 181.34) | 5924.44 (5121.28 to 6804.39) | 19539.9 (16886.1 to 22447.2) | 707.28 (635.91 to 786.69) | 978.23 (814.3 to 1160.98) | 238.86 (125 to 403.1) | 779.36 (406.48 to 1314.74) | 39.29 (20.74 to 67.75) | 39.05 (20.28 to 67.32) | |
| Egypt | 1418.15 (1110.49 to 1773.57) | 3454.09 (2691.38 to 4316.12) | 46.7 (36.59 to 58.46) | 100.33 (85.48 to 117.45) | 8109.41 (6794.44 to 9618.47) | 19730.43 (16485.32 to 23431.56) | 1683.44 (1442.16 to 1944.26) | 695.11 (625.04 to 772.94) | 338.84 (168.06 to 605.29) | 814.12 (416.68 to 1454.51) | 28.69 (14.65 to 49.73) | 28.01 (14.19 to 48.84) | |
| El Salvador | 453.72 (376.77 to 540.23) | 985.54 (816.21 to 1176.27) | 128.25 (106.47 to 152.69) | 200.24 (154.08 to 250.92) | 3127.25 (2775.48 to 3503.78) | 6851.72 (6074.19 to 7678.84) | 699.88 (629.39 to 778.11) | 1688.77 (1446.62 to 1949.2) | 125.58 (66.22 to 218.75) | 274.88 (142.27 to 473.09) | 67.24 (35.71 to 113.96) | 67.02 (34.87 to 112.49) | |
| Equatorial Guinea | 13.22 (10.39 to 16.23) | 32.41 (25.49 to 39.76) | 60.57 (47.62 to 74.58) | 100.11 (85.31 to 117.13) | 82.87 (71.12 to 95.84) | 203.79 (174.92 to 235.61) | 692.47 (622.74 to 769.87) | 691.9 (622.15 to 769.31) | 3.4 (1.74 to 6.04) | 8.4 (4.25 to 14.8) | 28.21 (14.56 to 48.57) | 27.9 (14.59 to 47.81) | |
| Eritrea | 75.65 (59.98 to 92.53) | 201.38 (159.66 to 246.53) | 69.44 (54.94 to 85.18) | 99.88 (85.18 to 116.83) | 495.99 (426.55 to 572.35) | 1328.67 (1142.49 to 1533.46) | 293.27 (263.68 to 325.84) | 688.65 (619.26 to 765.61) | 20.63 (10.68 to 36.25) | 54.94 (28.18 to 97.95) | 27.9 (14.11 to 48.24) | 27.84 (14.32 to 48.7) | |
| Estonia | 396.46 (329.86 to 469.92) | 518.65 (431.28 to 614.3) | 148.21 (123.21 to 175.75) | 53.5 (46.12 to 61.35) | 3998.43 (3637.46 to 4389.72) | 5170.96 (4697.81 to 5681.8) | 2126.92 (1923.78 to 2344.32) | 285.93 (257.08 to 318) | 159.73 (83.01 to 273.55) | 206.42 (108.14 to 356.32) | 12.07 (5.94 to 21.74) | 11.83 (5.95 to 21.37) | |
| Eswatini | 20.89 (16.33 to 25.77) | 42.77 (33.43 to 52.84) | 66.15 (51.7 to 81.73) | 230.87 (193.93 to 271.79) | 133.95 (114.44 to 154.74) | 273.65 (233.69 to 316.04) | 1811.02 (1539.13 to 2112.61) | 2120.66 (1917.84 to 2336.51) | 5.56 (2.86 to 9.8) | 11.28 (5.92 to 19.76) | 85.84 (45.82 to 142.18) | 85.99 (46.14 to 143.28) | |
| Ethiopia | 1436.49 (1230.09 to 1672.87) | 3096.55 (2650.41 to 3605.77) | 63.51 (54.27 to 73.98) | 206.73 (158.7 to 261.32) | 9094.59 (8192.86 to 10122.1) | 19783.22 (17819.77 to 21992.21) | 1329.24 (1099.61 to 1583.44) | 1791.68 (1519.66 to 2087.71) | 375.31 (198.27 to 648.24) | 815.99 (429.93 to 1428.93) | 72.38 (37.89 to 123.72) | 71.85 (37.86 to 122.54) | |
| Fiji | 39.35 (30.74 to 49.04) | 98.39 (76.85 to 122.62) | 105.52 (82.49 to 131.57) | 174.23 (134.19 to 220.7) | 262.03 (224.57 to 303.08) | 655.35 (561.24 to 757.78) | 1602.04 (1388.86 to 1830.45) | 1326.56 (1099.55 to 1578.59) | 10.65 (5.48 to 18.64) | 26.38 (13.8 to 45.36) | 53.76 (27.44 to 91.94) | 53.92 (27.93 to 93.49) | |
| Finland | 1876.19 (1481.32 to 2336.12) | 3297.82 (2596.74 to 4106.74) | 198.3 (156.5 to 246.96) | 197.61 (156 to 245.7) | 15237.9 (13199.44 to 17417.35) | 27200.11 (23581.21 to 31099.08) | 1609.78 (1394.29 to 1839.47) | 1593.78 (1382.97 to 1820.61) | 605 (313.03 to 1024.14) | 1078.19 (573.54 to 1821.66) | 64.06 (33.86 to 109.22) | 63.41 (33.38 to 106.95) | |
| France | 27154.79 (20605.7 to 35073.15) | 44268.68 (33431.84 to 57069.88) | 250.61 (189.88 to 322.88) | 197.68 (156.03 to 245.81) | 261653.87 (220024.54 to 307001.52) | 431266.12 (362198.19 to 507419.03) | 2150.62 (1778.56 to 2568.72) | 1594.65 (1383.65 to 1821.57) | 10388.55 (5428.58 to 17713.02) | 17102.54 (9069.48 to 28741.51) | 63.95 (33.1 to 108.42) | 63.58 (33.81 to 107.79) | |
| Gabon | 52.64 (40.12 to 66.09) | 90.57 (70.01 to 113.92) | 74.39 (56.68 to 93.51) | 237.31 (177.39 to 304.67) | 357.03 (298.91 to 421.52) | 611.52 (514.13 to 721.22) | 1367.22 (1089.02 to 1672.56) | 2136.61 (1766.51 to 2552.22) | 14.75 (7.78 to 25.71) | 25.19 (13.09 to 44.99) | 85.87 (45.13 to 142.04) | 86.21 (45.18 to 148.21) | |
| Gambia | 27.3 (21.48 to 33.82) | 76.64 (60.27 to 94.9) | 69.36 (54.51 to 85.98) | 180.35 (136.91 to 231.1) | 182.29 (156.8 to 210.13) | 513.99 (442.01 to 592.26) | 1316.86 (1054.81 to 1609.81) | 1361.05 (1084 to 1664.15) | 7.6 (3.9 to 13.44) | 21.25 (10.96 to 38.17) | 54.59 (28.75 to 92.44) | 54.67 (28.54 to 94.28) | |
| Georgia | 1375.44 (1046.98 to 1739.9) | 1359.77 (1034.77 to 1728.95) | 168.54 (128.5 to 213.52) | 176.44 (134.38 to 226.84) | 13082.7 (11233.44 to 15058.58) | 12925.02 (11143.26 to 14873.49) | 1612.91 (1396.5 to 1843.09) | 1313.47 (1053.5 to 1606.14) | 528.97 (283.96 to 888.86) | 516.33 (274.48 to 877.81) | 52.65 (27.06 to 88.68) | 52.81 (27.54 to 90.23) | |
| Germany | 33058.5 (25854.91 to 41305.96) | 48927.01 (38110.53 to 61207.1) | 201.31 (157.5 to 251.1) | 197.74 (156.09 to 245.92) | 274446.02 (238362.49 to 314229.73) | 415283.99 (359566.42 to 475540.53) | 1586.32 (1377.3 to 1811.92) | 1596.03 (1384.66 to 1823.24) | 10947.96 (5724.26 to 18657.25) | 16456.56 (8559.91 to 28245.86) | 64.48 (34.46 to 109.26) | 63.89 (33.73 to 108.2) | |
| Ghana | 434.52 (339.27 to 537.73) | 1185.26 (926.16 to 1468.44) | 61.61 (48.15 to 76.36) | 197.05 (155.67 to 244.77) | 2729.34 (2323.56 to 3170.12) | 7454.38 (6347.38 to 8664.44) | 1487.7 (1344.13 to 1647.32) | 1583.09 (1374.97 to 1809.05) | 113.87 (59.12 to 199.21) | 310.7 (156.29 to 559.65) | 63.64 (33.05 to 108.25) | 63.47 (33.48 to 107.31) | |
| Greece | 3563.38 (2721.61 to 4530.67) | 5224.6 (3967.8 to 6727.32) | 176.47 (134.62 to 224.63) | 187.09 (158.9 to 218.31) | 26506.07 (21235.01 to 32393.48) | 40804.54 (32638.75 to 49972.01) | 1597.46 (1385.62 to 1825.23) | 1485.12 (1333.47 to 1640.54) | 1060.47 (545.44 to 1786.87) | 1627.52 (847.02 to 2790.43) | 59.05 (31.38 to 100.82) | 59.36 (31.15 to 99.48) | |
| Greenland | 7.77 (6.24 to 9.47) | 19.26 (15.52 to 23.5) | 216.34 (173.32 to 263.73) | 197.58 (156.01 to 245.68) | 72.84 (65.05 to 81.3) | 178.19 (158.99 to 198.88) | 1564.47 (1344.84 to 1807.38) | 1591.58 (1381.68 to 1818.02) | 2.93 (1.56 to 4.92) | 7.19 (3.84 to 12.27) | 64 (33.47 to 107.72) | 64.03 (33.62 to 108.68) | |
| Grenada | 13.55 (10.56 to 16.87) | 20.02 (15.55 to 24.85) | 143.84 (112.05 to 178.93) | 190.07 (146.55 to 238.57) | 100.16 (85.72 to 116.06) | 144.38 (123.61 to 167.14) | 2041.72 (1842.68 to 2262.87) | 1562.76 (1343.53 to 1805.13) | 4.04 (2.1 to 6.94) | 5.76 (3.07 to 9.97) | 62.83 (32.93 to 106.4) | 62.58 (33.28 to 107.48) | |
| Guam | 9.33 (7.28 to 11.63) | 29.13 (22.71 to 36.41) | 105.31 (82.36 to 131.3) | 232.53 (196.06 to 274.15) | 61.9 (53.08 to 71.61) | 196.57 (168.3 to 227.5) | 1601.06 (1388.75 to 1829.07) | 2034.41 (1835.62 to 2253.32) | 2.55 (1.31 to 4.48) | 8.03 (4.22 to 13.97) | 81.15 (43.6 to 134.25) | 81.36 (43.5 to 135.48) | |
| Guatemala | 508.26 (423.01 to 603.49) | 1716.56 (1427.5 to 2040.92) | 127.64 (105.87 to 151.92) | 197.55 (155.96 to 245.62) | 3456.46 (3070.51 to 3871.52) | 11786.27 (10464.69 to 13200.6) | 1835.75 (1505.4 to 2205.44) | 1592.21 (1381.9 to 1818.89) | 138.59 (71.71 to 236.7) | 465.35 (243.43 to 802.13) | 64.41 (33.43 to 108.94) | 64.07 (33.18 to 107.25) | |
| Guinea | 287.27 (226.07 to 356.03) | 452.4 (356.09 to 560.73) | 69.51 (54.66 to 86.19) | 215.07 (163.37 to 275.8) | 1921.27 (1651.22 to 2213.86) | 3027.23 (2604.06 to 3488.57) | 1564.11 (1344.45 to 1807.01) | 1826.2 (1500.29 to 2187.61) | 79.72 (40.44 to 138.67) | 125.37 (63.9 to 218.31) | 73.86 (39.04 to 126.7) | 73.52 (38.66 to 124.33) | |
| Guinea-Bissau | 31.77 (25.01 to 39.35) | 54.48 (42.86 to 67.41) | 69.59 (54.73 to 86.25) | 190.15 (146.64 to 238.74) | 211.52 (181.85 to 243.78) | 362.67 (311.82 to 417.87) | 1606.62 (1391.49 to 1836.47) | 1564.63 (1344.86 to 1807.69) | 8.77 (4.41 to 15.62) | 15.04 (7.75 to 26.57) | 62.98 (32.56 to 106) | 62.64 (33.08 to 106.98) | |
| Guyana | 63.17 (49.11 to 78.43) | 112.53 (87.5 to 139.74) | 142.68 (110.97 to 177.3) | 197.41 (155.86 to 245.37) | 455.88 (390.44 to 527.6) | 809.69 (693.39 to 937.58) | 2089.23 (1865.56 to 2332.36) | 1590.35 (1380.41 to 1817.03) | 18.09 (9.67 to 30.75) | 31.96 (16.94 to 55.22) | 64.44 (33.53 to 110.06) | 63.89 (33.81 to 109.39) | |
| Haiti | 539.14 (418.72 to 668.7) | 1158.53 (900.8 to 1437.47) | 142.62 (110.86 to 177.18) | 216.77 (173.66 to 264.48) | 3850.71 (3298.52 to 4457.34) | 8294.43 (7104.9 to 9600.1) | 1453 (1161.79 to 1773.55) | 2083.75 (1860.25 to 2326.3) | 154.97 (81.1 to 266.67) | 330.85 (174.98 to 558.43) | 84.25 (44.76 to 143.01) | 83.45 (44.21 to 141.75) | |
| Honduras | 302.01 (251.18 to 358.86) | 970.56 (807.7 to 1153.15) | 127.83 (106.06 to 152.16) | 187.33 (141.63 to 240.97) | 2063.93 (1833.29 to 2311.63) | 6622.87 (5881.04 to 7416.36) | 2254.65 (2071.95 to 2447.21) | 1448.03 (1157.96 to 1768.12) | 82.97 (42.5 to 141.79) | 264.08 (140.32 to 454.19) | 58.06 (30.1 to 99.72) | 58.04 (29.6 to 100.63) | |
| Hungary | 2316.58 (1795.89 to 2920.82) | 3053.06 (2367.76 to 3850.51) | 117.05 (90.66 to 147.52) | 227.74 (196.59 to 262.84) | 17428.7 (14838.26 to 20297.26) | 22921.81 (19503.69 to 26708.47) | 1602.38 (1389.34 to 1830.83) | 2270.04 (2084.45 to 2469.8) | 696.15 (364.31 to 1191.67) | 918.39 (470.61 to 1615.47) | 89.73 (48.01 to 147.84) | 88.86 (47.55 to 145.98) | |
| Iceland | 79.96 (60.45 to 102.48) | 162.57 (123.35 to 208.59) | 215.69 (163.12 to 276.37) | 197.99 (156.28 to 246.41) | 685.69 (562.21 to 824.05) | 1395.89 (1145.99 to 1672.86) | 1601.65 (1389.51 to 1829.49) | 1599.47 (1387.36 to 1827.25) | 27.54 (14.55 to 47.22) | 56.07 (29.5 to 94.84) | 63.97 (33.65 to 107.67) | 63.91 (33.76 to 107.15) | |
| India | 38400.33 (32899.22 to 44692.68) | 108337.77 (92763.57 to 125951.47) | 73.66 (63.03 to 85.62) | 197.86 (156.18 to 246.19) | 244555.94 (220945.39 to 270851.11) | 694386.99 (626995.9 to 768757.37) | 1779.1 (1607.49 to 1966.43) | 1596.82 (1385.7 to 1824.01) | 9989.9 (5220.55 to 17396.89) | 28394.27 (14803.32 to 49641.19) | 64.39 (33.69 to 109.16) | 64.19 (33.75 to 107.9) | |
| Indonesia | 11193.24 (9570.64 to 13072.18) | 29024.5 (24828.28 to 33551.43) | 102.3 (87.34 to 119.24) | 211.91 (178.22 to 247.77) | 73192.01 (65757.46 to 81383.92) | 189339.91 (170566.61 to 209695.2) | 1049.43 (898.4 to 1215.49) | 1776.94 (1610.34 to 1965.28) | 2987.24 (1577.33 to 5076.82) | 7730.76 (4148.38 to 13056.92) | 71.45 (38.07 to 118.9) | 71.06 (37.91 to 117.93) | |
| Iran (Islamic Republic of) | 2468.13 (2048.41 to 2922.82) | 7309.29 (6135.11 to 8667.15) | 78.89 (65.51 to 93.28) | 142.7 (110.97 to 177.33) | 16547.93 (14691.44 to 18565.68) | 49417 (43874.02 to 55405.26) | 1563.45 (1344.16 to 1805.87) | 1043.19 (893.3 to 1207.39) | 688.04 (363.46 to 1183.48) | 2035.87 (1085.81 to 3563.93) | 42.21 (22.39 to 72.86) | 41.53 (22.04 to 71.34) | |
| Iraq | 649.22 (501.69 to 812.66) | 1905.98 (1472.04 to 2385.53) | 71.74 (55.47 to 89.79) | 190.06 (146.54 to 238.55) | 4275.48 (3659.56 to 4964.15) | 12490.9 (10694.06 to 14507.34) | 1051.28 (899.68 to 1218.14) | 1561.95 (1343.14 to 1803.93) | 176.31 (89.64 to 315.39) | 509.65 (262.46 to 898.5) | 62.56 (32.78 to 107.84) | 62.16 (32.61 to 106.9) | |
| Ireland | 1090.18 (861.58 to 1354.8) | 2025.57 (1598.44 to 2519.46) | 197.79 (156.12 to 246.01) | 143.36 (111.63 to 178.23) | 8711.82 (7555.23 to 9959.36) | 16425.79 (14254.19 to 18771.11) | 930.78 (816.58 to 1056.09) | 1048.16 (897.42 to 1214.17) | 349.07 (182.5 to 586.65) | 659.61 (346.27 to 1119.01) | 42.62 (22.71 to 73.95) | 41.89 (22.49 to 71.64) | |
| Israel | 1286.17 (1016.49 to 1601.67) | 3164.48 (2494.95 to 3939.37) | 198.24 (156.48 to 247.01) | 132.31 (106.14 to 160.72) | 10298.37 (8937.18 to 11766.82) | 25721.57 (22309.11 to 29392.94) | 1052.45 (901.02 to 1219.34) | 931.65 (817.4 to 1057.09) | 414.29 (216.66 to 701.85) | 1031.42 (542.85 to 1730.27) | 37.6 (19.72 to 64.25) | 37.55 (19.73 to 63.46) | |
| Italy | 27771.03 (23446.72 to 32752.37) | 41896.67 (35207.51 to 49452.15) | 232.73 (196.37 to 274.59) | 143.16 (111.46 to 177.93) | 243988.32 (220178.61 to 270349.48) | 384648.88 (347214.33 to 425475.69) | 1038.65 (889.92 to 1201.61) | 1046.4 (896.04 to 1211.73) | 9697.15 (5200.61 to 16057.91) | 15257.54 (8164.96 to 25425.34) | 42.54 (22.37 to 72.99) | 41.95 (22.25 to 72.37) | |
| Jamaica | 329.18 (256.31 to 409.1) | 552.39 (428.49 to 687.48) | 142.94 (111.22 to 177.65) | 141.88 (110.28 to 176.09) | 2410.53 (2063.58 to 2791.06) | 4054.22 (3469.93 to 4692.56) | 1039.18 (889.94 to 1202.27) | 1035.83 (887.33 to 1197.77) | 97.23 (51.27 to 166.18) | 162.92 (84.93 to 283.38) | 42.17 (21.96 to 72.01) | 41.63 (22.1 to 72.21) | |
| Japan | 50802.58 (42728.17 to 59912.86) | 100613.39 (84432.57 to 118384.96) | 231 (194.15 to 272.61) | 142.89 (111.18 to 177.58) | 464121.14 (419797.13 to 511647.32) | 968046.49 (875545.25 to 1065757.53) | 1042.35 (892.48 to 1206.45) | 1044.16 (894.02 to 1208.86) | 18765.65 (10010.35 to 31068.99) | 38832.09 (20760.05 to 64776.46) | 42.23 (22.16 to 72.46) | 42 (21.69 to 71.25) | |
| Jordan | 98.98 (74.84 to 126.05) | 573.66 (433.92 to 730.11) | 70.11 (53.11 to 89.24) | 143.06 (111.36 to 177.83) | 641.64 (524.7 to 773.77) | 3716.83 (3040.92 to 4481.44) | 1038.3 (889.26 to 1201.04) | 1045.64 (895.22 to 1210.89) | 26.64 (13.48 to 45.92) | 152.55 (78.63 to 267.74) | 41.21 (21.96 to 70.1) | 41.11 (21.69 to 71.02) | |
| Kazakhstan | 2624.22 (1982.92 to 3345.33) | 3774.72 (2853.65 to 4803.95) | 169.39 (128.08 to 216.12) | 142.64 (110.93 to 177.23) | 25132.62 (21660.55 to 28995.46) | 35563.63 (30640.64 to 41046) | 950.68 (834.4 to 1080.24) | 1042.07 (892.25 to 1206.08) | 1006.19 (535.09 to 1731.27) | 1428.55 (747.3 to 2446.99) | 42.05 (22.2 to 71.05) | 41.82 (22.06 to 71.48) | |
| Kenya | 593.26 (509.97 to 689.97) | 1663.34 (1422.11 to 1932.22) | 62.4 (53.56 to 72.59) | 133.98 (108.93 to 163.74) | 3749.91 (3390.35 to 4165.96) | 10447.67 (9416.96 to 11581.52) | 1056.55 (904.56 to 1224.6) | 950.14 (833.96 to 1079.59) | 155.33 (82 to 268.61) | 433.13 (228.44 to 749.43) | 38.06 (19.91 to 65.25) | 37.78 (19.44 to 64.94) | |
| Kiribati | 4.43 (3.46 to 5.52) | 8.81 (6.86 to 10.97) | 106.62 (83.31 to 132.91) | 142.62 (110.81 to 177.24) | 29.53 (25.27 to 34.16) | 58.6 (50.2 to 67.76) | 1052.4 (900.8 to 1219.49) | 1043.56 (893.74 to 1207.83) | 1.19 (0.63 to 2.1) | 2.37 (1.25 to 4.09) | 42.6 (22.55 to 73.18) | 41.77 (22.05 to 72.25) | |
| Kuwait | 43.49 (32.64 to 55.98) | 212.39 (159.18 to 275.23) | 74.05 (55.65 to 95.22) | 142.95 (110.96 to 177.67) | 289.62 (233.45 to 351.86) | 1417.92 (1142.63 to 1728.4) | 962.74 (831.85 to 1106.12) | 1047.42 (896.55 to 1212.31) | 12.04 (6.22 to 21.1) | 58.37 (29.47 to 103.62) | 42.53 (22.09 to 73.04) | 41.67 (22.18 to 72.15) | |
| Kyrgyzstan | 627.12 (474.27 to 799.07) | 957.56 (723.81 to 1218.14) | 168.87 (127.76 to 215.31) | 135.19 (107.5 to 165.09) | 5972.78 (5147.61 to 6891.68) | 8976.4 (7737.54 to 10354.04) | 882.49 (783.49 to 988.62) | 965.22 (833.97 to 1109.01) | 240.83 (128.33 to 404.65) | 363.64 (189.01 to 619.44) | 38.74 (20.29 to 65.46) | 38.47 (20.06 to 64.93) | |
| Lao People's Democratic Republic | 245.31 (193.08 to 303.83) | 533.31 (419.41 to 660.3) | 101.83 (80.14 to 126.05) | 128.56 (106.72 to 153.07) | 1610.93 (1379.52 to 1862.05) | 3514.94 (3011.81 to 4061.92) | 1044.85 (894.62 to 1209.66) | 887.81 (787.94 to 994.59) | 65.8 (33.86 to 115.64) | 143.78 (74.74 to 245.61) | 35.41 (18.17 to 59.68) | 35.55 (18.54 to 60.73) | |
| Latvia | 257.8 (221.9 to 296.71) | 279.23 (240.47 to 320.42) | 54.88 (47.2 to 63.15) | 142.57 (110.85 to 177.14) | 1375.88 (1237.29 to 1528.36) | 1500.62 (1348.94 to 1668.7) | 1050.08 (899.11 to 1215.99) | 1041.94 (892.28 to 1205.77) | 56.65 (27.85 to 102.35) | 61.91 (31.09 to 111.94) | 42.2 (22.26 to 72.17) | 41.89 (21.85 to 72.83) | |
| Lebanon | 190.49 (147.17 to 238.36) | 527.14 (408.06 to 659.59) | 71.72 (55.51 to 89.74) | 142.7 (110.97 to 177.34) | 1249.24 (1069.78 to 1450.77) | 3487.71 (2985.26 to 4047.01) | 1042.38 (892.73 to 1206.47) | 1043.14 (893.11 to 1207.41) | 51.69 (26.67 to 92.15) | 141.93 (71.86 to 251.14) | 42.08 (21.73 to 72.6) | 41.43 (21.92 to 70.48) | |
| Lesotho | 67.89 (53.09 to 83.91) | 87.41 (68.33 to 107.97) | 66.77 (52.18 to 82.61) | 142.67 (111.04 to 177.22) | 437.8 (373.89 to 506.02) | 560 (478.08 to 646.99) | 1040.39 (891.03 to 1203.72) | 1041.8 (892.17 to 1205.54) | 18.18 (9.36 to 31.69) | 22.96 (11.98 to 40.96) | 41.65 (21.84 to 71.64) | 41.31 (21.78 to 69.73) | |
| Liberia | 98.31 (77.36 to 121.86) | 152.02 (119.62 to 188.36) | 69.03 (54.28 to 85.63) | 143.19 (111.44 to 178.06) | 656.45 (564.44 to 756.97) | 1015.81 (873.99 to 1170.16) | 1050.01 (898.83 to 1216.37) | 1046.79 (895.95 to 1212.46) | 26.92 (13.86 to 47.6) | 41.79 (21.33 to 73.8) | 42.04 (22.22 to 70.85) | 41.57 (21.42 to 71.58) | |
| Libya | 154.77 (119.65 to 193.68) | 396.02 (305.83 to 495.77) | 71.33 (55.24 to 89.23) | 141.93 (110.27 to 176.17) | 1016.51 (870.17 to 1180.41) | 2600.02 (2225.21 to 3019.77) | 881.91 (783.06 to 988.17) | 1036.7 (888.27 to 1199.13) | 42.23 (22.22 to 74.99) | 106.75 (54.88 to 190.61) | 42.16 (22.21 to 72.88) | 41.27 (22.26 to 71.33) | |
| Lithuania | 588.81 (501.82 to 689.11) | 756.74 (644.25 to 885.56) | 100.95 (85.97 to 118.21) | 128.25 (106.48 to 152.69) | 4092.97 (3681.33 to 4550.1) | 5250.7 (4720.73 to 5835.35) | 885.19 (785.71 to 991.64) | 885.05 (785.59 to 991.51) | 165.02 (85.14 to 284.01) | 211.24 (110.25 to 362.8) | 35.32 (18.11 to 60.41) | 35.14 (18.64 to 60.5) | |
| Luxembourg | 142.59 (112.59 to 177.54) | 260.97 (205.69 to 324.72) | 198.21 (156.44 to 246.87) | 129.03 (107.09 to 153.74) | 1159.68 (1004.7 to 1325.91) | 2140.2 (1857.24 to 2445.67) | 880.26 (781.66 to 986.33) | 891.61 (790.78 to 998.75) | 46.5 (24.18 to 79.46) | 85.73 (45.36 to 146.76) | 35.51 (18.72 to 61.81) | 35.86 (18.55 to 61.76) | |
| Madagascar | 405.8 (321.79 to 496.18) | 830.36 (658.46 to 1015.49) | 68.06 (53.89 to 83.34) | 128.21 (106.46 to 152.64) | 2682.06 (2306.8 to 3092.92) | 5461.12 (4696.29 to 6295.95) | 882.05 (783.13 to 988.17) | 884.81 (785.37 to 991.27) | 111.19 (56.93 to 193.85) | 227.57 (115.69 to 395.74) | 35.02 (18.13 to 59.9) | 34.86 (18.24 to 60.03) | |
| Malawi | 311.94 (247.49 to 381.42) | 579.73 (459.8 to 709.08) | 68.62 (54.32 to 84.08) | 128.37 (106.58 to 152.83) | 2058.22 (1769.82 to 2373.77) | 3836.61 (3299.64 to 4425.93) | 1043.85 (893.66 to 1208.28) | 886.24 (786.57 to 992.86) | 85.78 (44 to 151.85) | 159.25 (82.12 to 280.54) | 35.48 (18.6 to 60.39) | 35.38 (17.89 to 61.26) | |
| Malaysia | 1067.89 (839.42 to 1321.73) | 3585.87 (2821.68 to 4438.73) | 101.63 (79.97 to 125.75) | 142.74 (111.01 to 177.39) | 7076.64 (6064.04 to 8180.06) | 23658.35 (20260.44 to 27352.2) | 1099.91 (949.95 to 1261.01) | 1043.37 (893.36 to 1207.72) | 287.18 (146.43 to 510.2) | 957.79 (495.74 to 1686.46) | 41.9 (22.09 to 72.03) | 41.29 (21.82 to 70.75) | |
| Maldives | 9.67 (7.61 to 12) | 35.76 (28.11 to 44.25) | 97.8 (77.09 to 121.28) | 144.9 (115.8 to 175.39) | 62.87 (53.8 to 72.59) | 236.36 (202.33 to 273.11) | 714.68 (650.84 to 785.42) | 1103.37 (954.8 to 1266.78) | 2.59 (1.33 to 4.49) | 9.69 (4.91 to 17.06) | 44.23 (22.69 to 74.77) | 44.06 (23.23 to 75.07) | |
| Mali | 348.59 (265.32 to 442.99) | 753.1 (575.26 to 959.56) | 74.57 (56.78 to 94.87) | 112.44 (98.25 to 128.47) | 2398.64 (1991.18 to 2864.38) | 5188.32 (4290.9 to 6227.4) | 1427.46 (1300.87 to 1565.45) | 706.92 (642.99 to 777.33) | 98.71 (50.78 to 174.46) | 214.66 (111.36 to 380.6) | 28.83 (15.31 to 49.39) | 28.65 (15.11 to 48.95) | |
| Malta | 109.75 (86.74 to 136.53) | 262.53 (207.02 to 326.58) | 198.11 (156.34 to 246.64) | 169.41 (147.47 to 194.22) | 874.99 (759.25 to 999.57) | 2135.85 (1851.91 to 2442.05) | 879.28 (780.82 to 985.24) | 1428.63 (1302.12 to 1566.88) | 35.26 (18.29 to 59.59) | 85.69 (44.41 to 143.37) | 56.27 (30.15 to 92.61) | 56.33 (29.98 to 93.05) | |
| Marshall Islands | 1.97 (1.54 to 2.46) | 4.11 (3.2 to 5.12) | 105.88 (82.78 to 132.03) | 127.91 (106.12 to 152.24) | 13.15 (11.25 to 15.21) | 27.16 (23.27 to 31.4) | 885.67 (786.2 to 992.23) | 882.41 (783.46 to 988.6) | 0.53 (0.28 to 0.95) | 1.1 (0.58 to 1.93) | 35.32 (18.34 to 61.37) | 35.36 (18.75 to 61.36) | |
| Mauritania | 82.25 (64.69 to 101.81) | 179.27 (141.08 to 222.17) | 69.93 (54.96 to 86.62) | 128.57 (106.73 to 153.09) | 550.78 (473.06 to 634.89) | 1200.11 (1031.95 to 1382.97) | 1466.85 (1309.03 to 1637.35) | 888.02 (788.09 to 994.88) | 22.93 (11.87 to 40.77) | 49.9 (25.48 to 87.75) | 35.38 (18.47 to 60.32) | 35.29 (18.21 to 61.28) | |
| Mauritius | 91.68 (72.1 to 113.6) | 251.08 (197.42 to 311.17) | 102.84 (80.88 to 127.33) | 170.06 (138.1 to 204.57) | 605.48 (518.87 to 699.61) | 1664.47 (1426.05 to 1924.24) | 470.67 (402.87 to 546.22) | 1466.67 (1308.89 to 1637.17) | 24.65 (12.7 to 42.94) | 67.02 (35.04 to 117.83) | 58.84 (31.21 to 99.19) | 58.55 (31.26 to 98.95) | |
| Mexico | 5580.83 (4882.61 to 6367.97) | 17433.14 (15238.72 to 19919.17) | 112.92 (98.68 to 128.89) | 71 (55.07 to 88.87) | 35054.25 (31927.48 to 38535.65) | 109133.15 (99270.57 to 120037.17) | 463.22 (373.43 to 565.56) | 467.96 (400.51 to 543.09) | 1419.93 (754.97 to 2429.89) | 4433.97 (2339.07 to 7569.28) | 19.39 (9.93 to 34.68) | 18.99 (9.9 to 33.99) | |
| Micronesia (Federated States of) | 6.29 (4.92 to 7.85) | 9.34 (7.28 to 11.64) | 105.7 (82.64 to 131.86) | 70.83 (53.13 to 91.44) | 42.02 (35.99 to 48.62) | 62.02 (53.16 to 71.67) | 268.16 (224.75 to 318.31) | 462.42 (372.54 to 564.36) | 1.71 (0.88 to 3.01) | 2.52 (1.31 to 4.37) | 19.14 (9.63 to 33.83) | 18.98 (9.68 to 33.59) | |
| Monaco | 18.5 (14.58 to 23.04) | 24.95 (19.65 to 31.08) | 197.82 (156.17 to 246.02) | 46.45 (36.27 to 58.07) | 153.65 (133.3 to 175.67) | 206.57 (179.11 to 236.25) | 457.18 (373.77 to 551.96) | 266.43 (222.78 to 316.48) | 6.2 (3.25 to 10.51) | 8.29 (4.33 to 14.03) | 11.12 (5.51 to 19.96) | 10.93 (5.55 to 19.58) | |
| Mongolia | 204.63 (155.16 to 260.07) | 422.77 (319.51 to 537.66) | 167.25 (126.79 to 212.53) | 69.95 (53 to 89.04) | 1903.49 (1640.4 to 2199.36) | 3972.1 (3424.75 to 4580.74) | 534.5 (474.93 to 599.02) | 456.15 (373.19 to 550.28) | 76.69 (40.9 to 129.75) | 160.52 (84.53 to 272.61) | 18.88 (9.54 to 32.88) | 18.64 (9.59 to 32.82) | |
| Montenegro | 67.03 (52.7 to 82.37) | 115.48 (90.94 to 141.67) | 86.78 (68.31 to 106.61) | 79 (66.25 to 93.55) | 428.06 (368.41 to 494.55) | 733.24 (630.79 to 846.09) | 473.06 (404.9 to 549.17) | 535.92 (475.9 to 600.59) | 17.78 (9.19 to 31.44) | 30.34 (15.52 to 53.76) | 22.06 (11.6 to 38.18) | 22.02 (11.7 to 38.62) | |
| Morocco | 1220.55 (944.05 to 1527.56) | 3041.16 (2352.1 to 3804.33) | 71.43 (55.3 to 89.41) | 71.79 (55.55 to 89.85) | 8014.76 (6860.66 to 9307.71) | 19957.5 (17087.65 to 23167.49) | 472.91 (404.83 to 548.97) | 473.47 (405.28 to 549.52) | 333.84 (173.43 to 593.66) | 817.49 (422.78 to 1464.8) | 19.49 (9.9 to 34.86) | 19.25 (9.91 to 34.04) | |
| Mozambique | 469.22 (372.17 to 573.9) | 852.99 (676.5 to 1043.62) | 68.47 (54.21 to 83.91) | 72.03 (55.69 to 90.12) | 3100.49 (2666.17 to 3575.23) | 5634.98 (4846.21 to 6499.49) | 461.72 (395.06 to 536.21) | 475.04 (406.72 to 551.4) | 127.42 (66.02 to 223.83) | 232.48 (118.87 to 400.17) | 19.46 (10.04 to 34.75) | 19.37 (9.82 to 34.21) | |
| Myanmar | 2793.82 (2197.32 to 3460.26) | 6083.45 (4785.38 to 7534.94) | 102.15 (80.38 to 126.43) | 70.27 (54.49 to 87.86) | 18393.69 (15762.68 to 21258.26) | 40312.83 (34556.99 to 46591.08) | 496.96 (400.79 to 604.69) | 462.7 (395.94 to 537) | 749.29 (395.43 to 1318.48) | 1630.15 (851.97 to 2834.73) | 18.93 (9.82 to 33.82) | 18.85 (9.63 to 34.1) | |
| Namibia | 52.59 (41.11 to 64.89) | 105.22 (82.26 to 129.9) | 65.98 (51.58 to 81.49) | 74.21 (55.7 to 96.11) | 335.68 (286.67 to 387.48) | 675.37 (576.79 to 779.91) | 469.83 (402.1 to 545.49) | 497.79 (401.58 to 607.58) | 14.02 (7.16 to 25.01) | 27.98 (14.36 to 49.46) | 20.54 (10.55 to 36.21) | 20.39 (10.26 to 36.27) | |
| Nauru | 0.53 (0.41 to 0.66) | 0.68 (0.53 to 0.85) | 104.06 (81.23 to 129.58) | 71.7 (55.48 to 89.71) | 3.48 (2.98 to 4.03) | 4.56 (3.91 to 5.28) | 766.45 (642.04 to 906.94) | 472.8 (404.73 to 548.69) | 0.14 (0.07 to 0.25) | 0.18 (0.1 to 0.32) | 19.44 (9.84 to 34.32) | 19.45 (9.99 to 34.57) | |
| Nepal | 773.75 (605.91 to 955.12) | 2112.22 (1655.67 to 2608.51) | 73.05 (57.25 to 90.18) | 100.51 (75.24 to 128.85) | 4959.87 (4221.11 to 5768.61) | 13597.49 (11578.97 to 15827) | 493.28 (406.01 to 589.97) | 766.37 (642.07 to 911.22) | 203.58 (103.86 to 357.55) | 557.26 (289.04 to 1006.42) | 31.2 (16 to 53.59) | 30.93 (15.94 to 53.08) | |
| Netherlands | 5170.85 (4082.03 to 6436.55) | 9185.13 (7243.18 to 11424.61) | 197.94 (156.25 to 246.35) | 73.9 (56.62 to 93.43) | 41996.79 (36411.21 to 48002.8) | 74766.08 (64855.39 to 85454.62) | 466.17 (399 to 541.29) | 491.35 (405.13 to 587.94) | 1688.99 (882.5 to 2901.13) | 2989.66 (1554.46 to 5038.84) | 20.29 (10.65 to 35.92) | 20.11 (10.3 to 35.32) | |
| New Zealand | 976.88 (825.16 to 1146.44) | 2072.8 (1760.55 to 2418.11) | 187.11 (157.98 to 219.78) | 70.33 (54.56 to 88.04) | 7722.83 (6977.17 to 8551.99) | 16513.97 (14830.97 to 18241.75) | 453.68 (369.34 to 548.26) | 463.1 (396.25 to 538.15) | 306.65 (162.87 to 523.97) | 658.8 (345.83 to 1104.11) | 19.04 (9.68 to 33.85) | 18.9 (9.73 to 33.9) | |
| Nicaragua | 224.17 (186.28 to 266.61) | 745.01 (619.43 to 886.22) | 128.24 (106.4 to 152.68) | 69.77 (52.55 to 89.41) | 1537.61 (1365.12 to 1722.48) | 5124.07 (4548.19 to 5739.98) | 470.87 (403.03 to 546.67) | 454.46 (370.25 to 548.82) | 61.64 (32.19 to 104.98) | 204.19 (105.34 to 355.24) | 18.58 (9.46 to 33.65) | 18.59 (9.55 to 33.04) | |
| Niger | 217.89 (171.51 to 270.01) | 661.52 (520.55 to 819.41) | 69.28 (54.47 to 85.9) | 71.56 (55.41 to 89.53) | 1449.4 (1247.47 to 1669.94) | 4408.97 (3791.05 to 5081.1) | 475.16 (406.67 to 551.57) | 471.76 (403.84 to 547.45) | 60.27 (31.64 to 104.24) | 183.26 (93.88 to 321.32) | 19.57 (10.14 to 34.87) | 19.26 (9.94 to 34.52) | |
| Nigeria | 3672.19 (3137.46 to 4278.92) | 7075.33 (6031.24 to 8235.75) | 70.15 (59.88 to 81.73) | 71.65 (55.44 to 89.67) | 24361.88 (21998.03 to 27039.33) | 46947.12 (42362.33 to 52009.84) | 470.08 (402.38 to 545.65) | 472.43 (404.34 to 548.37) | 1004.82 (524.68 to 1730.9) | 1947.3 (1021.24 to 3342.13) | 19.54 (9.85 to 34.74) | 19.42 (10.01 to 35.03) | |
| Niue | 0.3 (0.23 to 0.37) | 0.3 (0.23 to 0.37) | 106.45 (83.24 to 132.8) | 71.56 (55.36 to 89.57) | 2.02 (1.73 to 2.34) | 2.02 (1.73 to 2.33) | 467.01 (399.58 to 542.23) | 471.69 (403.7 to 547.59) | 0.08 (0.04 to 0.14) | 0.08 (0.04 to 0.14) | 19.48 (10.2 to 34.63) | 19.31 (9.91 to 34.58) | |
| North Macedonia | 198.31 (155.94 to 243.61) | 383.82 (302.14 to 470.82) | 86.01 (67.76 to 105.57) | 68.46 (53.01 to 85.6) | 1257.77 (1081.43 to 1452.16) | 2422.84 (2083.13 to 2795.02) | 468.74 (401.16 to 544.29) | 449.42 (383.9 to 523.03) | 51.96 (26.78 to 93.74) | 99.63 (51.2 to 176.83) | 19.2 (10.08 to 34.22) | 18.44 (9.23 to 32.84) | |
| Northern Mariana Islands | 1.54 (1.2 to 1.92) | 6.47 (5.05 to 8.06) | 104.38 (81.62 to 130.04) | 71.32 (55.19 to 89.27) | 10.18 (8.73 to 11.8) | 42.97 (36.85 to 49.74) | 476.42 (405.6 to 554.05) | 469.8 (402.02 to 545.42) | 0.42 (0.21 to 0.72) | 1.75 (0.9 to 3) | 19.42 (9.87 to 34.39) | 19.31 (9.95 to 34.3) | |
| Norway | 1953.46 (1640.39 to 2303.82) | 2751.07 (2312.26 to 3239.76) | 213.24 (179.09 to 251.67) | 73.15 (57.33 to 90.31) | 16467.67 (14833.73 to 18322.52) | 23198.7 (20847.12 to 25693.67) | 475.08 (429.03 to 526.06) | 474.46 (404.22 to 551.6) | 652.74 (345.93 to 1084.55) | 925.49 (489.96 to 1550.66) | 19.7 (10.32 to 35.04) | 19.58 (9.86 to 34.69) | |
| Oman | 49.63 (37.6 to 63.71) | 138.55 (104.22 to 177.71) | 69.67 (52.76 to 89.49) | 74.1 (63.4 to 86.14) | 321.16 (261.31 to 387.87) | 895.03 (728.72 to 1078.75) | 467.84 (400.3 to 543.35) | 478.43 (431.97 to 529.57) | 13.21 (6.74 to 23.87) | 36.85 (19.02 to 65) | 19.26 (10.04 to 33.7) | 19.49 (10.14 to 34.16) | |
| Pakistan | 4829.55 (4139.98 to 5606.5) | 9945.56 (8518.02 to 11564.51) | 72.81 (62.37 to 84.55) | 72.25 (55.84 to 90.4) | 30895.41 (27901.56 to 34263.43) | 63427.33 (57290.44 to 70282.93) | 469.44 (399.83 to 545.45) | 476.49 (407.59 to 553.63) | 1272.54 (659.61 to 2224.69) | 2607.97 (1360.57 to 4552.94) | 19.15 (9.86 to 34.45) | 19.18 (9.86 to 34.64) | |
| Palau | 1.27 (0.99 to 1.58) | 2.93 (2.29 to 3.65) | 105.54 (82.5 to 131.59) | 72.91 (57.14 to 90) | 8.47 (7.26 to 9.8) | 19.46 (16.68 to 22.5) | 468.62 (423.22 to 519.64) | 472.67 (402.63 to 549.3) | 0.34 (0.18 to 0.6) | 0.78 (0.4 to 1.38) | 19.25 (9.97 to 34) | 19.47 (10.06 to 34.87) | |
| Palestine | 77.41 (58.83 to 98.55) | 208.74 (160.1 to 263.3) | 74.13 (56.38 to 94.36) | 73.35 (62.77 to 85.2) | 512.63 (422.04 to 612.82) | 1377.8 (1135.97 to 1647.12) | 380.7 (326.18 to 440.27) | 472.48 (426.66 to 523.5) | 21.14 (11.11 to 37.36) | 56.65 (29.04 to 98.88) | 19.25 (9.96 to 33.71) | 19.32 (10.06 to 33.86) | |
| Panama | 226.32 (187.78 to 269.21) | 698.72 (579.18 to 832.35) | 127.53 (105.76 to 151.78) | 60.6 (47.65 to 74.62) | 1553.24 (1379.42 to 1740.3) | 4830.24 (4287.43 to 5412.94) | 473.77 (403.56 to 550.7) | 384.17 (329.42 to 444.37) | 62.48 (32.44 to 108.67) | 193.43 (102.57 to 335.59) | 15.63 (7.93 to 27.75) | 15.81 (8.02 to 28.06) | |
| Papua New Guinea | 211.49 (164.92 to 263.74) | 551.52 (430.01 to 687.21) | 105.03 (82.06 to 130.9) | 73.5 (57.62 to 90.8) | 1398.55 (1198.99 to 1617.59) | 3657.79 (3137.42 to 4234.11) | 383.25 (328.44 to 443.2) | 476.95 (406.45 to 554.82) | 56.96 (28.92 to 99.56) | 148.99 (78.81 to 262.78) | 19.34 (9.82 to 34.06) | 19.49 (10.1 to 35.18) | |
| Paraguay | 450.28 (365.75 to 541.62) | 1211.88 (983.75 to 1458.96) | 170.1 (138.17 to 204.6) | 60.81 (47.81 to 74.88) | 3856.82 (3441.3 to 4306.92) | 10383.44 (9263.29 to 11598.87) | 383.89 (329.15 to 444.04) | 385.62 (330.44 to 446.07) | 155.06 (82.29 to 261.43) | 415.61 (221.78 to 702.53) | 15.66 (8.06 to 27.79) | 15.78 (7.96 to 27.75) | |
| Peru | 1834.47 (1472.58 to 2226.5) | 5372.58 (4308.32 to 6529.35) | 132.21 (106.08 to 160.57) | 60.78 (47.78 to 74.77) | 12871.93 (11302.88 to 14590.78) | 37869.69 (33225.83 to 42966.54) | 294.47 (248.57 to 347.9) | 385.29 (330.42 to 445.59) | 521.07 (273.48 to 890.09) | 1526.62 (801.97 to 2579.8) | 15.64 (7.98 to 27.92) | 15.8 (7.98 to 27.9) | |
| Philippines | 3389.21 (2909.18 to 3927.93) | 10004.42 (8581.58 to 11611.62) | 102.23 (87.66 to 118.47) | 49.34 (38.94 to 61.44) | 22208.77 (20062.17 to 24576.36) | 65687.43 (59328.51 to 72735.02) | 381.56 (327.11 to 441.46) | 292.47 (246.82 to 344.9) | 901.07 (482.96 to 1536.03) | 2663.13 (1416.58 to 4444.53) | 12.1 (6.09 to 21.8) | 12.06 (6.03 to 21.82) | |
| Poland | 5031.79 (4335.74 to 5826.75) | 8513.07 (7330.42 to 9845.54) | 87.55 (75.38 to 101.37) | 60.67 (47.71 to 74.72) | 32104.26 (28868.05 to 35588.29) | 54182.29 (48766.67 to 60039.46) | 508.35 (425.63 to 600.3) | 384.69 (329.63 to 444.92) | 1317.24 (686.91 to 2314.35) | 2230.45 (1170.53 to 3888.32) | 15.59 (7.97 to 27.35) | 15.79 (8.02 to 27.87) | |
| Portugal | 3758.71 (2972.03 to 4674.37) | 6216.79 (4891.97 to 7750.53) | 198.09 (156.34 to 246.6) | 74.2 (57.32 to 93.43) | 29941.88 (25966.07 to 34216.81) | 52059.82 (45134.52 to 59520.16) | 456.39 (392.54 to 526.41) | 506.84 (425.91 to 598.56) | 1197.58 (628.57 to 2016.4) | 2069.96 (1093.33 to 3466.23) | 20.93 (11 to 36.63) | 20.77 (10.75 to 37.12) | |
| Puer to Rico | 668.54 (520.91 to 830.14) | 1335.5 (1039.11 to 1663.86) | 142.95 (111.26 to 177.62) | 67.83 (53.71 to 83.19) | 4865.7 (4166.98 to 5632.86) | 9903 (8472.42 to 11471.65) | 456.92 (393.04 to 527.01) | 453.29 (389.99 to 523.14) | 196.58 (101.75 to 338.53) | 393.78 (206.08 to 673.47) | 18.82 (9.6 to 33.94) | 18.8 (9.72 to 33.37) | |
| Qatar | 6.06 (4.68 to 7.57) | 55.93 (43.11 to 69.89) | 70.13 (54.39 to 87.65) | 68.77 (54.46 to 84.25) | 39.51 (33.82 to 45.9) | 363.36 (311.04 to 422.02) | 459.52 (395.3 to 530.14) | 459.86 (395.7 to 530.59) | 1.63 (0.85 to 2.9) | 14.97 (7.67 to 27.02) | 18.88 (10.01 to 33.79) | 19 (9.67 to 33.68) | |
| Republic of Korea | 7031.31 (5360.31 to 8909.87) | 25920.77 (19895.41 to 32756.01) | 207.79 (158.24 to 263.51) | 67.99 (53.85 to 83.29) | 59577.62 (50653.35 to 69530.55) | 223924.26 (190008.75 to 260871.56) | 397.48 (359.28 to 441.38) | 454.31 (390.93 to 524.19) | 2398.61 (1256.85 to 4106.45) | 8995 (4740.82 to 15329.04) | 18.99 (9.96 to 33.54) | 18.91 (9.72 to 33.44) | |
| Republic of Moldova | 565.9 (483.28 to 661.65) | 805.27 (686.83 to 942.22) | 100.24 (85.44 to 117.3) | 62.9 (53.73 to 73.09) | 3902.36 (3510.2 to 4340.42) | 5560.03 (5000.83 to 6182.39) | 458.76 (394.69 to 529.27) | 400.12 (360.61 to 443.43) | 157.59 (79.67 to 271.44) | 225.02 (115.61 to 393.18) | 16.39 (8.64 to 28.41) | 16.49 (8.66 to 28.61) | |
| Romania | 2331.94 (1847.64 to 2875.81) | 3204.57 (2543.88 to 3942.94) | 64.02 (50.7 to 78.93) | 69.01 (54.62 to 84.58) | 13290.55 (11314.15 to 15533.05) | 18324.61 (15653.71 to 21427.39) | 454.67 (391.23 to 524.45) | 461.76 (397.21 to 532.8) | 550.71 (280.64 to 978.95) | 757.42 (380.48 to 1332.68) | 18.99 (9.71 to 33.73) | 19.08 (9.79 to 33.72) | |
| Russian Federation | 30664.73 (26831.2 to 34876.62) | 43211 (37898.15 to 49210.5) | 129.72 (113.46 to 147.5) | 68.43 (54.18 to 83.84) | 266434.61 (245418.9 to 289309.31) | 379030.11 (349735.86 to 411349.49) | 686.76 (588.36 to 794.02) | 457.49 (393.5 to 527.63) | 10641.77 (5702.21 to 17671.61) | 15066.29 (8079.3 to 24791.31) | 18.76 (9.59 to 32.81) | 18.93 (9.6 to 33.08) | |
| Rwanda | 168.73 (132.92 to 208.09) | 379.46 (298.6 to 468.39) | 50.69 (39.89 to 62.64) | 102.43 (80.59 to 126.8) | 976.81 (827.63 to 1142.09) | 2199.15 (1861.65 to 2572.73) | 457.79 (393.79 to 528.03) | 683.31 (585.46 to 790.26) | 40.68 (20.56 to 72.42) | 92.05 (46.37 to 166.65) | 27.78 (14.29 to 48.56) | 27.39 (14.31 to 48.12) | |
| Saint Kitts and Nevis | 7.69 (6 to 9.54) | 12.87 (9.99 to 15.99) | 143.51 (111.73 to 178.45) | 68.96 (54.58 to 84.55) | 55.67 (47.65 to 64.54) | 92.23 (78.99 to 106.78) | 408.11 (367.66 to 453.83) | 461.45 (396.92 to 532.41) | 2.23 (1.16 to 3.82) | 3.7 (1.98 to 6.34) | 18.71 (9.69 to 32.93) | 18.93 (9.63 to 32.79) | |
| Saint Lucia | 16.4 (12.79 to 20.36) | 43.09 (33.5 to 53.52) | 143.5 (111.75 to 178.41) | 63.7 (54.46 to 74.19) | 118.71 (101.64 to 137.5) | 314.22 (269.05 to 363.7) | 464.96 (399.87 to 536.87) | 409.32 (368.76 to 454.8) | 4.77 (2.46 to 8.22) | 12.49 (6.61 to 21.24) | 16.7 (8.81 to 29) | 16.83 (8.84 to 29.5) | |
| Saint Vincent and the Grenadines | 13.41 (10.46 to 16.67) | 26.48 (20.58 to 32.84) | 143.52 (111.75 to 178.51) | 69.51 (55.02 to 85.27) | 97.31 (83.29 to 112.75) | 192.08 (164.62 to 222.14) | 452.58 (389.43 to 521.95) | 465.36 (400.18 to 537.28) | 3.91 (2.06 to 6.75) | 7.66 (4.14 to 13.26) | 19.15 (9.82 to 33.85) | 19.12 (9.77 to 34.11) | |
| Samoa | 10.79 (8.42 to 13.46) | 18.06 (14.11 to 22.51) | 105.61 (82.55 to 131.8) | 68.66 (54.37 to 84.15) | 72.02 (61.72 to 83.33) | 120.96 (103.66 to 139.93) | 458.58 (394.54 to 529.03) | 459.13 (394.96 to 529.6) | 2.93 (1.55 to 5.04) | 4.89 (2.52 to 8.56) | 18.68 (9.73 to 33.21) | 18.9 (9.78 to 33.04) | |
| San Marino | 9.19 (7.25 to 11.43) | 18 (14.15 to 22.47) | 197.73 (156.08 to 245.94) | 69.33 (54.89 to 85.01) | 74.5 (64.62 to 85.17) | 151.6 (131.51 to 173.3) | 687.72 (589.28 to 795.18) | 464 (399.04 to 535.56) | 2.99 (1.62 to 5.19) | 6.04 (3.2 to 10.24) | 18.98 (9.64 to 33.49) | 19.15 (9.92 to 33.84) | |
| Sao tome and Principe | 5.73 (4.51 to 7.1) | 8.72 (6.86 to 10.8) | 70.06 (55.06 to 86.78) | 101.87 (80.15 to 126.11) | 38.47 (33.07 to 44.33) | 58.4 (50.23 to 67.29) | 297.73 (252.18 to 348.39) | 678.77 (581.14 to 785.02) | 1.6 (0.82 to 2.8) | 2.42 (1.22 to 4.33) | 27.92 (14.71 to 49.69) | 27.25 (14.34 to 48.16) | |
| Saudi Arabia | 440.18 (340.84 to 551.12) | 1271.24 (983 to 1591.76) | 70.76 (54.87 to 88.57) | 50.97 (40.09 to 63.03) | 2883.89 (2467.97 to 3351.2) | 8290.46 (7099.32 to 9638.77) | 683.63 (593.6 to 779.47) | 299.48 (253.43 to 350.64) | 118.31 (60.15 to 210.23) | 341.39 (176.68 to 609.75) | 12.32 (6.19 to 21.98) | 12.46 (6.24 to 22.56) | |
| Senegal | 265.33 (208.89 to 328.82) | 634.12 (498.8 to 785.33) | 69.4 (54.58 to 86.06) | 87.55 (68.51 to 108.45) | 1773.57 (1525.11 to 2043.97) | 4244.81 (3650.03 to 4891.36) | 427.86 (365.67 to 494.19) | 668.45 (578.93 to 768.11) | 73.55 (38.59 to 129.53) | 175.91 (90.5 to 313.89) | 27.76 (14.59 to 48.55) | 27.47 (14.21 to 48.43) | |
| Serbia | 1285.78 (1010.44 to 1577.62) | 1940.65 (1528.89 to 2383.37) | 86.28 (67.94 to 105.89) | 66.14 (51.69 to 81.72) | 8167.12 (7023.98 to 9431.07) | 12339.29 (10610.5 to 14236.95) | 632.76 (526.83 to 748.61) | 429.31 (366.81 to 495.84) | 338.37 (178 to 599.94) | 506.71 (264 to 895.59) | 17.74 (9.02 to 31.55) | 17.61 (8.92 to 31.13) | |
| Seychelles | 7.47 (5.88 to 9.25) | 14.53 (11.42 to 18) | 102.96 (80.99 to 127.49) | 85.28 (64.87 to 108.13) | 49.83 (42.7 to 57.63) | 96.16 (82.36 to 111.12) | 428.16 (365.93 to 494.57) | 639.49 (532.07 to 756.75) | 2.02 (1.07 to 3.6) | 3.88 (2.05 to 6.89) | 26.06 (13.36 to 45.85) | 26.45 (13.59 to 46.43) | |
| Sierra Leone | 171.11 (134.68 to 212.08) | 294.96 (232.06 to 365.42) | 69.35 (54.55 to 86) | 66.33 (51.85 to 81.99) | 1147.17 (986.37 to 1322.34) | 1975.16 (1698.82 to 2276.05) | 429.41 (387.8 to 475.62) | 430.65 (367.98 to 497.61) | 47.4 (24.17 to 83.52) | 82.01 (42.05 to 142.68) | 17.77 (9.05 to 31.75) | 17.77 (9.11 to 31.48) | |
| Singapore | 448.63 (344.55 to 567.23) | 1949.45 (1500.76 to 2468.96) | 174.32 (133.81 to 220.55) | 66.65 (57.08 to 77.46) | 3361.07 (2780.36 to 4005.01) | 14694.63 (12180.16 to 17479.56) | 433.79 (370.63 to 501.44) | 430.05 (388.38 to 476.33) | 136.51 (69.76 to 233.09) | 598.5 (310.73 to 1037.8) | 17.73 (9.34 to 31.03) | 17.6 (9.4 to 30.79) | |
| Slovakia | 687.08 (540.9 to 844.8) | 1116.55 (879.35 to 1370.7) | 86.91 (68.4 to 106.78) | 66.8 (52.18 to 82.63) | 4390.4 (3777.41 to 5071.39) | 7119.82 (6123.35 to 8218.62) | 429.44 (367.06 to 496.33) | 434.09 (370.87 to 501.81) | 180.17 (92.41 to 318.1) | 292.16 (154.51 to 508.02) | 17.97 (9.24 to 31.39) | 17.72 (9.19 to 31.65) | |
| Slovenia | 277.21 (217.92 to 340.73) | 496.92 (391.77 to 609.86) | 87.76 (68.99 to 107.81) | 66.69 (52.11 to 82.51) | 1784.11 (1535.32 to 2061.76) | 3165.21 (2721.98 to 3649.78) | 424.61 (363.04 to 490.29) | 433.21 (370.22 to 500.71) | 73.1 (38.24 to 127.92) | 129.78 (67.34 to 233.83) | 17.75 (9.12 to 31.42) | 17.76 (9.27 to 31.32) | |
| Solomon Islands | 15.75 (12.28 to 19.61) | 38.28 (29.9 to 47.69) | 104.09 (81.26 to 129.55) | 66.32 (51.87 to 81.96) | 104.05 (89.31 to 120.52) | 254.98 (218.59 to 294.92) | 470.1 (404.14 to 541.78) | 430.33 (367.8 to 497.12) | 4.28 (2.24 to 7.41) | 10.39 (5.4 to 18.3) | 17.58 (9.1 to 31.14) | 17.74 (9.26 to 31.56) | |
| Somalia | 156.89 (124.32 to 191.94) | 461.06 (365.99 to 563.86) | 68.59 (54.31 to 84.04) | 70.07 (55.06 to 86.77) | 1033.69 (889.29 to 1192.17) | 3028.77 (2603.7 to 3494.37) | 474.41 (407.81 to 547.06) | 472.85 (406.51 to 544.88) | 43.11 (21.98 to 75.82) | 126.01 (65.72 to 221.19) | 19.26 (9.79 to 34.08) | 19.53 (10.14 to 34.32) | |
| South Africa | 1634.91 (1402.03 to 1899.2) | 3775.12 (3237.71 to 4384.23) | 66.57 (57.03 to 77.37) | 70.69 (55.53 to 87.51) | 10475.08 (9465.17 to 11600.58) | 24142.22 (21820.21 to 26737.52) | 465.03 (400.09 to 536.16) | 477.44 (410.22 to 550.53) | 433.7 (228.75 to 758.15) | 991.75 (531.12 to 1731.11) | 19.72 (10.11 to 35.1) | 19.81 (10.16 to 34.67) | |
| South Sudan | 208.3 (165.37 to 255.14) | 275.38 (218.33 to 336.97) | 67.16 (53.2 to 82.43) | 69.43 (54.58 to 86.06) | 1379.66 (1186 to 1592.28) | 1817.81 (1564.12 to 2096.55) | 466.08 (400.98 to 537.14) | 467.93 (402.44 to 539.18) | 56.73 (29.2 to 100.36) | 74.53 (37.9 to 130.12) | 19.02 (9.77 to 33.7) | 19.19 (9.76 to 33.9) | |
| Spain | 13832.09 (10461.27 to 17787.46) | 22562.67 (17025.07 to 29081.62) | 187.56 (141.74 to 241.35) | 69.41 (54.59 to 86.05) | 106687.7 (85310.74 to 130188.14) | 181372.07 (144670.36 to 221657.67) | 470.68 (404.7 to 542.42) | 467.99 (402.61 to 539.27) | 4264.58 (2210.37 to 7317.53) | 7219.89 (3691.76 to 12479.83) | 19 (9.9 to 33.08) | 19.29 (9.8 to 33.79) | |
| Sri Lanka | 1269.99 (997.39 to 1577.32) | 3699.12 (2877.37 to 4595.09) | 101.21 (79.46 to 125.81) | 69.95 (54.97 to 86.63) | 8358.74 (7101.67 to 9773.25) | 24532.36 (20809.33 to 28744.86) | 467.77 (402.46 to 539.17) | 471.89 (405.67 to 543.75) | 339.18 (177 to 577.13) | 983.76 (510.81 to 1714.26) | 19.47 (9.88 to 34.33) | 19.51 (9.92 to 34.45) | |
| Sudan | 771.3 (597.54 to 964.97) | 1506.86 (1166.67 to 1885.83) | 71.15 (55.14 to 89.01) | 69.87 (54.91 to 86.57) | 5055.65 (4327.3 to 5872.38) | 9880.18 (8456.42 to 11482.66) | 556.9 (475.81 to 645.81) | 471.4 (405.29 to 543.18) | 209.73 (107.44 to 368.27) | 408.06 (213.13 to 725.34) | 19.41 (9.95 to 34.37) | 19.44 (9.99 to 34.9) | |
| Suriname | 42.93 (33.32 to 53.31) | 112.18 (87.23 to 139.44) | 142.41 (110.71 to 176.89) | 78.17 (60.99 to 97.7) | 311.54 (266.83 to 360.47) | 814.87 (697.46 to 943.79) | 391.17 (333.12 to 454.7) | 558.94 (477.67 to 652.1) | 12.63 (6.69 to 21.27) | 32.44 (16.7 to 55.88) | 23.01 (11.98 to 40.82) | 23.1 (11.82 to 41.13) | |
| Sweden | 3991.02 (3410.67 to 4615.73) | 5476 (4675.93 to 6338.71) | 199.31 (170.38 to 230.67) | 62.09 (48.57 to 77.02) | 32483.41 (29334.27 to 35813.51) | 45038.16 (40700.07 to 49668.49) | 470.17 (404.35 to 542.01) | 394.53 (336.01 to 458.98) | 1299.35 (696.13 to 2180.95) | 1794.11 (957.28 to 3028.41) | 16.24 (8.39 to 28.45) | 16.38 (8.23 to 29.5) | |
| Switzerland | 2650.59 (2089.68 to 3302.42) | 4471.94 (3519.71 to 5571.95) | 198.06 (156.34 to 246.6) | 68.93 (54.18 to 85.51) | 21848.99 (18940.58 to 24980.39) | 37228.39 (32288.94 to 42560.46) | 468.51 (402.78 to 539.92) | 464.35 (399.57 to 535.47) | 868.6 (463.17 to 1460.03) | 1478.24 (785.48 to 2503.63) | 19.41 (9.96 to 34.46) | 19.16 (9.88 to 33.47) | |
| Syrian Arab Republic | 427.6 (330.6 to 535.23) | 1149.12 (887.89 to 1437.89) | 71.14 (55.12 to 89.03) | 69.35 (54.55 to 86) | 2802.98 (2399.63 to 3256.24) | 7522.78 (6440.16 to 8737.89) | 469.3 (403.59 to 540.87) | 467.43 (402.05 to 538.68) | 116.66 (59.34 to 206.26) | 310.49 (160.73 to 548.08) | 19.38 (9.8 to 33.81) | 19.3 (9.83 to 33.69) | |
| Taiwan (Province of China) | 2985.93 (2274.14 to 3753.6) | 8548.52 (6477.89 to 10830.8) | 146.97 (111.99 to 184.85) | 70.19 (55.16 to 86.9) | 23487.47 (19842.66 to 27588.92) | 68946.16 (58375.98 to 80574.35) | 471.78 (405.66 to 543.72) | 473.76 (407.23 to 545.96) | 958.55 (502.37 to 1662.26) | 2781.91 (1446.15 to 4778.07) | 19.36 (9.72 to 34.57) | 19.53 (10.01 to 34.55) | |
| Tajikistan | 545.97 (413.37 to 694.97) | 1109.22 (839.99 to 1409.46) | 168.1 (127.32 to 213.96) | 69.51 (54.66 to 86.17) | 5132.24 (4423.18 to 5924.22) | 10196.12 (8796.86 to 11759.59) | 519.93 (431.26 to 621.76) | 468.47 (402.8 to 539.91) | 207.95 (108.52 to 360.07) | 414.51 (218.08 to 717.12) | 19.59 (10.13 to 34.88) | 19.42 (9.9 to 34.18) | |
| Thailand | 4101.62 (3224.89 to 5077.89) | 14539.56 (11434.45 to 18005.16) | 102.18 (80.4 to 126.46) | 74.55 (56.9 to 94.95) | 27112.48 (23239.71 to 31330.62) | 96795.31 (82962.11 to 111908.23) | 467.13 (401.9 to 538.36) | 519.09 (429.05 to 623.88) | 1101.94 (575.66 to 1894.85) | 3927.94 (2037.6 to 6869.27) | 21.28 (10.89 to 37.63) | 21.38 (11.05 to 38) | |
| Timor-Leste | 27.5 (21.6 to 34.08) | 110.22 (86.77 to 136.46) | 100.84 (79.33 to 124.76) | 69.68 (54.78 to 86.37) | 180.81 (154.86 to 208.8) | 726.33 (621.81 to 840.21) | 469.26 (423.8 to 520.55) | 469.78 (403.88 to 541.35) | 7.37 (3.91 to 12.91) | 29.38 (15.44 to 51.18) | 19.3 (10.05 to 33.51) | 19.42 (9.91 to 34.15) | |
| Togo | 89.88 (69.12 to 113.56) | 279.31 (214.65 to 352.77) | 66.32 (50.96 to 83.89) | 70.53 (60.11 to 82.08) | 587.65 (483.3 to 702.32) | 1824.82 (1499.75 to 2179.79) | 467.44 (402.04 to 538.72) | 471.85 (425.8 to 522.53) | 24.37 (12.35 to 43.24) | 75.7 (39.15 to 131.28) | 19.28 (10.05 to 33.32) | 19.49 (10.19 to 33.58) | |
| Tokelau | 0.2 (0.16 to 0.25) | 0.2 (0.16 to 0.26) | 105.34 (82.37 to 131.25) | 69.55 (54.68 to 86.21) | 1.35 (1.16 to 1.56) | 1.38 (1.18 to 1.6) | 467.79 (402.33 to 539.12) | 468.92 (403.28 to 540.33) | 0.06 (0.03 to 0.1) | 0.06 (0.03 to 0.1) | 19.27 (9.82 to 34.02) | 19.41 (9.94 to 33.86) | |
| Tonga | 7.03 (5.49 to 8.76) | 10.22 (7.99 to 12.74) | 105.49 (82.47 to 131.56) | 69.82 (54.88 to 86.52) | 46.97 (40.26 to 54.35) | 68.97 (59.07 to 79.81) | 473.03 (406.59 to 545.18) | 470.93 (404.88 to 542.68) | 1.92 (1.01 to 3.33) | 2.78 (1.43 to 4.82) | 19.33 (10.1 to 34.15) | 19.44 (9.99 to 34.7) | |
| Trinidad and Tobago | 149.69 (116.55 to 185.83) | 370.09 (287.66 to 459.84) | 142.77 (111.02 to 177.46) | 70 (55.01 to 86.69) | 1083.65 (927.91 to 1254.33) | 2683.33 (2297.28 to 3106.46) | 707.57 (606.66 to 819.74) | 472.32 (406 to 544.32) | 43.58 (22.97 to 74.91) | 106.36 (56.28 to 182.02) | 19.62 (9.97 to 34.47) | 19.51 (9.83 to 34.82) | |
| Tunisia | 438.57 (339.33 to 548.73) | 1221.64 (944.48 to 1528.58) | 71.27 (55.21 to 89.17) | 105.5 (82.47 to 131.53) | 2871.77 (2458.05 to 3336.73) | 8027.57 (6873.12 to 9319.19) | 1051.63 (899.84 to 1218.79) | 712.72 (610.72 to 825.02) | 119.35 (60.48 to 209.82) | 331.04 (170.37 to 587.91) | 28.73 (14.87 to 49.5) | 28.62 (14.8 to 49.78) | |
| Turkey | 4118.41 (3078.84 to 5278.14) | 11923.91 (8922.44 to 15287.09) | 100.45 (75.18 to 128.7) | 143.25 (111.52 to 178.11) | 31156.81 (26092.68 to 36856.52) | 90536.73 (75827.19 to 107683.56) | 710.96 (609.28 to 823.2) | 1047.42 (896.6 to 1213.22) | 1275.25 (651.87 to 2186.69) | 3663.02 (1887.11 to 6274.22) | 42.78 (22.37 to 74.6) | 42.09 (21.97 to 71.36) | |
| Turkmenistan | 382.5 (289.25 to 487.27) | 796.06 (601.66 to 1013.86) | 168.72 (127.67 to 214.97) | 105.84 (82.75 to 131.99) | 3613.38 (3113.92 to 4170.85) | 7453.64 (6426.87 to 8597.3) | 1044.69 (894.71 to 1209.32) | 715.49 (612.79 to 828.04) | 146.3 (77.51 to 248.42) | 301.62 (158.44 to 510.54) | 29.09 (14.87 to 51.16) | 29.18 (15.34 to 50.77) | |
| Tuvalu | 0.9 (0.7 to 1.12) | 1.41 (1.1 to 1.76) | 106.52 (83.32 to 132.86) | 143.27 (111.57 to 178.08) | 5.98 (5.11 to 6.91) | 9.45 (8.08 to 10.93) | 437.69 (360.12 to 523.79) | 1047.36 (896.85 to 1212.85) | 0.24 (0.13 to 0.42) | 0.38 (0.2 to 0.66) | 42.19 (21.84 to 72.71) | 41.85 (21.88 to 71.62) | |
| Uganda | 632.45 (482.04 to 799.2) | 1380.17 (1051.4 to 1745.52) | 84.49 (64.27 to 107.03) | 67.27 (51.65 to 85.1) | 4700.79 (3910.95 to 5558.49) | 10262.01 (8532.5 to 12130.22) | 713.12 (611.03 to 825.33) | 444.61 (365.55 to 532.09) | 194.39 (99.83 to 341.25) | 426.19 (219.43 to 745.9) | 18.07 (9.13 to 32.11) | 18.36 (9.47 to 32.01) | |
| Ukraine | 9843.71 (8558.25 to 11284.18) | 10731.39 (9333.7 to 12284.37) | 102.26 (88.84 to 117.18) | 105.37 (82.38 to 131.33) | 68303.73 (61715.51 to 75350.07) | 73972.35 (66879.83 to 81534.75) | 2082.99 (1859.96 to 2325.04) | 711.63 (609.83 to 823.57) | 2755.25 (1435.74 to 4680.43) | 2968.7 (1539.79 to 5133.45) | 28.99 (14.82 to 50.34) | 28.38 (14.64 to 49.77) | |
| United Arab Emirates | 24.53 (18.97 to 30.73) | 237 (182.96 to 296.18) | 70.9 (54.94 to 88.79) | 216.07 (173.69 to 263.69) | 160.36 (137.22 to 186.27) | 1534.65 (1312.87 to 1785.01) | 720.44 (616.49 to 833.91) | 2057.42 (1835.63 to 2296.31) | 6.63 (3.51 to 11.81) | 63.78 (32.16 to 112.2) | 82.81 (44.02 to 139.1) | 82.27 (43.85 to 139.78) | |
| United Kingdom | 25748.48 (21760.49 to 30269.57) | 35167.51 (29556.7 to 41092.24) | 212.14 (179.27 to 249.64) | 106.21 (83.04 to 132.43) | 217313.67 (196345.61 to 240226.89) | 301575.74 (273095.95 to 333435.39) | 713.04 (611 to 825.38) | 718.53 (614.96 to 831.71) | 8711.53 (4641.31 to 14496.61) | 12003.41 (6397.75 to 19932.07) | 29.23 (14.98 to 51.39) | 28.79 (15.2 to 49.9) | |
| United Republic of Tanzania | 1146.94 (899.12 to 1422.5) | 2505.15 (1962.25 to 3098.32) | 88.6 (69.33 to 110.09) | 105.26 (82.3 to 131.15) | 8762.89 (7612.92 to 9985.14) | 18973.33 (16436.78 to 21800.12) | 1050.29 (899.36 to 1216.71) | 710.53 (608.89 to 822.06) | 357.14 (188.17 to 621.94) | 782.45 (406.04 to 1380.36) | 28.83 (15.11 to 50.16) | 28.42 (14.63 to 50.09) | |
| United States of America | 14.17 (11.03 to 17.59) | 36.39 (28.34 to 45.19) | 224.46 (193.05 to 259.23) | 143.27 (111.46 to 178.22) | 102.05 (87.37 to 118.2) | 265.08 (226.86 to 306.94) | 700.66 (600.67 to 812.02) | 1048.11 (896.9 to 1214.08) | 4.13 (2.19 to 7.12) | 10.54 (5.56 to 17.81) | 42 (21.96 to 72.13) | 41.73 (22.19 to 71.4) | |
| United States Virgin Islands | 94766.57 (81560.58 to 109364.33) | 179007.14 (154561.43 to 206577.38) | 143.11 (111.35 to 177.97) | 106.6 (83.27 to 132.79) | 951939.61 (874703.82 to 1033373.28) | 1785263.55 (1639173.64 to 1942483.31) | 721 (616.88 to 834.38) | 721.74 (617.66 to 835.12) | 37822.81 (20219.42 to 62323.87) | 69810.16 (37347.43 to 114650.94) | 28.42 (14.4 to 50.78) | 28.76 (15 to 50.72) | |
| Uruguay | 992.6 (766.13 to 1245.52) | 1344.11 (1036.4 to 1688.3) | 190.13 (146.62 to 238.7) | 106.23 (83.06 to 132.39) | 8132.56 (6990.31 to 9396.05) | 11266.45 (9682.69 to 13014.19) | 1596.17 (1384.85 to 1823.61) | 718.65 (615.05 to 831.52) | 327.55 (169.28 to 551.12) | 449.41 (237.38 to 767.56) | 29.21 (15.12 to 50.78) | 28.92 (15.17 to 50.39) | |
| Uzbekistan | 2323.28 (1757.99 to 2957.58) | 5162.55 (3906.19 to 6568.43) | 168.76 (127.73 to 215.03) | 197.64 (156.07 to 245.78) | 22077.74 (19029.24 to 25480.62) | 47951 (41368.42 to 55304.41) | 711.5 (609.63 to 823.7) | 1592.12 (1382.24 to 1818.58) | 891.37 (468.28 to 1504.66) | 1936.24 (1009.59 to 3352.24) | 64.19 (34.77 to 111.43) | 63.89 (33.97 to 108.63) | |
| Vanuatu | 7.33 (5.72 to 9.13) | 20.58 (16.07 to 25.66) | 104.56 (81.65 to 130.23) | 105.84 (82.73 to 131.9) | 48.54 (41.62 to 56.21) | 136.87 (117.28 to 158.28) | 1599.23 (1386.78 to 1827.22) | 715.34 (612.67 to 827.68) | 1.99 (1.02 to 3.51) | 5.57 (2.93 to 9.66) | 28.93 (14.98 to 50.03) | 28.69 (14.77 to 49.9) | |
| Venezuela (Bolivarian Republic of) | 1626 (1303.99 to 1973.96) | 5513.76 (4403.2 to 6677.96) | 144.62 (115.93 to 175.71) | 197.55 (155.97 to 245.58) | 12286.48 (10611.99 to 14085.85) | 41762.63 (36145.12 to 47956.11) | 703.38 (602.74 to 815.42) | 1593.06 (1382.45 to 1819.87) | 495.85 (254.05 to 839.84) | 1672.33 (881.45 to 2845.54) | 64.77 (33.87 to 109.87) | 64.27 (33.56 to 109.06) | |
| Viet Nam | 5175.24 (4070.68 to 6409.3) | 12528.57 (9847.77 to 15523.29) | 103.45 (81.37 to 128.07) | 104.98 (82.05 to 130.74) | 34377.98 (29464.88 to 39741.59) | 83130.51 (71274.44 to 96043.29) | 1046.75 (895.97 to 1212.38) | 708.54 (606.97 to 820.68) | 1404.33 (730.92 to 2420.21) | 3412.93 (1762.51 to 6005.29) | 28.69 (14.7 to 49.52) | 28.69 (14.65 to 49.38) | |
| Yemen | 401.42 (310.12 to 502.63) | 1129.88 (872.86 to 1414.27) | 72.01 (55.76 to 90.17) | 142.96 (111.19 to 177.76) | 2626.63 (2249.04 to 3051.84) | 7403.99 (6338.57 to 8599.07) | 448.51 (385.83 to 517.71) | 1045.54 (894.9 to 1210.36) | 108.6 (54.95 to 192.19) | 305.58 (158.26 to 549.56) | 42.18 (22.33 to 72.67) | 41.57 (21.97 to 70.4) | |
| Zambia | 213.66 (169.43 to 261.25) | 504.07 (399.69 to 616.77) | 67.77 (53.66 to 82.99) | 67.86 (53.74 to 83.13) | 1412.95 (1215.53 to 1629.29) | 3336.4 (2869.95 to 3847.86) | 468.87 (401.34 to 544.25) | 453.24 (390.06 to 522.81) | 58.62 (30.67 to 104.22) | 137.95 (71.81 to 240.51) | 18.34 (9.43 to 32.55) | 18.47 (9.37 to 32.32) | |
| Zimbabwe | 314.73 (246.38 to 388.62) | 526.27 (411.56 to 649.9) | 65.46 (51.22 to 80.91) | 70.89 (54.95 to 88.72) | 2015.4 (1721.97 to 2326.53) | 3361.29 (2870.55 to 3881.01) | 468.87 (401.34 to 544.25) | 467.01 (399.64 to 542.5) | 83.83 (43.57 to 148.03) | 139.37 (73.28 to 246.91) | 19.38 (9.91 to 34.16) | 19.23 (10.02 to 34.29) | |

Abbreviations: ASRs, age-standardized rates; DALYs, disability-adjusted life-years; UI, uncertainty interval.

**Supplement Table 3. Decomposition analysis of changes in incidence, prevalence and DALYs rates by sex and SDI regions.**

| **Location** | **Metric** | **Overall difference** | **Aging** | **Population growth** | | **Epidemiological change** |
| --- | --- | --- | --- | --- | --- | --- |
| Global | Incidence | 733577.32 | 1004.92 (0.14%) | 801341.04 (109.24%) | -68768.64 (-9.37%) | |
| High-middle SDI | Incidence | 150763.57 | 146.19 (0.1%) | 165680.13 (109.89%) | -15062.75 (-9.99%) | |
| Middle SDI | Incidence | 216399.7 | -334.82 (-0.15%) | 218171.36 (100.82%) | -1436.84 (-0.66%) | |
| Low SDI | Incidence | 22972.39 | -87.65 (-0.38%) | 22894.55 (99.66%) | 165.49 (0.72%) | |
| High SDI | Incidence | 254914.68 | -3683.85 (-1.45%) | 263276.19 (103.28%) | -4677.65 (-1.83%) | |
| Low-middle SDI | Incidence | 88044.95 | -77.28 (-0.09%) | 86936.73 (98.74%) | 1185.49 (1.35%) | |
| Global | Prevalence | 5690511.15 | 109227.36 (1.92%) | 6355317.54 (111.68%) | -774033.75 (-13.6%) | |
| High-middle SDI | Prevalence | 1100908.56 | 21639.72 (1.97%) | 1250694.8 (113.61%) | -171425.96 (-15.57%) | |
| Middle SDI | Prevalence | 1460053.01 | 7566.75 (0.52%) | 1461491.25 (100.1%) | -9004.99 (-0.62%) | |
| Low SDI | Prevalence | 151408.17 | -69.06 (-0.05%) | 150855.77 (99.64%) | 621.45 (0.41%) | |
| High SDI | Prevalence | 2373972 | 25968.54 (1.09%) | 2402954.76 (101.22%) | -54951.3 (-2.31%) | |
| Low-middle SDI | Prevalence | 600658.87 | 2209.38 (0.37%) | 589186.31 (98.09%) | 9263.18 (1.54%) | |
| Global | DALYs | 228117.02 | 3368.63 (1.48%) | 255984.18 (112.22%) | -31235.79 (-13.69%) | |
| High-middle SDI | DALYs | 44562.1 | 616.23 (1.38%) | 50560.59 (113.46%) | -6614.72 (-14.84%) | |
| Middle SDI | DALYs | 59485.02 | 114.77 (0.19%) | 59855.23 (100.62%) | -484.97 (-0.82%) | |
| Low SDI | DALYs | 6239 | -12.75 (-0.2%) | 6201.07 (99.39%) | 50.68 (0.81%) | |
| High SDI | DALYs | 93317.23 | 496.19 (0.53%) | 95732.93 (102.59%) | -2911.89 (-3.12%) | |
| Low-middle SDI | DALYs | 24373 | 35.61 (0.15%) | 23972.36 (98.36%) | 365.03 (1.5%) | |

Abbreviations: DALYs, disability-adjusted life-years; SDI, Socio-demographic Index.

**Supplement Table 4. Cross-country inequality analysis of DALYs for elderly** **atopic dermatitis.**

| **Metric** | **Year** | **Atopic dermatitis** |
| --- | --- | --- |
| **All included** | | |
| **Slope index** | 1990 | 45.46 (38.14, 52.77) |
|  | 2021 | 35.6 (28.98, 42.22) |
| **Concentration index** | 1990 | 0.29 (0.31, 0.26) |
|  | 2021 | 0.26 (0.3, 0.23) |
| **high SDI** |  |  |
| **Slope index** | 1990 | 42.11 (24.4, 59.81) |
|  | 2021 | 36.71 (20.67, 52.74) |
| **Concentration index** | 1990 | 0.05 (0.09, 0) |
|  | 2021 | 0.02 (0.07, -0.02) |
| **High-middle SDI** | | |
| **Slope index** | 1990 | 32.59 (2.77, 62.42) |
|  | 2021 | 8.03 (-16.03, 32.09) |
| **Concentration index** | 1990 | 0.18 (0.23, 0.13) |
|  | 2021 | 0.15 (0.2, 0.1) |
| **Middle SDI** | | |
| **Slope index** | 1990 | 14.17 (1.85, 26.49) |
|  | 2021 | 3.24 (-8.34, 14.82) |
| **Concentration index** | 1990 | 0.05 (0.12, -0.02) |
|  | 2021 | -0.08 (-0.02, -0.15) |
| **Low-middle SDI** | | |
| **Slope index** | 1990 | 6.86 (-1.32, 15.04) |
|  | 2021 | 8.67 (0.95, 16.39) |
| **Concentration index** | 1990 | 0.03 (0.08, -0.02) |
|  | 2021 | 0 (0.04, -0.05) |
| **Low SDI** | | |
| **Slope index** | 1990 | -0.01 (-0.96, 0.94) |
|  | 2021 | 0.79 (-0.23, 1.81) |
| **Concentration index** | 1990 | 0.03 (0.08, -0.02) |
|  | 2021 | 0.05 (0.09, 0) |

Abbreviations: DALYs, disability-adjusted life-years; SDI, Socio-demographic Index.

**Supplement Table 5. Frontier analysis based on SDI and DALYs attributable to elderly atopic dermatitis in 204 countries and territories.**

| **Location** | **SDI** | **Rate of Incidence (95% CI)** | **Frontier Incidence** | **Effective difference** |
| --- | --- | --- | --- | --- |
| Afghanistan | 0.337199998 | 72.25 (55.84 to 90.4) | 12.33 | 6.86 |
| Albania | 0.706849791 | 85.69 (67.49 to 105.16) | 10.96 | 11.6 |
| Algeria | 0.659500924 | 70.83 (53.13 to 91.44) | 10.96 | 8.01 |
| American Samoa | 0.723727533 | 105.37 (82.38 to 131.33) | 10.97 | 17.41 |
| Andorra | 0.869444113 | 197.05 (155.67 to 244.77) | 10.97 | 52.51 |
| Angola | 0.453721949 | 60.6 (47.65 to 74.62) | 11.63 | 4.18 |
| Antigua and Barbuda | 0.749886887 | 142.7 (110.97 to 177.33) | 10.96 | 30.58 |
| Argentina | 0.723122973 | 190.07 (146.55 to 238.57) | 10.96 | 51.62 |
| Armenia | 0.701833194 | 167.99 (127.2 to 213.52) | 10.95 | 52.42 |
| Australia | 0.844252814 | 145.15 (112.42 to 181.34) | 10.96 | 28.09 |
| Austria | 0.853837004 | 197.74 (156.09 to 245.92) | 10.96 | 52.93 |
| Azerbaijan | 0.694851274 | 167.62 (127 to 213.02) | 10.95 | 52.37 |
| Bahamas | 0.805020668 | 143.36 (111.63 to 178.23) | 10.96 | 30.93 |
| Bahrain | 0.753043204 | 71 (55.07 to 88.87) | 10.96 | 8.03 |
| Bangladesh | 0.492420885 | 72.91 (57.14 to 90) | 11.05 | 8.42 |
| Barbados | 0.746748764 | 143.16 (111.46 to 177.93) | 10.96 | 30.98 |
| Belarus | 0.784484711 | 100.33 (85.48 to 117.45) | 10.96 | 17.05 |
| Belgium | 0.853654016 | 197.61 (156 to 245.7) | 10.97 | 52.45 |
| Belize | 0.610229002 | 141.88 (110.28 to 176.09) | 10.96 | 30.67 |
| Benin | 0.373486574 | 70.07 (55.06 to 86.77) | 12.29 | 7.24 |
| Bermuda | 0.821365422 | 143.25 (111.52 to 178.11) | 10.97 | 31.13 |
| Bhutan | 0.473062378 | 73.15 (57.33 to 90.31) | 11.06 | 8.51 |
| Bolivia (Plurinational State of) | 0.599010799 | 133.98 (108.93 to 163.74) | 10.97 | 26.81 |
| Bosnia and Herzegovina | 0.723077893 | 86.4 (68.03 to 106.07) | 10.94 | 11.67 |
| Botswana | 0.642721629 | 66.14 (51.69 to 81.72) | 10.96 | 6.65 |
| Brazil | 0.653043887 | 169.41 (147.47 to 194.22) | 10.97 | 45.37 |
| Brunei Darussalam | 0.810234367 | 200.24 (154.08 to 250.92) | 10.94 | 56.07 |
| Bulgaria | 0.768150939 | 86.61 (68.19 to 106.33) | 10.96 | 11.81 |
| Burkina Faso | 0.285118402 | 69.95 (54.97 to 86.63) | 12.33 | 7.18 |
| Burundi | 0.289374365 | 67.99 (53.85 to 83.29) | 12.32 | 6.58 |
| Cabo Verde | 0.533534539 | 70.69 (55.53 to 87.51) | 11.01 | 8.79 |
| Cambodia | 0.473621491 | 103.96 (81.78 to 128.71) | 11.19 | 16.7 |
| Cameroon | 0.479691223 | 78.17 (60.99 to 97.7) | 11.06 | 12.04 |
| Canada | 0.87317068 | 216.77 (173.66 to 264.48) | 10.97 | 72.49 |
| Central African Republic | 0.30916769 | 60.81 (47.81 to 74.88) | 12.33 | 3.45 |
| Chad | 0.240436019 | 68.93 (54.18 to 85.51) | 15.57 | 3.59 |
| Chile | 0.771514716 | 190.06 (146.54 to 238.55) | 10.96 | 51.2 |
| China | 0.72162976 | 99.8 (85.73 to 115.42) | 10.97 | 15.21 |
| Colombia | 0.655442913 | 128.56 (106.72 to 153.07) | 10.95 | 24.59 |
| Comoros | 0.475978688 | 68.77 (54.46 to 84.25) | 11.3 | 7.71 |
| Congo | 0.583075236 | 49.34 (38.94 to 61.44) | 10.97 | 1.09 |
| Cook Islands | 0.779109955 | 105.5 (82.47 to 131.53) | 10.96 | 17.65 |
| Costa Rica | 0.700340477 | 128.37 (106.58 to 152.83) | 10.96 | 24.42 |
| Coted'Ivoire | 0.425941883 | 69.41 (54.59 to 86.05) | 12.14 | 7.15 |
| Croatia | 0.798341027 | 1. 3 (67.96 to 105.93) | 10.96 | 11.71 |
| Cuba | 0.668729864 | 142.89 (111.18 to 177.58) | 10.96 | 31.04 |
| Cyprus | 0.835630545 | 180.35 (136.91 to 231.1) | 10.96 | 43.72 |
| Czechia | 0.828450433 | 86.13 (67.82 to 105.71) | 10.94 | 11.55 |
| Democratic People's Republic of Korea | 0.569854634 | 120.73 (94.66 to 150.04) | 11 | 23.46 |
| Democratic Republic of the Congo | 0.383179849 | 60.67 (47.71 to 74.72) | 12.31 | 3.48 |
| Denmark | 0.896424204 | 237.31 (177.39 to 304.67) | 10.96 | 75.24 |
| Djibouti | 0.487958371 | 67.83 (53.71 to 83.19) | 11.06 | 7.75 |
| Dominica | 0.746967185 | 142.62 (110.81 to 177.24) | 10.96 | 30.81 |
| Dominican Republic | 0.619388201 | 142.64 (110.93 to 177.23) | 10.96 | 30.86 |
| Ecuador | 0.661017053 | 135.19 (107.5 to 165.09) | 10.95 | 27.52 |
| Egypt | 0.606787094 | 46.45 (36.27 to 58.07) | 10.93 | 0 |
| El Salvador | 0.563775188 | 129.03 (107.09 to 153.74) | 10.96 | 24.9 |
| Equatorial Guinea | 0.657857456 | 60.78 (47.78 to 74.77) | 10.97 | 4.84 |
| Eritrea | 0.403863943 | 69.51 (55.02 to 85.27) | 12.32 | 6.8 |
| Estonia | 0.844917787 | 146.55 (122.08 to 173.38) | 10.96 | 47.47 |
| Eswatini | 0.585459713 | 66.69 (52.11 to 82.51) | 10.99 | 6.77 |
| Ethiopia | 0.358823295 | 63.7 (54.46 to 74.19) | 12.33 | 4.5 |
| Fiji | 0.675051631 | 106.2 (83.03 to 132.46) | 10.96 | 17.73 |
| Finland | 0.859831368 | 197.68 (156.03 to 245.81) | 10.96 | 52.62 |
| France | 0.838364875 | 249.97 (189.38 to 321.84) | 10.97 | 82.68 |
| Gabon | 0.634691393 | 74.2 (57.32 to 93.43) | 10.96 | 9.81 |
| Gambia | 0.40971416 | 69.87 (54.91 to 86.57) | 12.31 | 7.13 |
| Georgia | 0.732473604 | 168.13 (128.03 to 213.89) | 10.96 | 52.77 |
| Germany | 0.902957091 | 199.33 (155.91 to 249.02) | 10.96 | 53.8 |
| Ghana | 0.56493039 | 62.09 (48.57 to 77.02) | 10.98 | 5.4 |
| Greece | 0.791854408 | 176.44 (134.38 to 226.84) | 10.96 | 41.85 |
| Greenland | 0.826210336 | 216.07 (173.69 to 263.69) | 10.97 | 71.31 |
| Grenada | 0.668993028 | 142.95 (110.96 to 177.67) | 10.97 | 30.71 |
| Guam | 0.803982203 | 105.84 (82.75 to 131.99) | 10.96 | 18.23 |
| Guatemala | 0.539972424 | 128.21 (106.46 to 152.64) | 11 | 23.86 |
| Guinea | 0.336401293 | 69.35 (54.55 to 86) | 12.29 | 7.01 |
| Guinea-Bissau | 0.353109621 | 70.19 (55.16 to 86.9) | 12.29 | 7.25 |
| Guyana | 0.650812335 | 143.06 (111.36 to 177.83) | 10.96 | 30.15 |
| Haiti | 0.448278285 | 142.67 (111.04 to 177.22) | 11.08 | 30.23 |
| Honduras | 0.513037248 | 128.25 (106.48 to 152.69) | 11.02 | 24.12 |
| Hungary | 0.790754768 | 116.67 (90.38 to 147.04) | 10.96 | 24.27 |
| Iceland | 0.87636168 | 215.07 (163.37 to 275.8) | 10.95 | 62.58 |
| India | 0.575401649 | 74.1 (63.4 to 86.14) | 11 | 8.49 |
| Indonesia | 0.656868336 | 102.4 (87.45 to 118.37) | 10.96 | 16.45 |
| Iran (Islamic Republic of) | 0.697207398 | 79 (66.25 to 93.55) | 10.97 | 11.05 |
| Iraq | 0.662626231 | 71.79 (55.55 to 89.85) | 10.96 | 8.29 |
| Ireland | 0.87375385 | 197.58 (156.01 to 245.68) | 10.97 | 53.06 |
| Israel | 0.809011652 | 197.86 (156.18 to 246.19) | 10.96 | 53.24 |
| Italy | 0.805773534 | 232.53 (196.06 to 274.15) | 10.96 | 70.39 |
| Jamaica | 0.683263064 | 142.57 (110.85 to 177.14) | 10.96 | 30.93 |
| Japan | 0.871241813 | 230.87 (193.93 to 271.79) | 10.96 | 75.04 |
| Jordan | 0.725307227 | 69.95 (53 to 89.04) | 10.96 | 7.67 |
| Kazakhstan | 0.725144495 | 168.28 (127.36 to 214.2) | 10.97 | 53.03 |
| Kenya | 0.523768077 | 62.9 (53.73 to 73.09) | 11 | 5.5 |
| Kiribati | 0.527186583 | 106.99 (83.51 to 133.35) | 11.01 | 18.06 |
| Kuwait | 0.846651055 | 74.21 (55.7 to 96.11) | 10.96 | 9.43 |
| Kyrgyzstan | 0.603979328 | 167.94 (127.15 to 213.58) | 10.97 | 52.95 |
| Lao People's Democratic Republic | 0.489136091 | 101.69 (80.03 to 125.84) | 11.05 | 16.48 |
| Latvia | 0.830663516 | 53.5 (46.12 to 61.35) | 10.95 | 0.88 |
| Lebanon | 0.744746351 | 72.03 (55.69 to 90.12) | 10.96 | 8.41 |
| Lesotho | 0.510393066 | 66.8 (52.18 to 82.63) | 11.04 | 6.68 |
| Liberia | 0.352442452 | 69.43 (54.58 to 86.06) | 12.33 | 6.86 |
| Libya | 0.725771399 | 71.56 (55.36 to 89.57) | 10.96 | 8.35 |
| Lithuania | 0.856484049 | 100.11 (85.31 to 117.13) | 10.96 | 16.94 |
| Luxembourg | 0.884428955 | 197.41 (155.86 to 245.37) | 10.96 | 52.92 |
| Madagascar | 0.400246943 | 68.43 (54.18 to 83.84) | 12.32 | 6.61 |
| Malawi | 0.384553634 | 69.01 (54.62 to 84.58) | 12.32 | 6.76 |
| Malaysia | 0.742523828 | 101.25 (79.68 to 125.24) | 10.96 | 16.16 |
| Maldives | 0.650886627 | 100.32 (78.97 to 124.12) | 10.96 | 16.21 |
| Mali | 0.268579941 | 74.55 (56.9 to 94.95) | 15.59 | 5.79 |
| Malta | 0.801585034 | 197.55 (155.96 to 245.62) | 10.96 | 53.11 |
| Marshall Islands | 0.574091128 | 105.2 (82.2 to 131.1) | 10.99 | 17.45 |
| Mauritania | 0.4989451 | 69.51 (54.66 to 86.17) | 11.06 | 8.36 |
| Mauritius | 0.718260446 | 102.43 (80.59 to 126.8) | 10.97 | 16.43 |
| Mexico | 0.664575304 | 112.44 (98.25 to 128.47) | 10.96 | 17.7 |
| Micronesia (Federated States of) | 0.587534967 | 106.09 (82.95 to 132.29) | 10.98 | 17.87 |
| Monaco | 0.908262831 | 197.55 (155.97 to 245.58) | 10.96 | 53.31 |
| Mongolia | 0.617621565 | 168.45 (127.47 to 214.28) | 10.96 | 53.13 |
| Montenegro | 0.795800584 | 86.23 (67.9 to 105.84) | 10.97 | 11.72 |
| Morocco | 0.562698301 | 71.56 (55.41 to 89.53) | 11 | 8.26 |
| Mozambique | 0.326462614 | 68.96 (54.58 to 84.55) | 12.33 | 6.6 |
| Myanmar | 0.53390084 | 103.18 (81.19 to 127.7) | 10.98 | 16.75 |
| Namibia | 0.617564872 | 66.33 (51.85 to 81.99) | 10.96 | 6.81 |
| Nauru | 0.625177834 | 106.6 (83.27 to 132.79) | 10.96 | 17.8 |
| Nepal | 0.433174635 | 73.5 (57.62 to 90.8) | 11.75 | 7.74 |
| Netherlands | 0.888464256 | 197.53 (155.96 to 245.59) | 10.96 | 52.86 |
| New Zealand | 0.849442499 | 187.09 (158.9 to 218.31) | 10.96 | 48.39 |
| Nicaragua | 0.523958472 | 128.57 (106.73 to 153.09) | 11.01 | 24.28 |
| Niger | 0.168072774 | 69.68 (54.78 to 86.37) | 16.63 | 2.79 |
| Nigeria | 0.503390833 | 70.53 (60.11 to 82.08) | 11.06 | 8.43 |
| Niue | 0.72622205 | 106.21 (83.04 to 132.43) | 10.97 | 17.82 |
| North Macedonia | 0.750629703 | 85.76 (67.53 to 105.26) | 10.97 | 11.35 |
| Northern Mariana Islands | 0.771535213 | 104.98 (82.05 to 130.74) | 10.96 | 17.74 |
| Norway | 0.91613281 | 212.82 (179.09 to 250.61) | 10.96 | 59.9 |
| Oman | 0.773391602 | 69.77 (52.55 to 89.41) | 10.96 | 7.63 |
| Pakistan | 0.504028689 | 73.35 (62.77 to 85.2) | 11.06 | 8.27 |
| Palau | 0.754046931 | 105.26 (82.3 to 131.15) | 10.94 | 17.47 |
| Palestine | 0.631011665 | 73.9 (56.62 to 93.43) | 10.96 | 9.15 |
| Panama | 0.708864828 | 127.91 (106.12 to 152.24) | 10.97 | 24.39 |
| Papua New Guinea | 0.417797443 | 104.63 (81.7 to 130.33) | 11.25 | 17.24 |
| Paraguay | 0.635718099 | 170.06 (138.1 to 204.57) | 10.96 | 47.59 |
| Peru | 0.662054037 | 132.31 (106.14 to 160.72) | 10.94 | 26.61 |
| Philippines | 0.651219329 | 103.27 (88.52 to 119.77) | 10.96 | 16.62 |
| Poland | 0.812042809 | 86.88 (74.82 to 100.5) | 10.96 | 11.8 |
| Portugal | 0.744151851 | 197.99 (156.28 to 246.41) | 10.97 | 52.94 |
| Puerto Rico | 0.825525847 | 143.27 (111.57 to 178.08) | 10.97 | 30.88 |
| Qatar | 0.846860584 | 70.27 (54.49 to 87.86) | 10.94 | 7.9 |
| Republic of Korea | 0.886675267 | 206.73 (158.7 to 261.32) | 10.96 | 60.89 |
| Republic of Moldova | 0.732214875 | 99.88 (85.18 to 116.83) | 10.96 | 16.88 |
| Romania | 0.768453864 | 63.98 (50.76 to 78.72) | 10.96 | 4.17 |
| Russian Federation | 0.808536005 | 130.79 (114.75 to 148.87) | 10.96 | 34.54 |
| Rwanda | 0.435588706 | 50.97 (40.09 to 63.03) | 12.02 | 0.44 |
| Saint Kitts and Nevis | 0.754987055 | 143.27 (111.46 to 178.22) | 10.97 | 30.76 |
| Saint Lucia | 0.672509735 | 142.7 (110.97 to 177.34) | 10.96 | 30.46 |
| Saint Vincent and the Grenadines | 0.637195963 | 141.93 (110.27 to 176.17) | 10.97 | 30.3 |
| Samoa | 0.593392769 | 105.6 (82.57 to 131.68) | 10.98 | 17.75 |
| San Marino | 0.888005474 | 197.64 (156.07 to 245.78) | 10.94 | 52.94 |
| Sao tome and Principe | 0.505413747 | 70 (55.01 to 86.69) | 11.05 | 8.45 |
| Saudi Arabia | 0.815143493 | 70.33 (54.56 to 88.04) | 10.96 | 7.94 |
| Senegal | 0.408054193 | 69.82 (54.88 to 86.52) | 12.29 | 7.15 |
| Serbia | 0.792416294 | 86.21 (67.88 to 105.82) | 10.95 | 11.59 |
| Seychelles | 0.730150775 | 101.87 (80.15 to 126.11) | 10.96 | 16.29 |
| Sierra Leone | 0.358665881 | 69.55 (54.68 to 86.21) | 12.33 | 7.08 |
| Singapore | 0.856097766 | 174.23 (134.19 to 220.7) | 10.96 | 42.96 |
| Slovakia | 0.81061053 | 86.51 (68.11 to 106.19) | 10.97 | 11.7 |
| Slovenia | 0.842430731 | 85.81 (67.58 to 105.31) | 10.95 | 11.46 |
| Solomon Islands | 0.429360316 | 105.28 (82.32 to 131.21) | 11.42 | 17.36 |
| Somalia | 0.077688109 | 69.33 (54.89 to 85.01) | 18.96 | 0.19 |
| South Africa | 0.679626598 | 66.65 (57.08 to 77.46) | 10.96 | 6.64 |
| South Sudan | 0.278371125 | 67.86 (53.74 to 83.13) | 12.39 | 6.08 |
| Spain | 0.769283698 | 187.33 (141.63 to 240.97) | 10.96 | 47.08 |
| Sri Lanka | 0.701534935 | 102.89 (80.04 to 127.84) | 10.97 | 16.51 |
| Sudan | 0.541949735 | 70.89 (54.95 to 88.72) | 10.97 | 8.26 |
| Suriname | 0.633665739 | 143.19 (111.44 to 178.06) | 10.97 | 30.6 |
| Sweden | 0.886880299 | 199.02 (170.17 to 230.37) | 10.96 | 52.87 |
| Switzerland | 0.933059111 | 197.55 (155.96 to 245.61) | 10.96 | 52.66 |
| Syrian Arab Republic | 0.623004075 | 71.32 (55.19 to 89.27) | 10.95 | 8.36 |
| Taiwan (Province of China) | 0.874747053 | 150.26 (114 to 190.16) | 10.96 | 37.95 |
| Tajikistan | 0.541511187 | 167.06 (126.75 to 212.15) | 10.99 | 51.53 |
| Thailand | 0.682547933 | 102.29 (80.51 to 126.59) | 10.95 | 16.7 |
| Timor-Leste | 0.444667619 | 101.36 (79.73 to 125.43) | 11.07 | 16.1 |
| Togo | 0.408533695 | 67.27 (51.65 to 85.1) | 12.31 | 6.06 |
| Tokelau | 0.686425621 | 105.84 (82.73 to 131.9) | 10.97 | 17.72 |
| Tonga | 0.626349936 | 105.92 (82.82 to 132.08) | 10.97 | 17.84 |
| Trinidad and Tobago | 0.768763254 | 142.74 (111.01 to 177.39) | 10.96 | 30.33 |
| Tunisia | 0.682432216 | 71.7 (55.48 to 89.71) | 10.96 | 8.49 |
| Turkey | 0.712692673 | 100.51 (75.24 to 128.85) | 10.96 | 19.98 |
| Turkmenistan | 0.682160776 | 167.93 (127.17 to 213.55) | 10.96 | 52.82 |
| Tuvalu | 0.576620529 | 106.23 (83.06 to 132.39) | 10.99 | 17.93 |
| Uganda | 0.423261181 | 85.28 (64.87 to 108.13) | 12.12 | 14.33 |
| Ukraine | 0.760773913 | 101.03 (87.87 to 115.66) | 10.97 | 16.97 |
| United Arab Emirates | 0.849317734 | 68.46 (53.01 to 85.6) | 10.95 | 7.49 |
| United Kingdom | 0.859000182 | 211.91 (178.22 to 247.77) | 10.96 | 60.09 |
| United Republic of Tanzania | 0.446568273 | 87.55 (68.51 to 108.45) | 11.81 | 15.67 |
| United States Virgin Islands | 0.821830853 | 142.96 (111.19 to 177.76) | 10.94 | 30.63 |
| United States of America | 0.862448354 | 227.74 (196.59 to 262.84) | 10.97 | 77.89 |
| Uruguay | 0.719283445 | 190.15 (146.64 to 238.74) | 10.96 | 51.68 |
| Uzbekistan | 0.662621694 | 167.45 (126.91 to 212.77) | 10.96 | 52.08 |
| Vanuatu | 0.473100706 | 105.55 (82.52 to 131.59) | 11.33 | 17.49 |
| Venezuela (Bolivarian Republic of) | 0.596513059 | 144.9 (115.8 to 175.39) | 10.97 | 33.09 |
| Viet Nam | 0.627933721 | 103.28 (81.24 to 127.87) | 10.97 | 17.2 |
| Yemen | 0.450376375 | 71.65 (55.44 to 89.67) | 11.06 | 8.36 |
| Zambia | 0.505948954 | 68.66 (54.37 to 84.15) | 11.05 | 7.85 |
| Zimbabwe | 0.473819486 | 66.32 (51.87 to 81.96) | 11.06 | 6.68 |

Abbreviations: CI, confidence interval; DALYs, disability-adjusted life-years; SDI, Socio-demographic Index.

**Supplement Table 6. The predicted case number and ASR of incidence, prevalence, and DALYs of elderly atopic dermatitis from 2022 to 2050 globally.**

| **Incidence** | | | | | | **Prevalence** | | | | | | **DALYs** | | | | | |
| --- | --- | --- | --- | --- | --- | --- | --- | --- | --- | --- | --- | --- | --- | --- | --- | --- | --- |
| **2022 rate (95% CI)** | **2050 rate (95% CI)** | **Rate change** | **2022 number (95% CI)** | **2050 number (95% CI)** | **Number change** | **2022 rate (95% CI)** | **2050 rate (95% CI)** | **Rate change** | **2022 number (95% CI)** | **2050 number (95% CI)** | **Number change** | **2022 rate (95% CI)** | **2050 rate (95% CI)** | **Rate change** | **2022 number (95% CI)** | **2050 number (95% CI)** | **Number change** |
| 128.34 (96.92 to 159.77) | 136.07 (1.55 to 294.94) | 6.02% | 1449654.55 (1095460.97 to 1803848.14) | 2915200.49 (29512.24 to 6458070.01) | 101.10% | 1017.38 (986.3 to 1048.46) | 1060.29 (231.4 to 1894.87) | 4.22% | 11412389.31 (11062813.44 to 11761965.18) | 23173196.27 (5581248.5 to 40868707.1) | 103.05% | 44.85 (22.29 to 67.43) | 40.74 (6.19 to 77.27) | -0.16% | 457538.75 (68042.38 to 869732.71) | 895010.9 (0 to 4228056.23) | 95.61% |

Abbreviations: ASR, age-standardized rate; CI, confidence interval; DALYs, disability-adjusted life-years.
